# Systematic review of dietary salt reduction policies: Evidence for an effectiveness hierarchy?

**DOI:** 10.1371/journal.pone.0177535

**Published:** 2017-05-18

**Authors:** Lirije Hyseni, Alex Elliot-Green, Ffion Lloyd-Williams, Chris Kypridemos, Martin O’Flaherty, Rory McGill, Lois Orton, Helen Bromley, Francesco P. Cappuccio, Simon Capewell

**Affiliations:** 1 Department of Public Health and Policy, Institute of Psychology, Health and Society, University of Liverpool, Liverpool, United Kingdom; 2 University of Warwick, WHO Collaborating Centre, Warwick Medical School, Coventry, United Kingdom; SOAS, University of London, UNITED KINGDOM

## Abstract

**Background:**

Non-communicable disease (NCD) prevention strategies now prioritise four major risk factors: food, tobacco, alcohol and physical activity. Dietary salt intake remains much higher than recommended, increasing blood pressure, cardiovascular disease and stomach cancer. Substantial reductions in salt intake are therefore urgently needed. However, the debate continues about the most effective approaches. To inform future prevention programmes, we systematically reviewed the evidence on the effectiveness of possible salt reduction interventions. We further compared “downstream, agentic” approaches targeting individuals with “upstream, structural” policy-based population strategies.

**Methods:**

We searched six electronic databases (CDSR, CRD, MEDLINE, SCI, SCOPUS and the Campbell Library) using a pre-piloted search strategy focussing on the effectiveness of population interventions to reduce salt intake. Retrieved papers were independently screened, appraised and graded for quality by two researchers. To facilitate comparisons between the interventions, the extracted data were categorised using nine stages along the agentic/structural continuum, from “downstream”: dietary counselling (for individuals, worksites or communities), through media campaigns, nutrition labelling, voluntary and mandatory reformulation, to the most “upstream” regulatory and fiscal interventions, and comprehensive strategies involving multiple components.

**Results:**

After screening 2,526 candidate papers, 70 were included in this systematic review (49 empirical studies and 21 modelling studies). Some papers described several interventions. Quality was variable. Multi-component strategies involving both upstream and downstream interventions, generally achieved the biggest reductions in salt consumption across an entire population, most notably 4g/day in Finland and Japan, 3g/day in Turkey and 1.3g/day recently in the UK. Mandatory reformulation alone could achieve a reduction of approximately 1.45g/day (three separate studies), followed by voluntary reformulation (-0.8g/day), school interventions (-0.7g/day), short term dietary advice (-0.6g/day) and nutrition labelling (-0.4g/day), but each with a wide range. Tax and community based counselling could, each typically reduce salt intake by 0.3g/day, whilst even smaller population benefits were derived from health education media campaigns (-0.1g/day). Worksite interventions achieved an increase in intake (+0.5g/day), however, with a very wide range. Long term dietary advice could achieve a -2g/day reduction under optimal research trial conditions; however, smaller reductions might be anticipated in unselected individuals.

**Conclusions:**

Comprehensive strategies involving multiple components (reformulation, food labelling and media campaigns) and “upstream” population-wide policies such as mandatory reformulation generally appear to achieve larger reductions in population-wide salt consumption than “downstream”, individually focussed interventions. This ‘effectiveness hierarchy’ might deserve greater emphasis in future NCD prevention strategies.

## Introduction

Non-communicable diseases (NCDs) kill over 35 million people annually. Common cancers, cardiovascular diseases, diabetes, respiratory diseases and dementia together now account for over two thirds of the entire global burden of disability and death.[1,2] These NCDs are mainly attributable to just four major risk factors. Furthermore, the contribution from poor diet exceeds the combined contribution from alcohol, tobacco and physical inactivity.[3] This poor diet mainly reflects a predominantly unhealthy global food environment, dominated by processed foods high in sugar, saturated fat, trans-fat and, crucially, salt.[3]

In the UK and other high income countries, over 70% of dietary salt is consumed in processed foods such as bread, breakfast cereals, processed meats, snack foods, soups and sauces.[4–6] This food environment contributes to excessive salt intake among adults, on average 10g/day or more,[7] far in excess of what the body actually needs.[8] High salt intake is a major risk factor for increasing blood pressure,[9–11] cardiovascular disease,[12–14] stroke,[15,16] and stomach cancer.[17–19] Moreover, a reduction in salt intake would substantially reduce this risk.[10]

WHO recommends a maximum adult salt intake of 5g/day.[20] Different strategies and policy options have been proposed to achieve this goal. Individual level interventions often involve behavioural approaches, for example dietary counselling, leaflets or medical advice. These are sometimes termed “downstream” or “agentic” interventions, and are dependent on the individual responding. [21,22] Conversely, “upstream” structural interventions take place at the population level and typically involve policies such as regulatory approaches, taxes or subsidies. Finally, intermediate interventions target subgroups in worksites, schools or communities.[23]

National salt reduction strategies were identified in 75 countries in 2015, a substantial increase from 32 in 2010.[24] However, the debate regarding the most effective and acceptable salt reduction strategy continues.

Notable policy approaches have been seen in Finland,[25] Japan,[26] and more recently, the United Kingdom.[27] In the UK, a combination of awareness campaigns, agreed target settings, voluntary reformulation from industry and population monitoring of salt consumption have led to a 1.4g per day reduction in population salt intake between 2001 and 2011 (the campaign started in 2003).[27] However, health inequalities in salt consumption have persisted.[28,29] Furthermore, the introduction of the UK Responsibility Deal in 2010 shifted emphasis to ‘downstream’ interventions, coupled with ineffective voluntary agreements and, controversially, the direct involvement of the industry in policy decisions.[30,31]

Geoffrey Rose famously advocated population wide approaches rather than targeting high-risk individuals.[32] Furthermore, there seems to be some evidence for a public health ‘effectiveness hierarchy’ whereby “upstream” structural interventions consistently achieve larger improvements in population health, are more equitable and often reduce health inequalities[33,34] compared to “downstream” agentic interventions targeting individuals, for instance in tobacco control and alcohol policies.[35,36] Emerging evidence suggests that a comparable effectiveness hierarchy might also exist for salt reduction strategies, whereby upstream interventions apparently achieve bigger reductions in salt intake.[37,38]. To test this hypothesis and hence inform future preventive health strategies, we have systematically reviewed the evidence for studies focusing on the effectiveness of salt interventions to reduce salt intake.

## Methods

### Study design

We conducted a systematic review of interventions intended to decrease population dietary salt intake. To ensure proper conduct, we adhered to the PRISMA checklist (Preferred Reporting Items for Systematic Reviews and Meta-Analyses)(S1 Table).[39] We used a narrative synthesis and formally investigated evidence to support or refute an effectiveness hierarchy. The research protocol can be found in S1 File.

### Search strategy

We first identified exemplar studies to define and refine search terms needed for targeted searches. The search strategy consisted of a combination of four sets of key words:

1) salt, sodium; 2) health promotion, nutrition education, campaigns, dietary counselling, regulation, legislation, tax, self-regulation, reformulation, social marketing, promotion, provision, labelling, marketing control, primary care advice, food industry; 3) public policy, health policy, nutrition policy, policies, interventions, strategies, initiatives, programmes, policy option, actions; and 4) effectiveness, effect, intake, consumption, reduction, cost-benefit analysis, and cardiovascular diseases.

A pilot search was conducted to determine appropriate databases, identify relevant studies and highlight potential issues to be addressed. This process identified six databases which were then used for the targeted searches: Ovid MEDLINE, Science Citation Index, SCOPUS, Cochrane Database of Systematic Reviews, The Campbell Collaboration Library of Systematic Reviews and the CRD Wider Public Health database. We searched for all studies published in the last four decades (from 1975 onwards). The final searches were conducted on 30 October 2015. All papers identified by the searches were imported into the Zotero data management programme to identify duplicates and help screen titles, abstracts and full texts as appropriate. The reference lists of included studies were scanned for potential additional papers and topic experts (FPC and SC) were also consulted for additional data sources.[40,41]

### Study selection and inclusion criteria

Studies were included if they investigated the effectiveness of specific interventions on population dietary salt intake and contained quantitative outcomes. Only studies in English were included. We included a wide range of study designs including meta-analyses, trials, observational studies and natural experiments. Empirical studies and modelling studies were analysed separately, in view of their profound differences. The retrieved studies were assessed using the PICOS approach (Participants, Interventions, Comparators, Outcomes and Study design), summarised in Table 1. The primary outcome was salt intake (g/day). Studies reporting urinary sodium excretion (mmol/day) or sodium mg/day were converted to g/day. Where necessary, we simultaneously considered studies reporting solely on salt intake data in a specific population with the corresponding studies describing the interventions during that same time period.

**Table 1:** PICOS; Inclusion/exclusion criteria.

**Participants**	
**Include**	**Exclude**
Studies for all age groups from all populations, from high-, middle- and low-income countries	Studies on animals, cells and pregnant women
**Interventions**	
Systematic Reviews and primary studies evaluating the effects of actions to promote salt reduction by government policy or adopted in specific real or experimental settings	Studies evaluating the effect of a general or specific diet
**Comparators**	
Systematic and non-systematic reviews where actions to promote salt reduction were evaluated or compared	No comparisons of different actions to promote salt reduction presented
**Outcomes**	
Primary outcome of interest was dietary salt intake (g/day). Studies including urinary sodium excretion as an outcome were converted to g/day. Secondary outcomes included changes in clinical/physiological indicators related to NCDs and behaviours associated with a healthy diet	Process evaluations reporting on implementation of interventions/policies without any quantitative outcome data; feasibility or acceptability without an assessment or primary outcomes (intake); studies on individuals as opposed to populations; data on cost only and BMI
**Study design**	
Primary studies, RCTs, Systematic Reviews (SRs), empirical observational studies, natural experiments, and modelling studies, secondary analysis, and before vs. after interventions	Commentary/opinion articles and purely qualitative evaluations with no quantitative assessment

One reviewer (LH) conducted the searches; extracted potential papers and removed duplicates. Two reviewers (LH and AEG) then independently screened titles and abstracts for eligibility using the inclusion and exclusion criteria. Full text was retrieved for all papers deemed potentially eligible and these were also screened independently by the two reviewers. Any discrepancies were resolved by consensus or by involving the senior author (SC).

### Data extraction and management

Pre-designed and pre-piloted tables were used to extract data from all included studies. To ensure that all relevant information was captured, extracted data included: first author; year of publication; funder(s); study aim(s); sample size; study design; methods; participants; policies analysed; geographical scope; length of follow-up; outcomes, effect and response; authors’ assessment of limitations and our own assessment of potential risk of bias. The sources referenced for the effect sizes used in each modelling study were also specified in the tables (recognising that some modelling studies are based on empirical studies, potentially some included in this review). This data extraction was done independently by two reviewers (LH and AEG).

### Quality assessment of included studies

Two reviewers (LH and AEG) independently assessed the methodological quality of each study (poor, fair or good). We used the National Heart, Lung and Blood Institute (NHLBI) tools specific for each research design (i.e. RCTs, cross-sectional studies, before and after studies, and systematic reviews).[42] Several questions were asked for each study design (varying from 8 to 14) and depending on the points scored, the studies were labelled as good, fair or poor. However, we also took into consideration as to which questions points were allocated. For example, if an RCT scored 10 out of 14 points, but did not conduct an intention to treat analysis, it would be rated as fair rather than good. Modelling studies were independently assessed by two modelling experts (MOF & CK) using a different tool adapted from Fattore et al. (2014).[43] Discrepancies in quality assessment were reconciled by consensus or by involving a third, senior member of the team (SC or HB).

### Data synthesis and effectiveness hierarchy continuum

The evidence was summarised as a narrative synthesis according to intervention type, ranging from downstream to upstream interventions, to facilitate comparisons between the interventions. Summary tables of the studies included in this review can be found in Tables 2–10 for empirical studies and Table 11 for modelling studies. A more detailed data extraction of these studies can be found in S2 Table. We defined UPSTREAM interventions as those targeting the entire population (not a subset, however large) and creating structural changes (effectively removing individual choice from the equation). This accorded with the Nuffield’s ladder taxonomy,[44] and with McLaren’s structural/agentic continuum.[21] Conversely, we defined DOWNSTREAM interventions as those where the principal mechanism of action is “agentic”, being dependent on an individual altering their behaviour.

**Table 2:** Dietary counselling (individuals).

Study	Study type	Geographical scope	Aim and main outcomes	Policies analysed	Relevant results	Quality assessment
Hooper et al. (2002)^45^	SR and meta-analysis of RCTs	US, Australia, New Zealand, UK	*Aim*: to assess the long term effects of advice to restrict dietary sodium in adults with and without hypertension. *Outcomes*: salt intake as measured by urinary sodium excretion	Dietary advice	*Meta-analysis (11 studies included)*. They found reductions in salt intake at both intermediate, <12 months (2.8g/day) and late follow up, 13–60 months (2.0g/day).	Good
Appel et al. (2003)^46^	Randomised trial	US	*Aim*: to determine the effect on BP of 2 multicomponent, behavioral interventions *Outcomes*: salt intake as measured by urinary sodium excretion	Dietary advice	Only the reduction in the established group differed significantly from that of advice only group. 24-hour dietary recall data indicated both behavioral interventions significantly reduced sodium intake in comparison with advice only group (P value = 0.01). Advice group • Baseline = 10.0g/day • 6 months = 8.8g/day • Mean difference = -1.2g/day Intervention group • Established: mean difference = -1.82 g/day • Established + DASH: mean difference = -1.83 g/day	Good
Brunner et al. (1997)^47^	Meta-analysis of RCTs	UK, US, Netherlands and Australia	*Aim*: to evaluate the effectiveness of dietary advice in primary prevention of chronic disease. *Outcomes*: salt intake	Dietary advice	Overall mean net reduction of 1.8g/day which is a 20% reduction in salt intake. The heterogeneity test was highly significant (P < .0005) for the 3- to 6-month trials, with a net reduction of 3.4 (95% CI = 45, 72) g/day. Summary effect of the two trials with SE was somewhat larger at 9–18 months than at 3–6 months.	Fair
Francis & Taylor (2009)^48^	Randomised control group study	US	*Aim*: to implement a health-healthy diet-education programme. *Outcomes*: salt intake	Dietary counselling	Intervention salt consumption decreased significantly (P0.020) from record 1 to record 3. The reduction in control group participants’ sodium intake was not significant *Intervention*: (Mean ± SEM (g/day); P-value) • Record 1: 7.0 ± 0.5; 0.020e • Record 2: 5.9 ± 0.3; 0.067 • Record 3: 5.9 ± 0.4; 0.937 *Control* (Mean ± SEM (g/day), P-value) • Record 1: 6.2 ± 0.5; 0.323 • Record 2: 6.1 ± 0.4; 0.880 • Record 3: 5.7 ± 0.4; 0.284 Mean effect size:- 0.6g/day	Fair
Parekh et al. (2012)^49^	RCT	Australia	*Aim*: to evaluate the effectiveness of a minimal intervention on multiple lifestyle factors including diet using computer tailored feedback. *Outcomes*: salt intake (%)	Health promotion–computer tailored advice	*Salt (%)* Intervention +5.43 net change. Control +1.23 net change. Significant changes between groups were observed for reduced salt intake (OR 1.19, CI 1.05–1.38). The intervention group were 20% more likely to reduce salt intake	Fair
Petersen et al. (2013)^50^	RCT	Australia	*Aim*: to investigate whether urinary sodium excretion can be reduced by educating people with T2DM to read food labels and choose low sodium products. *Outcomes*: salt intake	Nutrition education	Baseline reported salt intake: 6.8 ± 3.2 g/day Intervention • Baseline: 10.0 ± 0.7 • 3 months: 10.1 ± 0.7 • Change: +0.06 ± 0.9 *Control* • Baseline: 9.6 ± 0.9 • 3 months: 9.3 ± 0.7 • Change: -0.3 ± 0.8 There was no between group difference (p > 0.05)	Fair
Kokanović et al. (2014)^51^	Before and after study	Croatia	*Aim*: to assess eating habits of adolescent population diagnosed with one or more cardiovascular risks before and after two months of individual dietary intervention *Outcomes*: salt intake	Nutrition education	Difference in intake on initial and control examination statistically significant for intake of sodium p = 0.013. *Salt intake g/day*. Initial examination: 18.9d/day; Control examination: 15.4g/day; Difference: -3.5g/day (= -18.8%)	Fair
Heino et al. (2000)^52^	Prospective randomized trial	Finland	*Aim*: to examine sodium intake of 1-5-y-old children in a CHD prevention trial, focused on dietary fat modification. *Outcomes*: salt intake	Dietary counselling	Intervention children (+1.5g/day) • 13 months: 4.1 ± 1.2 • 3 years: 4.9 ± 1.2 • 5 years: 5.6 ± 1.3 Control children (+1.6g/day) • 13 months: 3.9 ± 1.4 • 3 years: 4.7 ± 1.3 • 5 years: 5.5 ± 1.4 No significant differences between the intervention and control group found	Poor
Wang et al. (2013)^53^	RCT	US	*Aim*: one year dietary intervention study to examine patterns and amount of daily sodium intake among participants with metabolic syndrome *Outcomes*: salt intake	Dietary counselling	Intervention arm at one year follow-up found participants who consumed sodium greater than 5.8g/day declined from 75% at baseline to 59%. Those consumed higher than 3.8g/day declined from 96% (at baseline) to 85%. Average salt intake decreased from 7.5 g/day at baseline to 6.4 g/day at one-year (P<0.001). At one-year visit, salt intake was consistently reduced; significant difference only observed between males (7.6± 0.4 g/day) and females (6.0 ± 0.2 g/day; p < 0.001)	Poor

**Table 3:** Dietary counselling (worksite/schools).

Study	Study type	Geographical scope	Aim and main outcomes	Policies analysed	Relevant results	Quality assessment
He et al. (2015)^40^	Cluster RCT	China	*Aim*: to determine whether an education programme targeted at schoolchildren could lower salt intake in children and their families *Outcomes*: salt intake as measured by urinary excretion	Health education	At baseline, the mean salt intake in children was 7.3 (SE 0.3) g/day in the intervention group and 6.8 (SE 0.3) g/day in the control group. The mean effect on salt intake for intervention versus control group was −1.9 g/day (95% confidence interval −2.6 to −1.3 g/day; P<0.001). In adult family members the salt intakes were 12.6 (SE 0.4) and 11.3 (SE 0.4) g/day, respectively. During the study there was a reduction in salt intake in the intervention group, whereas in the control group salt intake increased. The mean effect on salt intake for intervention versus control group was −2.9 g/day (−3.7 to −2.2 g/day; P<0.001)	Good
Cotter et al. (2013)^57^	School based RCT	Portugal	*Aim*: to examine the influence on salt intake and blood pressure of three different educational interventions for 6 months *Outcomes*: salt intake as measured by urinary sodium excretion	Nutrition education	*Baseline*: mean salt intake of 7.8 ± 2.5 g per day. Estimated salt intake (g/d): CRT • Baseline: 7.7 ± 2.0 • Final: 7.4 ± 3.0 • Change: 0.35 ± 2.42 THEOR • Baseline: 8.1 ± 3.0 • Final: 7.5 ± 3.0 • Change: 0.60 ± 3.24 PRACT • Baseline: 7.5 ± 2.4 • Final: 6.4 ± 2.2 • Change: 1.08 ± 2.47*	Fair
Katz et al. (2011)^58^	School based RCT	US	*Aim*: to evaluate the effects of a nutrition education programme in distinguishing between healthful and less healthful choices in diverse food categories. *Outcomes*: salt intake	Nutrition education	There were no statistically significant improvements in dietary patterns from baseline between the intervention (-0.23g/day) and control groups (-0.04g/day) for salt intake (p = .44)	Poor
Aldana et al. (2005)^59^	RCT	US	*Aim*: to determine behavioral and clinical impact of a worksite chronic disease prevention program *Outcomes*: salt intake	Health education	*Intervention group (salt g/day)* • Baseline: 7.5 • ∆6 weeks: -0.5 • ∆6 months: -1.7 *Control group (salt g/day)* • Baseline: 6.3 • ∆6 weeks: -0.5 • ∆6 months: -0.5 Significant differences in mean change scores were not observed at 6 weeks (P = 0.88) but they were seen at 6 months (P = 0.0097)	Fair
Chen et al. (2008)^60^	Intervention control trial	China	*Aim*: to report the effects of these two programmes on blood pressure and changes in morbidity and mortality from CHD and stroke *Outcomes*: salt intake	Health education	Mean daily salt intake declined from 16.0 to 10.6 g d-1 in the intervention factory, compared with the control factory from 16.9 to 15.4 g d-1, with the net reduction of 3.9 g d-1, which was significantly different (P < 0.05).	Fair
Levin et al. (2009)^61^	Worksite based dietary intervention	US	*Aim*: to examine whether a worksite nutrition programme using a low-fat vegan diet could significantly improve nutritional intake *Outcomes*: salt intake	Dietary counselling	Intervention group participants significantly increased the reported intake and mean intake (P = 0.04) of salt compared to the control group. *Salt (g/day)* Intervention group • Baseline: 4.1 ± 0.1 • 22 weeks: 5.0 ± 0.2 • Mean difference: 0.9 ± 0.2 Control group • Baseline: 4.5 ± 0.2 • 22 weeks: 4.9 ± 0.2 • Mean difference: 0.4 ± 0.2 Mean effect size: +0.5 (95% CI 9.2, 394.4; P = 0.04)	Fair

**Table 4:** Dietary counselling (community).

Study	Study type	Geographical scope	Aim and main outcomes	Policies analysed	Relevant results	Quality assessment
Yanek et al. (2001)^62^	RCT	US	*Aim*: to test the impact on cardiovascular risk profiles after one year of participation in one of three church-based nutrition and physical activity strategies *Outcomes*: salt intake	Health promotion–education	Salt (g/day) *Combined standard and spiritual intervention groups* • Baseline: 6.7 ±2.5 • Change: -0.4 ±0.06 *Self-help control group* • Baseline: 7.4 ±3.0 • Change: -0.02 ±0.09 Between group P value = 0.0167	Fair
Cappuccio et al. (2006)^63^	Community-based cluster randomised trial	Ghana	*Aim*: to establish the feasibility of salt reduction as a way of reducing BP *Outcomes*: salt intake	Health education	Sodium intake as measured by sodium excretion fell in four out of six villages in the intervention group and in 5 out of six villages in the control group. The net intervention effect was non-significant. *Control Intervention* Baseline: 6.0 g/day Baseline: 5.8 g/day 3 months: 5.6 g/day 3 months: 5.4 g/day 6 months: 5.2 g/day 6 months: 5.3 g/day	Fair
Takahashi et al. (2006)^64^	Community based open randomizer controlled cross-over trial	Japan	*Aim*: to assess whether dietary intervention in free-living healthy subjects is effective in improving blood pressure levels. *Outcomes*: salt intake as measured by urinary sodium excretion	Dietary education	Salt intake as measured by sodium excretion, collected at two points, in the intervention group decreased by 2.8 (95% CI: -3.6, -2.1) and 0.6 g/day (-1.4, +0.2) in the control group. This difference in change between the two groups was statistically significant (P < 0.001). Dietary counselling for 1 year reduced salt intake by 2.2 g/day as measured by 24-h urinary sodium	Fair
Robare et al. (2010)^65^	Community based intervention trial	US	*Aim*: to evaluate a dietary Na reduction trial in a community setting *Outcomes*: salt intake as measured by urinary sodium excretion	Nutrition education	Salt intake decreased by 0.3g/day (7.8 to 7.5g/day) from baseline to 6 months follow up which was not significant (p = 0.30). When comparing baseline with 12 months follow up, salt intake decreased by 0.7g/day (7.8 to 7.2g/day) which was significant (p = 0.03)	Fair
Van de Vijver et al. (2012)^66^	Review	Ghana and China	*Aim*: to evaluate the effectiveness of the community-based interventions for CVD prevention programmes in LMIC *Outcomes*: BP and salt intake (g/day and n, %)	Health education	**Cappuccio et al. (2006)** • *BP*: reduction SBP 2.5 mmHg (1.45 to 6.54), DBP 3.9 mmHg (0.78 7.11)* vs control • *Salt*: no significant reduction in salt intake vs control **Chen, Wu, and Gu (2008) (urban)** • *BP*: reduction SBP 1.9 mmHg, reduction DBP 2.2 mmHg* vs control • *Salt*: reduction in salt intake of 3.9 g/day* vs control **Yu et al. (1999)** • *BP*: reduction among men in prevalence in HT 2%,* SBP 0%, among women prevalence of HT 2%,* SBP 2 mmHg • *Salt*: reduction in salt intake 6.0% **Huang et al. (2011)** • *BP*: reduction prevalence HT 12.9%* pre vs post • *Salt*: reduction in salt intake 30%* (n, %)	Fair

**Table 5:** Media campaigns.

Study	Study type	Geographical scope	Aim and main outcomes	Policies analysed	Relevant results	Quality assessment
Shankar et al. (2012)^67^	Cross-sectional	UK	*Aim*: to examine the trend in salt intake over a set period and deduce the effects of the policy on the intake of socio-demographic groups *Outcomes*: salt intake as measured by spot urinary sodium readings	Salt campaign (and potential effect on reformulation and table salt use)	The results are consistent with a previous hypothesis that the campaign reduced salt intakes by approximately 10%. The impact is shown to be stronger among women than among men. Salt as measured by spot urinary sodium readings • 2003: 6.3 g/day • 2004: 6.4 g/day • 2005: 5.7 g/day • 2006: 5.6 g/day • 2007: 5.4 g/day Difference in g/day between 2003–2007 = 0.9 g/day = 13.5%	Fair

**Table 6:** Labelling.

Study	Study type	Geographical scope	Aim and main outcomes	Policies analysed	Relevant results	Quality assessment
Babio et al. (2013)^72^	Randomised cross-over trial	Spain	*Aim*: to compare two models of front-of-pack guideline daily amounts (GDA) and the ability to choose a diet that follows the nutritional recommendations. *Outcomes*: salt intake based on choices	Labelling	Participants using the multiple-traffic-light GDA system chose significantly less salt (0.4g/day; P <0.001) than those using the monochrome GDA labels	Poor
Elfassy et al. (2015)^73^	Cross-sectional	US	*Aim*: to examine independent association between hypertension and frequency use of NF label for sodium information and whether this was associated with differences in intake *Outcomes*: salt intake as measured by urinary sodium excretion	Labelling (use)	Daily sodium intake was not lower in those who reported frequent vs non-frequent use of the NF label for sodium information (7.7g/day vs 7.6g/day; P = 0.924)	Poor

**Table 7:** Reformulation.

Study	Study type	Geographical scope	Aim and main outcomes	Policies analysed	Relevant results	Quality assessment
Chang et al. (2006)^78^	Cluster RCT	Taiwan	*Aim*: to examine the effects of potassium-enriched salt on CVD mortality and medical expenditures in elderly veterans. *Outcomes*: incidence, CVD mortality, LYG	Reformulation–low sodium salt	The incidence of CVD-related deaths was 13.1 per 1000 persons (27 deaths in 2057 person-years) and 20.5 per 1000 (66 deaths in 3218 person years) for the experimental and control groups, respectively A significant reduction in CVD mortality (age-adjusted hazard ratio: 0.59; 95% CI: 0.37, 0.95) was observed in the experimental group. Persons in the experimental group lived 0.3–0.90 y longer	Fair

**Table 8:** Taxes.

Study	Study type	Geographical scope	Aim and main outcomes	Policies analysed	Relevant results	Quality assessment
Thow et al. (2014)^85^	Systematic Review	US (with UK data)	*Aim*: to assess the effect of food taxes on consumption *Outcomes*: sodium consumption	Sodium tax	A modelling study predicted that a sodium tax increasing the price of salty foods by 40% would reduce sodium consumption by 6%	Fair
Niebylski et al. (2015)^86^	SystematicReview	France and US	*Aim*: to evaluate the evidence base to assess the effect of unhealthy food taxation. *Outcomes*: energy intake	1) Tax on salty snacks 2) Tax on cheese/butter	1) Modelling study of tax on chips/salty snacks on energy intake in US. Predicted a 1% tax had no effect on consumption or body weight 2) Modelling study of effect of 1% VAT on cheese/butter, sugar, and fat products along with ready-made meals in France. Predicted proposed taxes reduced saturated fat, cholesterol, sodium, and energy intake but suggest 1% is insufficient to have positive health effect.	Fair

**Table 9:** Multi-component interventions.

Study	Study type	Geographical scope	Aim and main outcomes	Policies analysed	Relevant results	Quality assessment
He et al. (2014)^88^	Comprehensive analysis	UK	*Aim*: to analyse the UK salt reduction programme *Outcomes*: salt intake as measured by urinary sodium excretion	1) Reformulation 2) Labelling 3) Health promotion campaigns	15% decrease, there have been a steady fall in salt intake at a rate of ~2% per year since the introduction of the salt reduction strategy. The 0.9g/day reduction in salt intake achieved by 2008 led to E 6000 fewer CVD deaths per year. • 2000–2001: salt intake = 9.5g/day • 2005–2006: salt intake = 9.0g/day • 2008: salt intake = 8.6g/day • 2011: salt intake = 8.1g/day	Good
Mozaffarian et al. (2012)^89^	Systematic review	Finland and China	*Aim*: to systematically review and grade the current scientific evidence for effective population approaches to improve dietary habits. *Outcomes*: salt intake as measured by urinary sodium excretion	1) Education 2) Combined effects of labelling, reformulation and campaigns	**Tian et al. (1995)** *1) Education*: In the intervention neighborhoods, mean sodium intake decreased by 1.3 and 0.6 mmol/day in men and women, respectively, compared with increases of 1.0 and 0.2 mmol/day, respectively, in the control neighborhoods (P0.001 for men, P0.065 for women) **Pekka et al. (2002) + Puska & Stahl (2010)** 2) From the 1970s to the late 1990s, mean daily salt consumption in Finland declined from approximately 14.5 g in men (unknown in women) to approximately 11 g in men and 7 g in women; mean diastolic blood pressure declined by 5% in men and 13% in women	Good
Fattore et al. (2014)^43^	Systematic review	Australia, US and Vietname	*Aim*: to summarize and critically assess economic evaluation studies conducted on direct (e.g., counseling) or indirect (e.g., food labeling) interventions aimed at promoting voluntary dietary improvements through reduction of fat intake *Outcomes*: DALYs	1) Voluntary reformulation, mandatory reformulation and dietary advice 2) Reduction in daily caloric intake of 100 to 500 kcal below current estimated energy requirements 3) A set of personal (e.g., individual treatment of SBP >160 mmHg) and non-personal (e.g., a mass media campaign for reducing consumption of salt) prevention strategies to reduce CVD 4) Voluntary reformulation and sodium tax	1) **Cobiac et al. (2010)** 610,000 DALYs averted (95%CI: 480,000–740,000) if everyone reduced their salt intake to recommended limits. Dietary advice: <0.5% disease burden (IHD & stroke cases) averted; Tick program: <1%; making Tick limits mandatory: 18% 2) **Dall et al. (2009)** 400 mg/d sodium intake reduction 3) **Ha & Chisholm (2011)** A health education program to reduce salt intake (VND 1,945,002 or USD 118 per DALY averted) & individual treatment of SBP >160 mmHg (VND 1,281,596 or USD 78 per DALY averted) are the most cost-effective measures 4) **Smith-Spangler (2010)** (1) vs. (2): 1.25-mm Hg vs. 0.93-mm Hg decrease in mean SBP; 513,885 vs. 327,892 strokes averted; 480,358 vs. 306,137 MIs averted; 1.3 million vs. 840,113 years LE increase. Collaboration with industry: 2.1 million QALYs gained; USD 32.1 billion medical cost savings. Tax on sodium: 1.3 million QALYs gained; USD 22.4 billion medical cost savings	Fair
He & MacGregor (2009)^90^	Review	Japan, Finland and UK	*Aim*: to provide an update on the current experience of worldwide salt reduction programmes. *Outcomes*: salt intake, blood pressure, stroke & CHD mortality and life expectancy	1) Reformulation to reduce the salt content of all foods 2) Health promotion campaigns 3) Labelling to highlight salt content	*Japan*. The Japanese Government initiated a campaign to reduce salt intake. Over the following decade salt intake was reduced from an average of 13.5 to 12.1 g/day. However, in the north of Japan salt intake fell from 18 to 14 g/day. Paralleling this reduction in salt intake, there was an 80% reduction in stroke mortality despite large increases in population fat intake, cigarette smoking, alcohol consumption and an increase in BMI. *Finland*. Since the 1970s, Finland aimed to reduce salt intake by reformulation and raising general awareness of the harmful effects of salt on health. This led to a significant reduction in salt intake of 3g/day from 1979 to 2002 (12 to 9g/day) as measured by urinary sodium. This was accompanied by a fall of over 10mmHg in both systolic and diastolic BP, a pronounced decrease of 75–80% in both stroke and CHD mortality, and a remarkable increase of 5–6 years in life expectancy. *UK*. **Salt added to cooking or at the table:** estimated that 15% of the total 9.5g/day consumed was added (1.4g/day). **Naturally present in food:** approximately 5% (0.6g/day). **Reformulation:** 80% (7.5g/day) was added by the food industry. The UK salt reduction strategy started in 2003/2004 and the adult daily salt intake has already fallen, as measured by urinary sodium, from an average of 9.5 g/day to 8.6 g/day by May 2008	Fair
Pietinen et al. (2010)^91^	Before and after study	Finland	*Aim*: to describe the main actions in Finnish nutrition policy during the past decades. *Outcomes*: salt intake	1) Education 2) Voluntary reformulation 3) Labelling	1981; Eastern Finland: salt intake was about 13 g in men and 11 g in women. Salt intake has decreased continuously to a level of about 9 g in men and 7 g in women in 2007	Fair
Wang et al. (2011)^92^	Literature review	US	*Aim*: to summarize cost-effectiveness evidence on selected interventions to reduce sodium intake that would be intended as population-wide approaches to control hypertension *Outcomes*: stroke and MI averted	1) Reformulation 2) Sodium tax	**Smith-Spangler et al.** For US adults aged 40–85 years, collaboration with industry that decreased mean intake of sodium by 9.5% was estimated to avert 513 885 strokes and 480 358 myocardial infarctions over their lifetimes and to save US$ 32.1 billion in annual medical costs. Over the same period, a tax on sodium that decreased the population’s intake of sodium by 6% was projected to save US$ 22.4 billion in such costs	Fair
Webster et al. (2011)^93^	Review	Finland, France, Japan and UK	*Aim*: to provide an overview of national salt reduction initiatives around the world and describe core characteristic. *Outcomes*: salt intake, LYG, CHD and stroke mortality	1) Reformulation 2) Labelling 3) Health promotion campaigns	*Finland*: started salt reduction strategy in 1978 (reformulation, labelling and mass media campaigns) and by 2002 had demonstrated a 3 g reduction in average population salt intake (from 12 to 9 g/person per day). During the same period there was a corresponding 60% fall in CHD and stroke mortality *UK*: the Food Standards Agency (FSA) started working with the food industry in 2003 and launched its consumer education campaign in 2005. By 2008 the UK had achieved an average 0.9 g/person per day reduction in daily salt consumption, which is predicted to be saving some 6000 lives a year. *France*: the Food Safety Authority recommended a reduction in population salt consumption in 2000 and has since reported a decline in intake provided by foods from 8.1 to 7.7 g/day in the overall adult population. **Focus was on bread reformulation and nutrition campaigns** *Japan*: 60s started a salt campaign through a sustained public education campaign. Over the following decade average salt intake was reduced from 13.5 to 12.1 g/day with a parallel fall in blood pressure in adults and children, and an 80% reduction in stroke mortality despite large adverse changes in a range of other cardiovascular risk factors.	Fair
Wang & Bowman (2013)^94^	Literature review	US, UK	*Aim*: to summarize recent economic analyses of interventions to reduce sodium intake. *Outcomes*: SBP, hypertension, cardiovascular events	1) reducing the sodium content of all foods 2) reducing sodium content by labelling foods and by promoting, subsidizing, and providing low sodium food options 3) Legislation	*US (1&2)*: If the sodium-reduction strategies were implemented, adults in the county would reduce their intake of sodium by 233 mg per day, on average, in 2010. This would correspond to an average decrease of 0.71 mmHg in SBP among adults with hypertension, 388 fewer cases of uncontrolled hypertension, and a decrease per year of $629,724 in direct health care costs *UK (3)*: Legislation or other measures to reduce the intake of salt by 3 g per person per day (in a population where the current mean intake was about 8.5 g per person per day) would reduce the mean population SBP by approximately 2.5 mmHg, prevent about 30,000 cardiovascular events and approximately 4,450 deaths, and produce discounted savings overall of approximately £347 million (about $684 million) over a decade, which would be equivalent to annual savings of approximately £40 million	Fair
He et al. (2014)^95^	Cross-sectional	England	*Aim*: to determine the relationship between the reduction in salt intake that occurred in England, and BP, as well as mortality from stroke and IHD *Outcomes*: salt intake as measured by urinary sodium excretion	Combined 1) Reformulation 2) Health promotion campaigns 3) Labelling	From 2003 to 2011, salt intake decreased by 1.4 g/day (15%, p<0.05 for the downward trend). From 2003 to 2011, stroke mortality decreased from 128/1 000 000 to 82/1 000 000 (36% reduction, p<0.001) and IHD mortality decreased from 423/1 000 000 to 272/1 000 000 (36% reduction, p<0.001). • 2003: 9.5g/day • 2005/2006: 9.0g/day • 2008: 8.6g/day • 2011: 8.1g/day	Fair
Enkhtungalag et al. (2015)^96^	Before and after study	Mongolia	*Aim*: to reduce salt intake of the employees of three of the main food producing factories. *Outcomes*: salt intake as measured by 24h urine excretion	Education on salt consumption and provision of reduced salt foods	Salt intake reduced from 11.5g/day in 2011 to 8.7g/day in 2013	Fair
Trieu et al. (2015)^24^	Systematic review	75 countries	*Aim*: to quantify progress with the initiation of salt reduction strategies around the world in the context of the global target to reduce population salt intake by 30% by 2025. *Outcomes*: *salt (g/day)*	Labelling, mass media campaigns, education, reformulation	*Denmark*: from 2006 to 2010 salt intake reduced from 10.7 to 9.9g/day in men and 7.5g to 7.0g/day in women (7%) *Japan*: salt intake reduced from 13.5in 1997 to 10.4g/day in 2012 (23%) *Korea*: salt intake reduced from 13.4g in 2005 to 11.6g/day in 2012 (13.6%) *Slovenia*: salt intake reduced from 12.4g in 2007 to 11.3g/day in 2012 (8.9%) **Du et al. (2014)** *China*: salt intake reduced from 16.8g in 1999 to 12g/day in 2009 (28%) **Pietinen et al. (2010) & Laatikanen et al. (2006)** *Finland*: from 1979 to 2007 salt intake reduced from 13g to 8.3g/day in men and 11g to 7.0g/day in women (36%) **European commission (2008)** *France*: salt intake reduced from 8.1g in 1999 to 7.7g/day in 2007(4.9%) **WHO (2013)** *Iceland*: salt intake reduced from 8.4g in 2002 to 7.9g/day in 2010 (6%) **Walton (2013)** *Ireland*: salt intake reduced from 8.1g in 2001 to 7g/day in 2011(13.6%) **National Food and Veterinary Risk Assessment Institute** *Lithuania*: salt intake reduced from 10.8g in 1997 to 8.8g/day in 2007(18.6%) **WHO (2013)** *Turkey*: salt intake reduced from 18.0g in 2008 to 15g/day in 2012(16.7%) **Sadler et al. (2011)** *UK*: Salt intake reduced from 9.5g in 2001 to 8.1g/day in 2011(14.7%)	Fair
Luft et al. (1997)^97^	Review	Finland and US	*Aim*: to discuss the approaches used in a community-wide salt-reduction project. *Outcomes*: salt intake as measured by urinary excretion	1) Nutrition education 2) Reformulation	**Pietinen et al. (1984)—***Health education & reformulation*. After 3 y salt intake had not changed significantly. Hypertensive subjects **Men** **Women** 1979: 13.8 ± 5.3 1979: 10.4 ± 4.7 1982: 13.7 ± 5.5 1982: 10.0 ± 4.1 Normotensive subjects **Men** **Women** 1979: 12.4 ± 4.8 1979: 9.8 ± 3.8 1982: 12.2 ± 4.8 1982: 9.1 ± 3.6 **Lang et al. (1985)—***Dietary counselling*. Women reduced their salt intake from 7.5 ± 0.4 to 3.6 ± 0.2 g/day and men reduced their salt intake from 10.3 ± 0.8 to 4.7 ± 0.3 g/day. **Wassertheil-Smoller et a. (1992)–***Education*. Salt intake as measured by urinary sodium excretion was reduced from 7.9 to 1 6.4 g/day. Analysis of 3-d food records indicated that sodium intake decreased from 8.1 to 4.9 g/day. **Hypertension prevention collaborative research group (1992)—***Nutrition education*. Salt intake as measured by urinary sodium excretion *Intervention Control* Baseline: 8.9 ± 3.4 Baseline: 9.0 ± 3.5 Change: -3.2 ± 4.4 Change: -0.6 ± 4.4	Poor
Mohan et al. (2009)^98^	Review	UK	*Aim*: to review the evidence related to dietary sodium and health in the context of the Ottawa Charter for health promotion. *Outcomes*: salt intake, stroke, CVD & coronary artery mortality	1) Reformulation 2) Labelling 3) Health promotion campaign	*UK*: Consumer-friendly labelling indicating sodium content in processed foods by use of a colour system implemented in several UK food chains. Together with other efforts population salt intake decreased from 9.5g/day in 2004 to 8.6g/day in 2008	Poor
He & MacGregor et al. (2010)^99^	Comprehensive review	Japan, Finland and UK	*Aim*: to provide an update on the current salt reduction programmes that have been successfully carried out *Outcomes*: salt intake	1) Reformulation 2) Labelling 3) Health promotion campaigns	*Japan*: over a decade national salt intake fell from 13.5g/day to 12.1g/day. In the North, salt intake was reduced from 18 to 14g/day. There was also an 80% reduction in stroke mortality despite large increases in fat intake, cigarette smoking, alcohol consumption, and obesity *Finland*: reformulation, labelling and campaigns led to a significant reduction in salt from 12g/day in 1979 to 9g/day in 2002 *UK*: salt reduction strategy started in 2003/2004 and salt intake has already fallen from 9.5 to 8.6 g/d by May 2008	Poor
Wyness et al. (2012)^100^	Literature review	UK	*Aim*: to describe the UK Food Standards Agency's (FSA) salt reduction programme undertaken between 2003 and 2010 and to discuss its effectiveness *Outcomes*: salt intake	1) Health promotion campaigns 2) Voluntary reformulation 3) Labelling	• 2000–2001: salt intake = 9.5g/day • 2005–2006: salt intake = 9.0g/day • 2008: : salt intake = 8.6g/day	Poor

**Table 10:** Salt intake outcomes with interventions detailed in other publications.

Study	Study type	Geographical scope	Aim and main outcomes	Policies analysed	Relevant results	Quality assessment
Laatikainen et al. (2006)^25^	Cross-sectional population surveys	Finland	*Aim*: to present trends in urinary sodium and potassium excretion from 1979 to 2002 *Outcomes*: salt intake as measured by urinary sodium excretion	1) Reformulation 2) Mass media campaigns 3) Labelling	Between 1979 and 2002 salt intake as measured by sodium excretion decreased from over 12.7g/day to less than 9.8g/day among men and from nearly 10.4 to less than 7.5g/day among women. In 1979 the most educated North Karelian men had lower salt intake compared to the least educated being 11.4 g in the highest education tertile and 13.1 g in the lowest tertile. Respectively, in 2002, the salt intake in southwestern Finland among women in the highest education tertile was 6.7g compared to 8.1g in the lowest tertile	Good
Otsuka et al. (2011)^101^	Longitudinal study	Japan	*Aim*: to describe salt intake over 8 years according to age groups. Also to examine whether salt intake changes over time in middle-aged and elderly Japanese subjects *Outcomes*: salt intake		In stratified analyses by age, mean salt intake in men decreased 0.08 g/year among 40- to 49-year-olds, 0.09 g/year among 50- to 59-year-olds, 0.16 g/year among 60- to 69-year-olds, and 0.14 g/year among 70- to 79-year-olds. For women, mean salt intake decreased 0.08 g/year among 70- to 79-year-olds (P0.098).	Fair
Du et al. (2014)^102^	Ongoing open cohort study	China	*Aim*: to analyse the patterns and trends of dietary sodium intake, potassium intake and the Na/K ratio and their relations with incident hypertension. *Outcomes*: salt intake as measured per 24h dietary recalls	Labelling & media campaign	Salt intake decreased from 16.5g/day in 1991 to 11.8g/day in 2009	Fair
Miura et al. (2000)^103^	Report	Japan	*Aim*: to present the status of salt consumption, salt-reducing measures/guidance methods in individual and population strategies to reduce salt intake *Outcomes*: salt intake		The National Health and Nutrition Survey in 2010 reported that the mean salt intake in adults was 10.6 g/day. There was an ~4 g decrease in comparison with that in 1972 (14.5 g), when salt intake was investigated for the first time in the National Nutrition Survey	Poor

**Table 11:** Modelling studies included in the systematic review.

	Study	Study type	Geographical scope	Aim and main outcomes	Policies analysed	Relevant results	Quality assessment
**Salt**	Cobiac et al. (2010)^54^	Modelling study	Australia	*Aim*: to evaluate population health benefits and cost-effectiveness of interventions for reducing salt in the diet. *Outcomes*: DALYs and proportion of DALYs averted	1) Voluntary reformulation 2) Mandatory reformulation 3) Dietary advice	*Mandatory reformulation*: could avert 18% of the disease burden (110,000 DALYs). *Dietary advice*: might avert less than 0.5% of the disease burden (1,700–2,600 DALYs) *Voluntary Reformulation*: modelled for breads, margarines and cereals would avert less than 1% of the disease burden(5,300 DALYs) Voluntary reformulation and mandatory salt reduction had a 100% probability of being dominant (i.e., cost saving to the health sector) under all modelled scenarios. Dietary advice had zero probability of being cost-effective.	Good
	Cobiac et al. (2012)^55^	Modelling study	Australia	*Aim*: to evaluate the optimal mix of lifestyle, pharmaceutical and population-wide interventions for primary prevention of cardiovascular disease *Outcomes*: DALYs	1) Mandatory reformulation 2) Community heart health programme 3) Dietary advice	*Mandatory reformulation* in breads, margarines and cereals is easily the most effective and cost-effective strategy for primary prevention of CVD; (80,000 DALYs) and cost saving (dominant). *Community heart health program* (3,000 DALYs; $44,000) *Dietary advice* (180–370 DALYs) are least cost-effective of all the primary prevention strategies ($ 1,000,000 to $1,400,000)	Good
	Nghiem et al. (2015)^56^	Modelling study	New Zealand	*Aim*: to compare the impact of eight sodium reduction interventions *Outcomes*: QALYs	1) Dietary counselling 2) Labelling 3) Mandatory 3G reformulation (breads, processed meats and sauces) 4) Mandatory ‘All’ reformulation 5) UK package (multiple policies) 6) Mass media campaign 7) Tax	QALYs gained in order of effectiveness: 1) Salt tax (195,000) 2) Mandatory ‘all’ reformulation (110,000) 3) UK package (85,100) 4) Mandatory 3G reformulation (61,700) 5) Mass media campaign as per the UK one (25,200) 6) Voluntary labelling (7,900) 7) Dietary counselling (200)	Good
	Collins et al. (2014)^68^	Modelling study	UK	*Aim*: to evaluate the cost-effectiveness of four population health policies to reduce dietary salt intake on an English population to prevent coronary heart disease (CHD). *Outcomes*: life years gained and salt intake	1) Health promotion campaign 2) Labelling 3) Voluntary salt reformulation 4) Mandatory salt reformulation	*Primary outcomes*: Salt intake reductions: Campaign = 0.16g/d; Labelling = 0.16g/d; Voluntary reformulation = 1.21g/d; Mandatory reformulation = 1.62g/d *Secondary outcomes*: Gains: Change4life and labelling might each gain approximately = 1960 life-years; Voluntary reformulation = 14,560 life-years; and Mandatory reformulation 19,320 life-years.	Good
	Gillespie et al. (2015)^69^	Modelling study	England	*Aim*: to forecast the potential impact on English adults of policies implemented during the 2015 UK parliament, projecting the health consequences to 2025 *Outcomes*: salt intake, CHD deaths prevented, LYG	1) Mandatory reformulation 2) Voluntary reformulation 3) Social marketing 4) Nutrition labelling	Mandatory reformulation (30% reduction in salt content) • Salt intake = -1.45g/day • SBP = -0.81mmHg • CHD deaths = 4.500 prevented or postponed • LYG = 44.000 Mandatory reformulation (10% reduction in salt content) • Salt intake = -0.48g/day • SBP = -0.27mmHg • CHD deaths = 1.500 prevented or postponed • LYG = 15.000 Voluntary reformulation • Salt intake = -0.48g/day • SBP = -0.27mmHg • CHD deaths = 1.500 prevented or postponed • LYG = 14.000 Social marketing (50% impact) • Salt intake = -0.13g/day • SBP = -0.078mmHg • CHD deaths = 400–500 prevented or postponed • LYG = 5.000 Social marketing (10% impact) • Salt intake = -0.027g/day • SBP = -0.015mmHg • CHD deaths = 100 prevented or postponed • LYG = 780 Nutrition labelling (50% impact) • Salt intake = -0.16g/day • SBP = -0.091mmHg • CHD deaths = 500 prevented or postponed • LYG = 5.000 Nutrition labelling (10% impact) • Salt intake = -0.031g/day • SBP = -0.018mmHg • CHD deaths = 100 prevented or postponed • LYG = 1.000	Good
	Wilcox et al. (2014)^70^	Modelling study	Syria	*Aim*: to present a cost-effectiveness analysis of salt reduction policies to lower coronary heart disease in Syria. *Outcomes*: salt intake, deaths prevented and life years gained	1) Health promotion 2) Labelling 3) Reformulation	**Health promotion campaign**– *5% reduction in salt intake*. 252 deaths prevented; 5.679 LYG **Labelling**– *10% reduction in salt intake*. 497 deaths prevented; 11.192 LYG **Reformulation**– *10% reduction in salt intake*. 497 deaths prevented; 11.192 LYG **Reformulation + HP** & **Reformulation + Labelling**– *15% reduction in salt intake*. 735 deaths prevented; 16.543 LYG **All 3 policies**– *30% reduction in salt intake*. 1.413 deaths prevented; 31.674 LYG	Good
	Mason et al. (2014)^71^	Modelling study	Tunisia, Syria, Palestine and Turkey	*Aim*: to present an economic evaluation of population based salt reduction policies in Tunisia, Syria, Palestine and Turkey. *Outcomes*: life years gained	1) Health promotion campaign (HP) 2) Labelling (L) 3) Mandatory reformulation (R)	*Tunisia*: HP = 1.151 LYG; L = 2.272 LYG; R = 2.272 LYG; All 3 policies = 6.455 LYG *Syria*: HP = 5.679 LYG; L = 11.192 LYG; R = 11.192 LYG; All 3 policies = 31.674 LYG *Palestine*: HP = 479 LYG; L = 945 LYG; R = 945 LYG; All 3 policies = 2.682 LYG *Turkey*: HP = 68.816 LYG; L = 135.221 LYG; R = 135.221 LYG; All 3 policies = 199.303 LYG	Fair
	Pietinen et al. (2008)^74^	Modelling study	Finland	*Aim*: to estimate the impact of choosing food products labelled either as low or high in salt *Outcomes*: salt intake	Salt labelling	If the entire population were to choose low-salt breads, cheeses, processed meat and fish, fat spreads, and breakfast cereals, then salt intake could be lowered by 1.5 g in men and by 0.9 g in women. If everybody was to select high-salt products, then salt intake would go up by 1.9 g in men and by 1.2 g in women. Thus, the potential difference between the low and the high alternatives would be 3.4 g in men and 2.9 g in women. If all prepared foods had a reduced salt content, the mean salt intake would go further down by 2.3 g in men and by 1.7 g in women. Excluding under reporters, the mean salt intake was 11.1 g in men and would go down to 9.5 g if all men chose lightly salted products and further down to 6.8 g if also all prepared foods would have a lower salt content. In women, the respective numbers are 7.8, 6.7 and 4.9 g	Fair
	Temme et al. (2010)^75^	Modelling study	Netherlands	*Aim*: to evaluate the effects of changed food compositions according to health logo criteria on the intake of saturated fat, sugar and sodium *Outcomes*: salt intake	Labelling (health logos)	At baseline salt intake was 7.3 (95% CI 2.8, 2.9) g/day. For salt, in a 100% market share scenario (scenario II), salt reduction expected is 0.3g/day (4% reduction; non-significant). In scenario III, when all non-complying foods are replaced with foods complying with health logo criteria, sodium intake reduced by 23% to 5.5g/d (-1.8g/day).	Fair
	De Menezes et al. (2013)^76^	Modelling study	Brazil	*Aim*: to evaluate the impact of introducing products that are in agreement with the Choices criteria in the usual diet. *Outcomes*: salt intake	Food Labelling	Salt would still be considered an important reduction, 36% in relation to the typical menus (TM), but it would be 7.6 g/day, which is above the recommended by the program (6.5 g/day). **Salt (g/day)** • *Typical menus (TM):* 11.9 ± 1.2 • *Choices menus (CM):* 6.3 ± 0.1 • *Choices menus energy (CME)–same as CM, but adjusted for energy of TM:* 7.6 ± 0.9	Fair
	Roodenburg et al. (2013)^77^	Modelling study	The Netherlands	*Aim*: to evaluate these nutritional criteria by investigating the potential effect on nutrient intakes *Outcomes*: salt intake	Labelling	A reduction of -23% for sodium was seen for sodium compared to the ‘actual scenario’. **Salt (g/day)** • *Actual*: 7.4 • *Choices*: 5.7 • *Choices energy adjusted*: 6.5 • *Snacks (partially replaced)*: 5.8 • *Snacks (not replaced)*: 5.9	Fair
	Choi et al. (2015)^41^	Modelling study	US	*Aim*: to estimate the cardio-vascular impact of the expanded NSRI among different segments of the US population and under varying possible producer and consumer responses to the initiative *Outcomes*: sodium intake, MIs, stroke and hypertension	Reformulation (restaurants and manufacturers)	*Restaurants and manufacturers reaching agreed upon sodium targets*. Expansion of the initiative to ensure all restaurants and manufacturers reach agreed-upon sodium targets would be expected to avert from 0.9 to 3.0 MIs (a 1.6%–5.4% reduction) and 0.5 to 2.8 strokes (a 1.1%–6.2% reduction) per 10,000 Americans per year over the next decade, after incorporating consumption patterns and variations in the effect of sodium reduction on blood pressure among different demographic groups. The expanded NSRI covering both packaged and restaurant food items would be expected to reduce mean daily sodium intake by 447 mg per person per day on average, or 13.0%. If the NSRI included only restaurant food items, the program would lower MI and stroke mortality by an estimated 2.7% (95% CI, 1.4–4.0) and 2.1% (95% CI, 0.4–3.9), respectively. Hence, most of the benefit from the program would likely be due to sodium changes among packaged foods	Good
	Murray et al. (2003)^79^	Modelling study	South East Asia (SEA), Latin America (LA), Europe (EU)	*Aim*: to report estimates of the population health effects, and costs of selected interventions to reduce the risks associated with high cholesterol and blood pressure in areas of the world with differing epidemiological profiles *Outcomes*: DALYs	1) Voluntary reformulation 2) Mandatory reformulation	Measures to decrease salt intake appeared cost effective. Legislation appeared more cost effective than voluntary agreements with assumption it would lead to a larger reduction in dietary salt intake. A 15% reduction in mean population salt intake could avert 8.5 million cardiovascular deaths Voluntary reformulation: • *EU*: 7 X10^6^ DALYs averted ($44 per DALY) • SEA: 5X10^6^ DALYs averted ($37 per DALY) • *LA*: 3 X10^6^ DALYs averted ($24 per DALY) Mandatory reformulation • *EU*: 13x10^6^ DALYs averted ($23 per DALY) • *SEA*: 10 x10^6^ DALYs averted ($19 per DALY) • *LA*: 6 x10^6^ DALYs averted ($13 per DALY)	Good
	Rubinstein et al. (2010)^80^	Modelling study	Argentina	*Aim*: to estimate the burden of acute CHD and stroke and the cost-effectiveness of preventative population-based and clinical interventions. *Outcomes*: DALYs	Salt reduction in bread	*Reducing salt in bread is cost-saving* • DALY averted: 672.80 • % of DALY saved: 0.11% • International Dollars per DALY saved: 1,406.93	Good
	Smith-Spangler et al. (2010)^81^	Modelling study	US	*Aim*: to assess the cost-effectiveness of two population strategies to reduce sodium intake *Outcomes*: strokes and MIs averted, life years and QALYs gained	1) Voluntary reformulation 2) Sodium tax	*Collaboration with the industry*: a 9.5% reduction in sodium intake resulted in • Averted strokes = 513 885 s • Averted MIs = 480 358 • LYG = 1.3 million • QALYs = 2 million *Sodium tax*: would lead to a 6% decrease in sodium intake. • Averted Strokes = 327 892 • Averted MIs = 306 137 • LYG = 840 113 • QALYs = 1.3 million	Good
	Konfino et al. (2013)^82^	Modelling study	Argentina	*Aim*: to use Argentina-specific data to project impact of Argentina’s sodium reduction policies under two scenarios—the 2-year intervention currently being undertaken or a more persistent 10 year sodium reduction strategy. *Outcomes*: salt intake as measured by urinary sodium excretion, systolic blood pressure, deaths and cases averted, mortality	Reformulation	*Scenario 1: current initiative (2 year intervention)* • Projected to reduce mean salt consumption by 0.96 g/day in men and 0.79 g/day in women • SBP would reduce by 0.93 mmHg to 1.81 mmHg depending on population subgroup • 19.000 deaths, 6.000 CHD deaths and 2.000 stroke deaths, 13.000 MIs and 10.000 stroke cases averted • Overall mortality reduction of 0.6% in adults >35 years, 1.5% in total MIs, 1% in total stroke cases in the next decade *Scenario 2: current initiative maintained for 10 years* • Projected to reduce mean salt consumption by 4.83 g/day in men and 3.98 g/day in women • SBP would reduce by 4.66 mmHg to 9.04 mmHg depending on subgroup • 55.000 deaths, 16.000 CHD deaths and 5.000 stroke deaths, 38.000 MIs and 27.000 strokes averted • Overall mortality decreased by 2% in adults > 35 years, 4.3% MIs and 2.7% stroke cases in the next decade	Good
	Rubinstein et al. (2009)^83^	Modelling study	Argentina	*Aim*: to use generalised cost-effectiveness analysis to identify the most efficient interventions to decrease CVD. *Outcomes*: cost-effectiveness; DALYs	Reformulation in bread	Lowering salt intake in the population through reducing salt in bread was found to be the most cost-effective ($17 per DALY averted). Less salt in bread • Total Cost per year (ARS$): $ 9.644 • DALY Age weighted, 3% discounted per year: 579 • DALY No age-weight 3% discounted per year: 713 • DALY # age-weight, undiscounted per year: 1,107 • ARS$ (1)/DALY (2): $ 17	Fair
	Hendriksen et al. (2014)^84^	Modelling study	Netherlands	*Aim*: to evaluate the health benefits of salt-reduction strategies related to processed foods. *Outcomes*: AMI, CHF and CVA averted, life expectancy and DALYs gained, salt intake (g/day)	1) Reformulation 2) Substitution of high salt foods with low salt foods 3)Adherence to the recommended intake	If salt intake is reduced to the *recommended maximum salt intake (6 g/d)*: Prevented cases: • 31.800 cases of AMI; • 15.300 cases of CHF; • 51.900 cases of CVA. • Mortality reduction: 0.7%. • LE increased by 0.15 years • 56000 DALYs gained *Salt reduction processed foods scenario*: median salt intake would decrease by -2.3g/d (28%) Prevented Cases: • 29.200 AMI cases; • 16.600 CHF cases; • 53.400 CVA. • Mortality Reduction: 0.8% • LE increased by 0.15 years • 56.400 DALYs gained *Substitution*: median salt intake would decrease by - 3.0g/day (35%). Prevented Cases: • 35.5000 cases of AMI; • 20.000 cases of CHF; • 64.300 cases of CVA • LE increased by 0.18 years • 67.900 DALYs gained	Fair
	Ni Mhurchu et al. (2015)^87^	Modelling study	New Zealand	*Aim*: to estimate the effects of health-related food taxes and subsidies *Outcomes*: deaths prevented or postponed	Tax on major dietary sodium products	A 20% tax on major dietary sources of sodium might result in 2,000 (1300 to 2,700) DPP (6.8%)	Good
	Asaria et al. (2007)^104^	Modelling study	23 low and middle income countries	*Aim*: to investigate potential deaths averted over 10 years by implementation of selected population-based interventions. *Outcomes*: CVD deaths averted, salt reduction (g/day)	Combined: 1) Mass media campaign 2) Voluntary reformulation	8.5 million deaths would be averted by implementation of the salt-reduction strategy (15%) alone. Salt interventions: **15% reduction in mean salt intake** • risk factor reduction of 1.69g/day • 8.4 million CVD deaths averted **30% reduction in mean intake** • risk factor reduction of 3.38g/day • 16.0 million CVD deaths averted **Reducing salt intake to 5g/d** • risk factor reduction of 6.28g/d ay • 28.3 million CVD deaths averted **Reduction in deaths** • CVD = 75.6% Respiratory disease = 15·4% Cancer = 8·7%	Good
	Dodhia et al. (2012)^105^	Modelling study	England	*Aim*: to assess the impact of cost-effective interventions in terms of the avoidable CVD burden and costs by comparing these strategies to the current situation *Outcomes*: IHD and stroke events and deaths avoided, DALYs	Combined 1) Health promotion 2) Reformulation	30% reformulation through agreement with the food industry. Interventions: **Na– 2mmHg** • IHD events avoided: 56.116 • Stroke events avoided: 98.497 • IHD deaths avoided: 26.781 • Stroke deaths avoided: 39.557 • DALYs averted: 238.043 • Cost per DALY ($): -4.228 **Na– 5mmHg** • IHD events avoided: 120.138 • Stroke events avoided: 257.508 • IHD deaths avoided: 57.322 • Stroke deaths avoided: 103.492 • DALYs averted: 579.869 • Cost per DALY ($): -5.021 Reducing salt intake to 6g/day through reformulation **NA–MRC review** • IHD events avoided: 80.366 • Stroke events avoided: 128.032 • IHD deaths avoided: 38.372 • Stroke deaths avoided: 51.419 Reducing salt intake in the population with a 5 mmHg reduction in SBP had the greatest population impact and cost-saving to the NHS.	Good
	Gase et al. (2011)^106^	Modelling study	US	*Aim*: to examine approaches to reduce sodium content of food served in settings operated or funded by the government of the County of Los Angeles, California *Outcomes*: salt intake and BP	Combined: 1) Labelling 2) Promotion 3) Subsidy 4) Provide low sodium food options	**Hospital cafeterias:** Average sodium reduction of 1.8g/day (23%). Overall SBP: 1.59 **County government cafeterias:** Average sodium reduction of 0.7g/day (11%). Overall SBP: 0.63	Fair
	Ha & Chrisholm (2011)^107^	Modelling study	Vietnam	*Aim*: to assess costs, health effects and cost-effectiveness prevention strategies to reduce CVD *Outcomes*: DALYs	Combined 1) Mass media campaign 2) Voluntary reformulation	*Media salt campaign* • Cost per year (USD, million): 4.1 • DALYs averted per year: 45.939 • VND per DALY saved: 89.2	Fair
	Barton et al. (2011) ^120^	Modelling study	England and Wales	*Aim*: to estimate the potential cost effectiveness of a population-wide risk factor reduction programme aimed at preventing cardiovascular disease. *Outcomes*: BP, CVD deaths averted	Salt legislation	Reducing salt intake by 3 g/day might reduce mean population systolic blood pressure by approximately 2.5 mm Hg preventing approximately 4450 deaths from cardiovascular disease	Good

**Fig 1:**
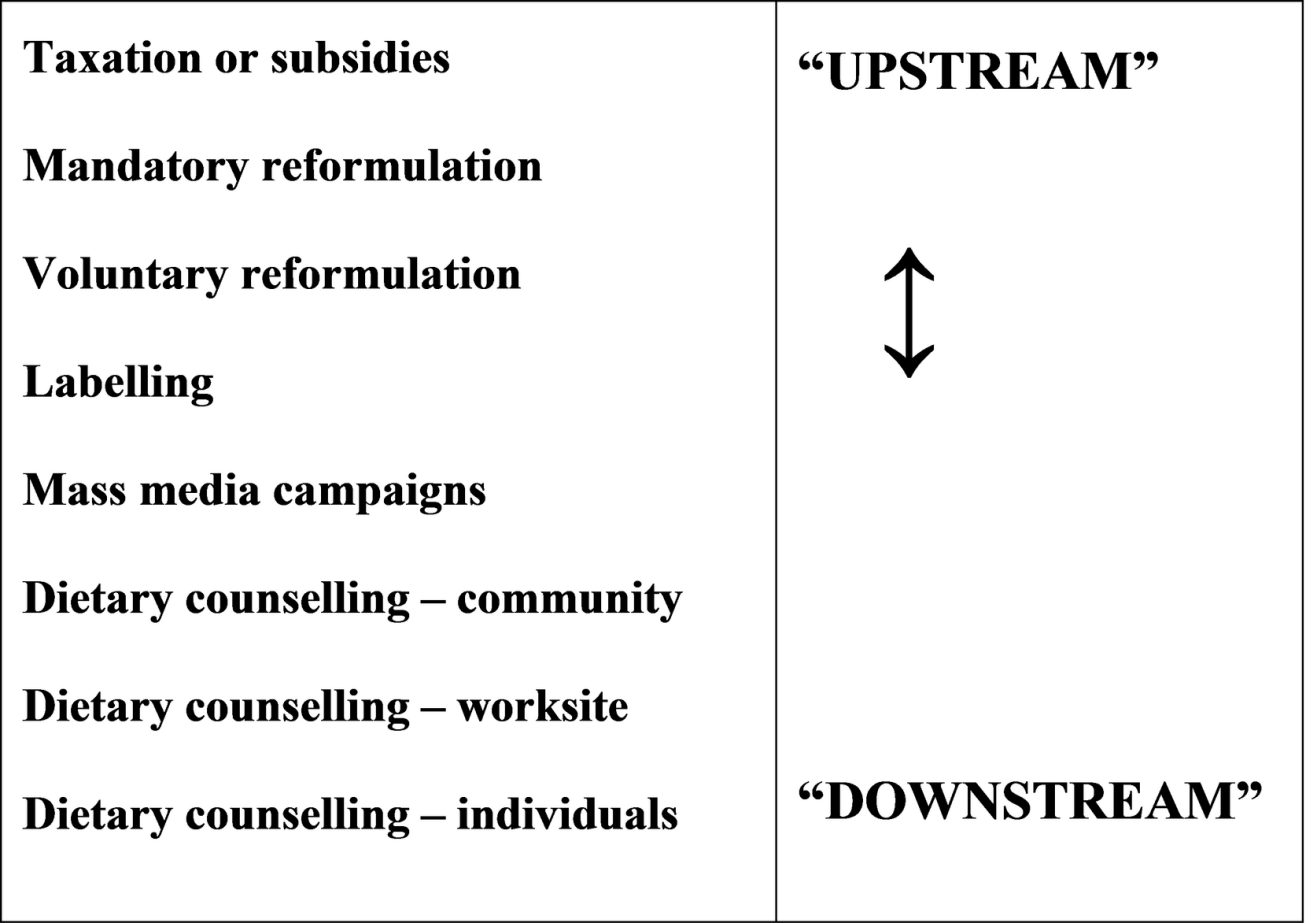
Interventions classified on the upstream / downstream continuum.

Interventions were then categorised according to their position in the McLaren et al. (2010) continuum from “upstream” to “downstream” (Fig 1).[21]

Multi-component interventions were considered separately.

### Patient involvement

Individual patients were not involved in this research; this is a secondary analysis of published data.

## Results

**Fig 2:**
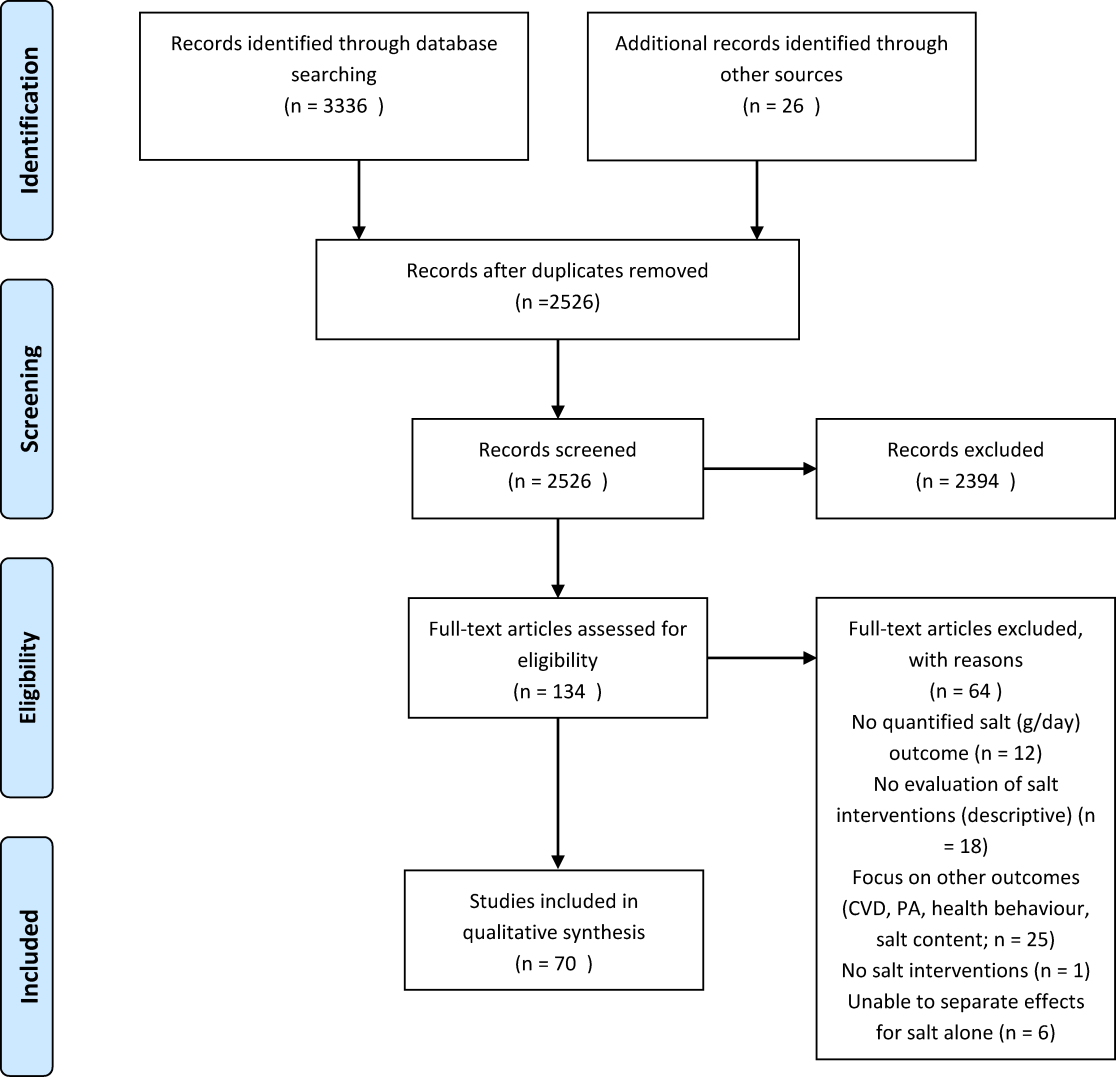
PRISMA flowchart.

**Fig 3:**
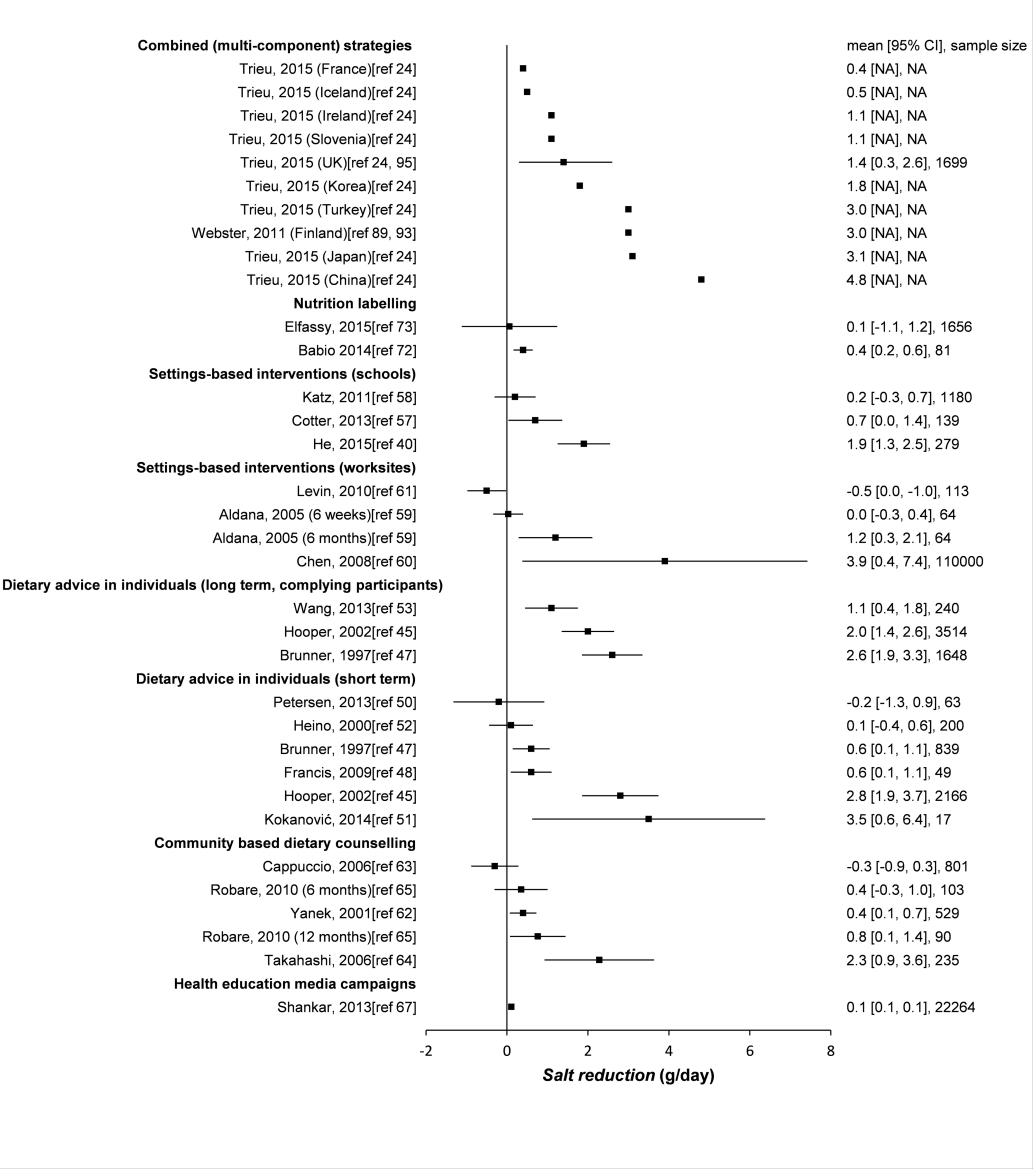
Effectiveness of interventions to reduce salt intake (empirical studies). Forest plot of the empirical studies that were included in this systematic review. Negative values of salt reduction are interpreted as reported increase in salt consumption. For most combined interventions the sample size and confidence intervals were not reported. NA denotes not applicable or not reported.

**Fig 4:**
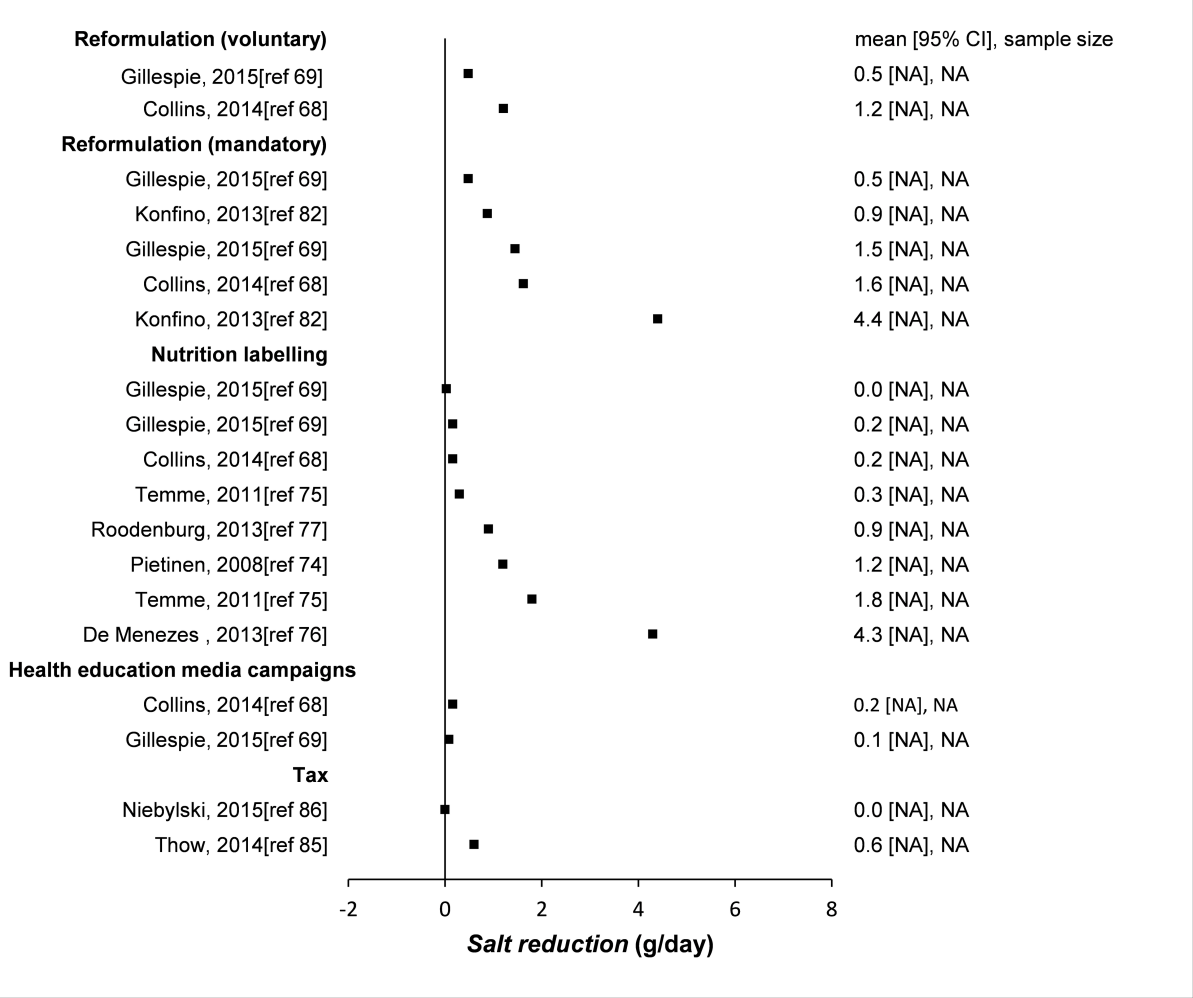
Effectiveness of interventions to reduce salt intake (modelling studies). Forest plot of the modelling studies that were included in this systematic review. Because of the different modelling approaches in these studies, their uncertainty measures are not comparable. Therefore we do not plot them in this graph. Different scenarios were considered for different studies. NA denotes not applicable or not reported.

The literature search identified 3336 potentially relevant papers. An additional 26 papers were identified through other sources, including reference lists and key informants. After removing 836 duplicates, 2526 publications were left to be screened by title and abstract, after which 134 full-text papers were assessed for eligibility. A total of 70 papers were finally included (49 empirical studies and 21 modelling studies, Fig 2). The interventions and their effect sizes are presented in Fig 3 (empirical studies) and Fig 4 (modelling studies).

### Dietary counselling–individual level (Table 2)

Nine empirical studies (two of good quality;[45–46] five of fair quality;[47–51] and two of poor quality [52–53]), and three modelling studies (all of good quality [54–56]) investigated the effect on salt intake of dietary counselling targeted at consenting individuals.

Two separate meta-analyses investigated the effect of dietary advice on salt intake. The first included eleven randomised controlled trials (RCTs) and found a 1.8g/day salt reduction after up to 18 months of dietary advice.[47] The second meta-analysis included eight RCTs and reported an overall reduction in salt consumption of 2.8g/day at 12 months and 2g/day up to 60 months.[45] The two meta-analyses overlapped in respect of only three studies.

One additional RCT found a statistically significant net reduction of 0.6g/day between the groups,[48] whilst a second RCT found no effect between the control and intervention group.[50]

All three modelling studies predicted that dietary advice is less effective in reducing the disease burden of high salt intake, only gaining 180–2,600 quality-adjusted life years (QALYs) compared to other interventions (7,900–195,000 QALYs).[54–56]

### Dietary counselling–school based and worksite interventions (Table 3)

Three school-based interventions (one of good quality;[40] one of fair quality;[57] one of poor quality [58]) and three worksite-based studies (all of fair quality) were included.[59–61] No modelling studies were identified for this section.

#### Schools

A nutrition programme in schools aimed at distinguishing between healthy and less healthy choices reported a non-significant reduction.[58] In the second school based RCT, the practical intervention group achieved a significant net reduction of 0.7g/day compared with the control group.[57] In a cluster RCT in China, education and training significantly reduced salt intake by a mean of −1.9 g/day in 279 school children (and −2.9 g/day in adult family members).[40]

#### Worksites

A randomised trial of a chronic disease prevention programme achieved a net reduction of 1.2g/day between the intervention and control group (P = 0.01).[59] A factory-based intervention study in China assessed health education aimed at altering diet, together with a high-risk strategy of hypertension control. Salt intake was reduced by 3.9g/day from a mean of 16g/day (P<0.05).[60]

### Dietary counselling–community level (Table 4)

Four empirical studies and one review, all of fair quality,[62–66] investigated community based dietary counselling. One study reported a statistically significant difference of -0.4g/day in salt intake between the intervention and control groups.[62] Two intervention trials of nutrition education reported significant reductions of 0.7g/day and 2.2g/day reductions respectively in salt intake after 12 months.[63–64] One RCT reported a favourable trend; however, this was non-significant and could have been caused by contamination between the groups.[63]

### Mass media campaigns (Table 5)

One empirical study of fair quality [67] and five modelling studies; four of good quality[56, 68–70] and one of fair quality[71] were included.

The UK FSA salt reduction programme involved media campaigns to discourage table salt use, plus sustained pressure on industry to reformulate. Although salt consumption declined by 0.9g/day using spot urinary sodium readings from 2003–2007, the media contribution was unclear but likely modest.[67]

The modelling studies likewise suggested media campaigns were generally considered less effective than food labelling or reformulation.[56, 69–71] The Change4Life campaign in the UK was predicted to reduce salt intake by 0.16g/day, less than labelling or reformulation.[68] Gillespie et al. (2015) similarly estimated that social marketing might modestly reduce salt consumption by 0.03g/day to 0.13g/day.[69]

### Nutrition labelling (Table 6)

Two empirical studies, both of poor quality, investigated the effect of nutrition labelling on salt intake [72–73]. Reduced salt intake was not observed in participants who reported frequent vs. non-frequent label use (7.7g/day vs. 7.6g/day).[73]

Ten modelling studies also examined labelling, four of good quality[56,68–70] and two of fair quality.[71, 74–77] These suggested that labelling might modestly reduce UK salt intake by 0.03g/day to 0.16g/day [68, 69]; much less than the 0.9g/day estimated by Roodenburg et al. (2013).[77] Another study suggested that salt intake might be lowered by 1.2g/day if the population were to choose products labelled as low-salt, or increased by 1.6g/day if they choose products labelled as high salt content.[74]

### Reformulation (Table 7)

Very few studies which focused on reformulation included quantified results of salt intake. In one empirical Taiwanese study of fair quality,[78] salt was enriched with potassium in the intervention group and their outcomes were an apparent reduction in cardiovascular deaths by 41%, compared to the control group rather than salt intake. Furthermore, people in the intervention group lived 0.3–0.9 years longer.[78]

Fourteen modelling studies evaluated reformulation, eleven of good quality[41, 54–56, 68–70, 79–82] and three of fair quality[71, 83, 84]. Mandatory reformulation could consistently achieve bigger salt reductions than voluntary reformulation; 1.6g/day compared with 1.2g/day;[68] and 1.4g/day versus 0.5g/day.[69] Mandatory reformulation might also achieve higher reductions in disability-adjusted life years (DALYs) and QALYs compared to voluntary reformulation.[54, 56, 79]

In the Netherlands, reformulation of processed foods was predicted to reduce median salt intake by 2.3g/day,[84] compared with a 0.9g/day from a two-year salt reformulation initiative in Argentina.[82]

### Fiscal interventions (Table 8)

Two systematic reviews of fair quality [85, 86] included three modelling studies eligible for this review. Furthermore, three additional tax modelling studies were included, all of good quality.[56, 81, 87] Two studies included in Niebylski et al’s. systematic review (2015) modelled a 1% tax on salty snacks or on cheese and butter; neither reduced salt consumption.[86] Another modelling study suggested that a very high (40%) tax might achieve a 6% reduction in salt consumption (0.6g/day).[81]

One modelling study predicted that a 20% tax on major dietary sodium sources might prevent or postpone 2000 deaths annually,[87] whilst Nghiem et al. (2015) predicted that a sodium tax might gain more QALYs than other interventions.[56]

### Multi-component interventions (Table 9 and Table 10)

Fifteen papers were included under multi-component interventions. Most studies came from Japan, Finland and the UK. Two were of good quality;[88, 89] ten of fair quality;[24, 43, 89–96] and four of poor quality.[97–100]

Four studies were included which presented dietary salt intake and linked to papers describing the interventions; (one of good quality;[25]; two of fair quality;[101, 102] and one of poor quality.[103]

#### Japan

The Japanese government initiated a sustained campaign in the 1960s.[26] Over the following decade, mean salt intake fell from 13.5g/day to 12.1g/day overall (and from 18g/day to 14g/day in Northern Japan). Miura et al. (2000) reported that salt intake subsequently decreased from 14.5g/day in 1972 to 10.6g/day in 2010, a fall of almost 4g/day [103]. Stroke mortality was predicted to fall by 80%.[90, 93]

#### Finland

Starting in 1978, Finland pursued a comprehensive salt reduction strategy using mass media campaigns, mandatory labelling and voluntary reformulation by the food industry. Population salt consumption was monitored regularly by using 24h urinary assessment and dietary survey data.[72] By 2007, salt intake had reduced by approximately 4g/day, from 13 to 8.3g/day in men, and from 11 to 7g/day in women.[24, 25] Stroke and coronary heart disease (CHD) mortality fell by over 75% during that period.[90]

#### United Kingdom

The UK salt reduction strategy included voluntary reformulation, a consumer awareness campaign, food labelling, target settings and population monitoring.[95] By 2011, population salt intake, measured by 24h urinary sodium excretion, had decreased by 1.4g/day (9.5g/day to 8.1g/day)[88]. He et al. (2014b) estimated that this might reduce stroke and coronary heart disease mortality by some 36%.[88]

Other countries have implemented several strategies including labelling, media campaigns and voluntary reformulation and effect sizes ranged from -0.4g/day in France [24, 93] to -4.8g/day in China [24, 102].

#### Modelling studies of combined interventions

Six modelling studies investigated the effect of multi-component interventions, three were of good quality;[70, 104, 105] whilst three others were of fair quality.[70, 106, 107]

Several modelling studies consistently suggested that multi-component salt reduction strategies (e.g. labelling, health promotion and reformulation) would be more effective than any single intervention.[70, 71] For instance, Gase et al. (2011) suggested that using labelling, promotion, subsidies and provision of low sodium options could lead to a 0.7–1.8g/day reduction.[106]

## Discussion

### Main results

This systematic review of salt reduction interventions suggests that comprehensive strategies could generally achieve the biggest reductions in salt consumption across an entire population, approximately 4g/day in Finland and Japan, 3g/day in Turkey and 1.3g/day recently in the UK. Mandatory reformulation alone could achieve a reduction of approximately 1.4g/day, followed by voluntary reformulation (median 0.7g/day) school interventions (0.7g/day) and worksite interventions (+0.5g/day). Smaller population benefits were generally achieved by short-term dietary advice (0.6g/day), community-based counselling (0.3g/day), nutrition labelling (0.4g/day), and health education media campaigns (-0.1g/day). Although dietary advice to individuals achieved a -2g/day reduction, this required optimal research trial conditions (smaller reductions might be anticipated in unselected individuals).

### Comparison with other research

Geoffrey Rose famously argued that a greater net benefit came from the population-wide approach, (achieving a small effect in a large number of people) when compared with targeting high risk individuals (a large effect but only achieved in a small number of people).[32]

#### Multi-component interventions

Multi-component salt reduction strategies involving a series of structural initiatives together with campaigns to increase population awareness have been successful in Japan and Finland where they substantially reduced dietary salt consumption, and associated high stroke and cardiovascular disease mortality rates. In Finland, some credit should also go to other dietary changes e.g. fat quality.[108]

Between 2003 and 2010, a multi-component approach in the UK including voluntary reformulation and political pressure on industry to agree category-specific targets achieved some success (1.3g/day reduction in population salt consumption over 8 years to 8.1g/day in 2011). Interestingly, pre-existing health inequalities in salt consumption persisted.[29] However, from 2010, the Responsibility Deal simply advocated a voluntary scheme. This was ineffective, and MacGregor therefore subsequently recommended mandatory reformulation.[31] Other useful reductions were demonstrated in other countries mostly using dietary surveys and some from grey literature. However, the -4.8g/day reduction reported in China appears extra-ordinarily large and perhaps merits some caution [24]. Multi-component interventions clearly have more potential than single interventions, and synergies might be anticipated. [13,93] Similarly powerful benefits have also been observed with comprehensive strategies for tobacco control and alcohol reduction.[35,36]

#### Reformulation

In high income countries, the majority of dietary salt intake comes in processed food (75%) and reformulation can be very effective in reducing salt consumption.[109] Though mandatory reformulation is more powerful, most countries currently use voluntary reformulation.[54,56,68,69,110] Success may then be very dependent on the degree of political pressure applied to the food industry and on regular, independent monitoring, as recently achieved in the UK. [111,112]

#### Food labelling

Nutrition labelling can be potentially effective, as demonstrated in Finland [72] and Brazil.[74] Nutrition labelling allows consumers to make informed choices whilst also putting pressure on the food industry to reformulate.[89] However, interpretation of labels depends on health literacy and different labelling systems may confuse consumers,[113] and reinforce inequalities.[29]. Consumers generally want simple (traffic light) labels which are easier to understand.[76,77,113,114]

Dietary interventions in diverse settings: communities, worksites, schools and homes.

Dietary interventions can be delivered at different levels, such as communities, worksites, schools or to individuals. However, effectiveness varies widely.[45,47,50] Furthermore, the benefits of dietary counselling decrease over time and are thus generally not sustainable; much smaller reductions might therefore be anticipated in unselected individuals in the general population.[44] Furthermore, for many individuals, issues such as competing priorities and financial constraints might reduce compliance and adherence,[8,13,21,22] and thus reduce net population benefits.

#### Mass media campaigns

Few empirical studies have examined salt media campaigns. However, benefits appear to be generally modest.[56, 67,68,69,115] or negligible.[111] Many individuals may not perceive any personal relevance and hence fail to engage in any behaviour change.[22,116,117]

#### Taxation

Price increases can powerfully reduce consumption of tobacco or alcohol.[35,36] However, salt is cheap, and a substantial tax of at least 40% might be needed to reduce consumption by just 6%.[81,118]

### Public health benefits and cost-effectiveness

Most economic analyses have consistently predicted substantial reductions in cardiovascular mortality, and consequent gains in life-years, QALYs, DALYs and healthcare savings. This is consistent with the growing evidence that population-wide prevention policies can often be powerful, rapid, equitable and cost-saving.[38,119–122]

Several modelling studies also investigated the cost-effectiveness of the salt interventions described above. Mandatory and voluntary reformulation appeared far more cost-effective than labelling or [54,55,68] dietary advice targeting individuals.[122]

### Strengths and limitations

This systematic review has multiple strengths. Firstly, two independent reviewers screened all papers and assessed quality using appropriate validated tools. Secondly, the inclusion of modelling studies (presented separately) adds value by allowing the evaluation of certain interventions where empirical studies failed (e.g. labelling). In addition, we recorded the effect size used in each modelling paper together with the source reference. Furthermore, most of the better quality modelling studies confirmed the superiority of upstream approaches. Finally, the studies reviewed included a wide variety of interventions, thus providing a useful spread of estimates.

Our review also has limitations. We were unable to conduct a formal meta-analysis due to the profound heterogeneity of the diverse studies, many of which included multiple interventions. Furthermore, studies were only included if the full text was available in English (15 non-English papers were excluded). We also had to exclude two potentially relevant studies which lacked the full text.[123,124] Publication bias remains possible, potentially over-estimating the true effect of some interventions. The primary outcome of this study was dietary intake (consumption); we excluded studies considering other dietary behaviours such as awareness, knowledge, preferences or purchasing behaviour. Also, the positive benefits of policy changes may sometimes appear larger if favourable underlying secular trends have not been formally considered. Furthermore, we did not contact authors for missing data. However, all the key information was presented in all but two papers. [123,124] Finally, generalization of the results should be cautioned as countries may vary in baseline salt intake.

### Socio-economic Inequalities

More deprived groups more often consume foods high in salt, (and sugar and fat); all are associated with poor health.[125–127] These inequalities persist in Britain [28,29] and Italy.[128]

Downstream interventions focused on individuals typically widen inequalities whereas upstream “structural” interventions may reduce inequalities.[33,129,130]

### Future research

This review highlights the greater power of combined (multi-component) strategies, mandatory reformulation and traffic light labelling. Most were cost-effective and many were cost-saving. However, the feasibility of implementing policy changes also deserves further study. Many factors can facilitate or obstruct successful policy development, notably including political feasibility and stakeholder influence.[114,131,132]

Stoeckle and Zola’s “upstream”/”downstream” concept was disseminated by John McKinlay,[133] critiqued by Krieger,[134] and then refined as a structural/agentic continuum by McLaren et al 2010.[21] To test our effectiveness hierarchy hypothesis, one ideally needs to quantify the “average” effect of each category of salt reduction intervention. Yet, the limited number and heterogeneity of these studies precludes a formal meta-analysis. However, the consistency with the effectiveness hierarchies demonstrated by tobacco and alcohol control interventions is encouraging. The effectiveness hierarchy hypothesis now clearly needs to be tested in other fields.

## Conclusions

There are clear implications for public health. The biggest population-wide reductions in salt consumption were consistently achieved by comprehensive multi-component strategies involving “upstream” population-wide policies (regulation, mandatory reformulation, and food labelling).”Downstream” individually-based interventions appear relatively weak (e.g. dietary counselling to individuals and school children, and media campaigns in isolation).

This ‘effectiveness hierarchy’ might deserve greater emphasis on the agendas of the WHO and other global health organizations reviewing action plans for NCD prevention.

## Supporting information

S1 Table PRISMA checklist. (DOCX)

S2 Table Full data extraction tables empirical and modelling studies. (DOC)

S1 File Research protocol. (DOCX)
