# Supplementary material for: Systematic review of dietary salt reduction policies: Evidence for an effectiveness hierarchy?
Source: PLoS One. 2017 May 18;12(5):e0177535. doi: 10.1371/journal.pone.0177535 (PMC5436672; doi:10.1371/journal.pone.0177535)
Supplement: S2 Table — (DOC) [file pone.0177535.s002.doc]

**Supplementary file 3.**

**Table 12. All included empirical studies**

**12A. Dietary counselling** **(individuals)**

| **Study ID** | **Study Design** | **Study Aim** | **Policy(ies) Analysed** | **Participants & sample size** | **Geographical Scope** | **Methods** | **Outcomes** | **Study Limitations** | **Comments** |
| --- | --- | --- | --- | --- | --- | --- | --- | --- | --- |
| Hooper et al. (2002)^45^ | Systematic review and meta-analysis of RCTs | To assess the long term effects of advice to restrict dietary sodium in adults with and without hypertension | Dietary advice |  | US, Australia, Italy, New Zealand, UK, | Cochrane library, Medline, Embase, and bibliographies were searched for relevant papers | 11 studies were included in the meta-analysis. Reductions in urinary 24 hour sodium excretion were found at both intermediate (48.9 mmol/24 hours, 65.4 to 32.5) and late follow up (35.5 mmol/24 hours, 47.2 to 23.9). We identified significant heterogeneity in both analyses that was not explained by trial quality. One trial in people without hypertension found that at seven years sodium excretion in a small subset of the original sample was similar in intervention and control groups | They were not able to assess the overall effect of advice to reduce dietary sodium on mortality or morbidity as too few events occurred. The completeness of urine samples is not known and it has been suggested that less salty foods were eaten on collection days in the trial of Thaler et al. |  |
| **Study ID** | **Study Design** | **Study Aim** | **Policy(ies) Analysed** | **Participants & sample size** | **Geographical Scope** | **Methods** | **Outcomes** | **Study Limitations** | **Comments** |
| Appel et al. (2003)^46^ | Randomised trial | To determine the effect on BP of 2 multicomponent, behavioural interventions | 1. Dietary advice | 810 adults | US | Participants were randomized to one of 3 intervention groups: (1) "established," a behavioural intervention that implemented established recommendations (n = 268); (2) "established plus DASH," which also implemented the DASH diet (n = 269); and (3) an "advice only" comparison group (n = 273) | Also, based on 24-hour dietary recall data, both behavioural interventions significantly reduced sodium intake in comparison with the advice only group (P value = 0.01).  Advice group:   - Baseline: 173.3 (66.7) mEq/24h - 6 months: 152.8 (66.3) mEq/24 h   Established:   - Baseline: 167.8 (70.0) mEq/24 h - 6 months: 136.2 (64.6) mEq/24 h   Established + DASH:   - Baseline: 178.2 (78.9) mEq/24 h - 6 months: 145.6 (71.6) mEq/24 h |  |  |
| **Study ID** | **Study Design** | **Study Aim** | **Policy(ies) Analysed** | **Participants & sample size** | **Geographical Scope** | **Methods** | **Outcomes** | **Study Limitations** | **Comments** |
| Brunner et al. (1997)^47^ | Meta-analysis of RCTs | To evaluate the effectiveness of dietary advice in primary prevention of chronic disease | 1. Dietary behaviour interventions | 6893 participants with 3736 in the intervention groups | UK, US, Netherlands, and Australia | A meta-analysis was conducted of 17 randomized controlled trials of dietary behaviour interventions of at least 3 months' duration. Results were analyzed as changes in reported dietary fat intakes and biomedical measures (serum cholesterol, urinary sodium, systolic and diastolic blood pressure) in the intervention group minus changes in the control group at 3 to 6 months and 9 to 18 months of follow-up | At 3 to 6 months, the Hypertension Prevention Trial provided two data points: (1) the sodium calorie restriction arm and (2) the sodium restriction and reduced sodium/increased potassium arms, which were pooled. The overall mean net reduction of some 32 mmol/24 hours was equivalent to 1.9 g NaCl, or a 20% reduction in salt intake.  The heterogeneity test was highly significant (P < .0005) for the 3- to 6-month trials, because the net reduction of 59 (95% CI = 45, 72) mmol/24 hours was an outlier result. At 9 to 18 months, the summary effect for the two trials with standard errors was somewhat larger than at 3 to 6 months |  |  |
| **Study ID** | **Study Design** | **Study Aim** | **Policy(ies) Analysed** | **Participants & sample size** | **Geographical Scope** | **Methods** | **Outcomes** | **Study Limitations** | **Comments** |
| Francis & Taylor (2009)^48^ | Randomised control group study | To implement a heart-healthy diet-education program designed using the needs and preferences of the target audience | 1. Dietary counselling | 58 women (30 control; 28 intervention) ages 54 to 83 years | US | 90-day program. Data were collected using the Mini Nutritional Assessment, three 3-day food records, and program evaluations. The intervention group received two individual registered dietitian–led in-home education sessions and the control group received education material mailings (Visits 2 and 3). Pretested education materials were used. Visits/mailings were scheduled 28 to 30 days apart. Variables measured included cardiovascular disease related dietary practices and dietary status (Mini Nutritional Assessment) | Intervention sodium consumption decreased significantly (P0.020) from record 1 to record 3. The reduction in control group participants’ sodium intake was not significant.  *Intervention*  Mean ± SEM (mg), P-value  Record 1: 2,791±192.7 0.020e  Record 2: 2,375±123.7 0.067  Record 3: 2,359±167.1 0.937  *Control*  Mean ± SEM (mg), P-value  Record 1: 2,473±215.5 0.323  Record 2: 2,447±149.9 0.880  Record 3: 2,276±142.9 0.284 |  | Control group received emails so still some form of education |
| **Study ID** | **Study Design** | **Study Aim** | **Policy(ies) Analysed** | **Participants & sample size** | **Geographical Scope** | **Methods** | **Outcomes** | **Study Limitations** | **Comments** |
| Parekh et al. (2012)^49^ | Randomised controlled trial | To evaluate the effectiveness of a minimal intervention on multiple lifestyle factors such as diet, physical activity, smoking and alcohol, delivered through general practice, using computer-tailored feedback | 1. Health promotion - computer tailored advice | 2306 adult patients | Australia | Patients visiting 21 general practitioners in Brisbane, Australia, were surveyed about ten health behaviours that are risk factors for chronic, non-communicable diseases. Those who completed the self-administered baseline questionnaire entered a RCT, with The intervention group received computer-tailored printed advice, targeting those health behaviours for which respondents were not meeting current recommendations. The control group received an individualised letter and tailored information sheets about the five health protective behaviours not included in the Prudence Score. The primary outcome was change in summary lifestyle score (Prudence Score) and individual health behaviours at three months. A repeated measures analysis compared change in these outcomes in intervention and control groups after adjusting for age and education | 1711 (76%) returned the follow up questionnaire at 3 months.  *Salt*   - Intervention +5.43 net change - Control +1.23 net change   Significant changes between groups were observed for reduced salt intake (OR 1.19, CI 1.05-1.38). The intervention group were 20% more likely to reduce salt intake | Dichotomous scoring system for health behaviours, where sub-threshold change in behaviour remains undetected. Use of self-reported data |  |
| **Study ID** | **Study Design** | **Study Aim** | **Policy(ies) Analysed** | **Participants & sample size** | **Geographical Scope** | **Methods** | **Outcomes** | **Study Limitations** | **Comments** |
| Petersen et al. (2013)^50^ | Randomised controlled trial | To investigate whether urinary sodium excretion can be reduced by educating people with T2DM to read food labels and choose low sodium products | 1. Nutrition education | 78 men (n=49) and women (n=29) with T2DM | Australia | In a 3 month RCT, participants were recruited from a Diabetes Centre at a University teaching hospital. The intervention group was educated in a single session to use the nutrition information panel on food labels to choose products which complied with the Food Standards Australia New Zealand (FSANZ) guideline of <120 mg sodium/100 g food. The control group continued on their usual diet. 24h urinary sodium excretion was performed at baseline and 3 months | At baseline reported sodium intake was 2714 ± 1279 mg/day.  **Reported dietary sodium intake (mg/24h)**  *Intervention*   - Baseline: 2479 ± 190 - 3 months: 2416 ± 181 - Change: -63 ± 249   *Control*   - Baseline: 2941 ± 237 - 3 months: 2240 ± 149 - Change: -701 ± 190   **Urinary sodium excretion**  ***mmol/24h***  *Intervention*   - Baseline: 174 ± 13 - 3 months: 175 ± 13 - Change: 1 ± 15   *Control*   - Baseline: 167 ± 15 - 3 months: 161 ± 13 - Change: -6 ± 14   ***mg/day***  *Intervention*   - Baseline: 4002 ± 299 - 3 months: 4025 ± 299 - Change: 23 ± 345   *Control*   - Baseline: 3841 ± 345 - 3 months: 3703 ± 299 - Change: 138 ± 322   Urinary sodium excretion was unchanged in the intervention (+1 ± 15 mmol/24 h) and control group (6 ± 14 mmol/24 h), and there was no between group difference (p > 0.05). There was a statistically significant reduction in reported sodium intake after 3 months in the control group only (p < 0.05). However, when adjusted for energy intake, sodium intake (mg) per 1000 kJ of energy was not different between the groups at 3 months | Not many low sodium products available in the food supply during this study |  |
| **Study ID** | **Study Design** | **Study Aim** | **Policy(ies) Analysed** | **Participants & sample size** | **Geographical Scope** | **Methods** | **Outcomes** | **Study Limitations** | **Comments** |
| Kokanović et al. (2014)^51^ | Before and after study | To assess eating habits of adolescent population, diagnosed with one or more cardiovascular risks before and two months after individual dietary intervention | 1. Nutrition education | 17 adolescents aged 14.5 years | Croatia | Food frequency questionnaire, anthropometric measurements, blood pressure and biochemical measurements with individual nutrition education were conducted on a group of adolescents | The difference in intake on initial and control examination was statistically significant for intake of sodium p=0.013.   - Initial examination: 7574mg - Control examination: 6147mg - Difference: -1426mg - %: -18.8% |  | No control group |
| **Study ID** | **Study Design** | **Study Aim** | **Policy(ies) Analysed** | **Participants & sample size** | **Geographical Scope** | **Methods** | **Outcomes** | **Study Limitations** | **Comments** |
| Heino et al. (2000)^52^ | Prospective randomize long-term trial | To examine sodium intake and dietary sodium sources of 1-5-y-old children in a prospective, randomized long-term coronary heart disease prevention trial, focused on dietary fat modification | Dietary counselling | 200 children aged between 1-5 years old | Finland | Counselling included no advice about reducing salt in the children’s diets. Food consumption of the children was recorded for 3 consecutive days at the age of 13 months and for 4 consecutive days at the ages of 3 and 5 y. Sodium intakes were calculated using the Micro Nutrica program | The daily sodium intake of the children (intervention children and control children combined) was 1600 ± 523 mg (4.0 ± 1.3 g NaCl) at the age of 13 months, 1900 ± 504 mg (4.8 ± 1.3 g NaCl) at the age of 3 y and 2200 ± 531 mg (5.5 ± 1.3 g NaCl) at the age of 5 y.  **Sodium intake mg/d**  *Intervention children*   - 13 months: 1621 ± 489 - 3 years: 1943 ± 485 - 5 years: 2244 ± 521   *Control children*   - 13 months: 1560 ± 563 - 3 years: 1899 ± 523 - 5 years: 2218 ± 543   No significant differences found | The assessment of children’s sodium intake is prone to many errors. First, food records may be unreliable at this age. The calculated intake of sodium at the age of 13 months, derived from “recipe salt”, overestimates the intake. Intra-individual day-to-day variations in food consumption | Intervention was on fat but sodium was measured |
| **Study ID** | **Study Design** | **Study Aim** | **Policy(ies) Analysed** | **Participants & sample size** | **Geographical Scope** | **Methods** | **Outcomes** | **Study Limitations** | **Comments** |
| Wang et al. (2013)^53^ | Randomised controlled trial | To examine patterns and amount of daily sodium intake among participants with metabolic syndrome enrolled in a one-year dietary intervention study | Health promotion – dietary counselling | 244 participants with metabolic syndrome | US | Participants with metabolic syndrome were randomized to either a high fiber diet or the American Heart Association diet (control) in a dietary intervention trial to lose weight and improve dietary quality. Three 24-hour dietary recalls were collected at each visit which provided meal patterns and nutrient data, including sodium intake. A secondary data analysis was conducted to examine sodium consumption patterns at baseline and at one-year study visits. Sodium consumption patterns over time were examined using linear mixed models | Follow-up for the one-year dietary intervention revealed that. Average sodium intake decreased from 2,994 mg/day at baseline to 2,558 mg/day at one-year (P < 0.001). At the one-year visit, sodium intake was consistently reduced; however a significant difference was only observed between males (3051 ± 146 mg/d) and females (2380 ± 88 mg/d; p < 0.001). After the one-year intervention, subjects consumed lower amounts of sodium when they ate at home with significant differences observed at lunch (1062 ± 47 versus 883 ± 49 mg; p < 0.05) and dinner (1281 ± 33 versus 1058 ± 36 mg; p < 0.05). Sodium intake when subjects ate away from home also decreased after the one-year intervention; however, a significant difference was only observed at lunch (1102 ± 54 versus 841 ± 56 mg; p < 0.05). Compared to baseline, sodium intake after the one-year intervention increased when subjects ate at restaurants/fast food chains with a significant difference for dinner (1610 ± 80 versus 1861 ± 82 mg; p < 0.05). However, the sodium density per meal between meal locations and the day of week were similar except for breakfast when consumed away from home or at restaurant/fast food (Baseline:1.68 ± 0.12 versus 1.29 ± 0.05 mg/kcal; p < 0.05; One-year: 1.85 ± 0.19 versus 1.28 ± 0.06 mg/kcal p < 0.05) | Obese participants with metabolic syndrome – not able to generalize to US population. Sodium intake measured through 24h recalls – sodium intake likely to be underestimated. Study was not focused exclusively on reducing sodium intake | Conditions are high fibre trial or AHA diet (control) |

**12B. Dietary counselling** **(worksite/schools)**

| **Study ID** | **Study Design** | **Study Aim** | **Policy(ies) Analysed** | **Participants & sample size** | **Geographical Scope** | **Methods** | **Outcomes** | **Study Limitations** | **Comments** |
| --- | --- | --- | --- | --- | --- | --- | --- | --- | --- |
| He et al. (2015)^40^ | Cluster Randomised controlled trial | To determine whether an education programme targeted at schoolchildren could lower salt intake in children and their families | Health education programme in schools | 279 children in grade 5 of primary school | Northern China | 28 primary schools in urban Changzhi, northern China were selected. Children in the intervention group were educated on the harmful effects of salt and how to reduce salt intake within the schools’ usual health education lessons. Children then delivered the salt reduction message to their families. The intervention lasted for one school term (about 3.5 months). Children in the control group carried on with their usual health education lessons as in the curriculum, and these lessons did not contain information on salt | At baseline, the mean salt intake in children was 7.3 (SE 0.3) g/day in the intervention group and 6.8 (SE 0.3) g/day in the control group. In adult family members the salt intakes were 12.6 (SE 0.4) and 11.3 (SE 0.4) g/day, respectively. During the study there was a reduction in salt intake in the intervention group, whereas in the control group salt intake increased. The mean effect on salt intake for intervention versus control group was −1.9 g/day (95% confidence interval −2.6 to −1.3 g/day; P<0.001) in children and −2.9 g/day (−3.7 to −2.2 g/day; P<0.001) in adults. The mean effect on systolic blood pressure was −0.8 mm Hg (−3.0 to 1.5 mm Hg; P=0.51) in children and −2.3 mm Hg (−4.5 to −0.04 mm Hg; P<0.05) in adults | Difficult to know whether there was under collection of urine during 24h urine collections – this potentially may have led to underestimation of salt intake |  |
| **Study ID** | **Study Design** | **Study Aim** | **Policy(ies) Analysed** | **Participants & sample size** | **Geographical Scope** | **Methods** | **Outcomes** | **Study Limitations** | **Comments** |
| Cotter et al. (2013)^57^ | Randomised controlled trial | To evaluate the 24-h urinary sodium excretion in children of 10-12 years at a school in the north of Portugal and to examine the influence on salt intake and blood pressure of three different educational interventions for 6 months | 1. Nutrition education | 155 children aged between 10-12 years old | Portugal | Blood pressure (BP) and sodium excretion in 24-h urinary samples (UNa) validated with urinary creatinine were measured in 155 children 10-12 years old belonging to nine classes at baseline and after 6 months of three educational interventions in students from three classes each after parents’ consent was obtained. Interventions consisted in no additional action [control (CTR)], weekly lessons about the dangers of high salt intake [Theoretical (THEOR)] and both lessons and working practices in the school garden of planting, collection of herbs for salt substitution at home [practical (PRACT)] | Population baseline urinary sodium excretion was 132 ± 43mmol/24 h leading to an estimation of mean salt intake of 7.8 ± 2.5 g per day. At the end of the interventional phase no significant differences were found between the final values observed in the three groups and between baseline and final values in each group. The reduction in salt intake was statistically significant in the PRACT group (1.1 g per day), whereas in the CRT and THEOR groups, the reduction, respectively, 0.3 and 0.6 g per day, was not significant.  **Estimated salt intake (g/d)**  *CRT*   - Baseline: 7.7 ± 2.0 - Final: 7.4 ± 3.0 - Change: 0.35 ± 2.42   *THEOR*   - Baseline: 8.1 ± 3.0 - Final: 7.5 ± 3.0 - Change: 0.60 ± 3.24   *PRACT*   - Baseline: 7.5 ± 2.4 - Final: 6.4 ± 2.2 - Change: 1.08 ± 2.47 | Small dimension of the sample. It is generally assumed that 24-h urinary collection is difficult to achieve and because of the common doubts on the completeness of the full day urinary collection |  |
| **Study ID** | **Study Design** | **Study Aim** | **Policy(ies) Analysed** | **Participants & sample size** | **Geographical Scope** | **Methods** | **Outcomes** | **Study Limitations** | **Comments** |
| Katz et al. (2011)^58^ | Randomised controlled trial | To evaluate the effects of a nutrition education program designed to teach elementary school students and their parents, and to distinguish between more healthful and less healthful choices in diverse food categories | Nutrition education | 1180 second, third and fourth grade elementary school students were included, with 628 students in the intervention and 552 in the control group | US | Three schools were assigned to receive the Nutrition Detectives™ program and 2 comparable schools served as controls. The program, delivered by physical education instructors over several sessions in total less than 2 hours, taught the children how to read food labels and detect marketing deceptions, while learning to identify and choose healthful foods. Parents were introduced to the program through written materials sent home and at school functions | There were no statistically significant improvements in dietary patterns from baseline between the intervention (-92.8±765.2mg) and control groups (-17.3±891.9mg) for  sodium intake (p = .44) | The food label quiz used was developed specifically for the program and not previously validated. This study did not show effects on downstream variables within the logic model for obesity, such as dietary patterns and weight. Relatively short duration of several months. Results were obtained from a single school district, potentially limiting generalizability |  |
| **Study ID** | **Study Design** | **Study Aim** | **Policy(ies) Analysed** | **Participants & sample size** | **Geographical Scope** | **Methods** | **Outcomes** | **Study Limitations** | **Comments** |
| Aldana et al. (2005)^59^ | Randomised clinical trial | To determine the behavioural and clinical impact of a worksite chronic disease prevention program | Health education | 137 working adults | US | Working adults participated in randomized clinical trial of an intensive lifestyle intervention. Nutrition and physical activity behaviour and several chronic disease risk factors were assessed at baseline, 6 weeks, and 6 months | **Sodium (mg)**  *Intervention group*   - Baseline: 3003.0 - ∆6 weeks: -209.3 - ∆6 months: -689.3   *Control group*   - Baseline: 2521.7 - ∆6 weeks: -184.1 - ∆6 months: -197.9   Between group ∆scores (P value)   - 6 weeks: 0.8824 - 6 months: 0.0097   Significant differences in mean change scores were not observed at 6 weeks but they were seen at 6 months |  |  |
| **Study ID** | **Study Design** | **Study Aim** | **Policy(ies) Analysed** | **Participants & sample size** | **Geographical Scope** | **Methods** | **Outcomes** | **Study Limitations** | **Comments** |
| Chen et al. (2008)^60^ | Intervention control trial | To report the effects of these two programmes on blood pressure and changes in morbidity and mortality from coronary heart disease and stroke | 1. Health promotion | >110.000 capital steel and iron company workers in each intervention arm | China | From 1987 to 1995, the cardiovascular intervention trial was launched and conducted in two factories with one as an intervention (factory) site and the other a control site. BFCP was a comprehensive prevention trial in the Fangshan rural area during the period of 1991–1999 | Mean daily salt intake declined from 16.0 to 10.6 g d-1 in the intervention factory, compared with the control factory from 16.9 to 15.4 g d-1, with the net reduction of 3.9 g d-1, which was significantly different (P < 0.05) |  | Comparisons between the intervention and control communities, in both urban and rural China |
| **Study ID** | **Study Design** | **Study Aim** | **Policy(ies) Analysed** | **Participants & sample size** | **Geographical Scope** | **Methods** | **Outcomes** | **Study Limitations** | **Comments** |
| Levin et al. (2009)^61^ | Worksite based dietary intervention study | To examine whether a worksite nutrition programme using a low-fat vegan diet could significantly improve nutritional intake | 1. Health promotion – nutrition education | 109 participants (sixty-five intervention and forty-four control) | US | At the intervention site, participants were asked to follow a low-fat vegan diet and participate in weekly group meetings that included instruction and group support (intervention group). At the control site, participants received no instruction (control group). At weeks 0 and 22, participants completed 3 d dietary records to assess energy and nutrient intake | Intervention group participants significantly increased the reported intake and mean intake (P = 0·04) of sodium compared to the control group.  **Sodium (mg)**  *Intervention group*   - Baseline: 1646.7 ± 44.4 - 22 weeks: 1991.9 ± 66.3 - Mean difference: 345.2 ± 67.4   *Control group*   - Baseline: 1797.1 ± 61.3 - 22 weeks: 1940.4 ± 70.8 - Mean difference: 143.3 ± 63.5   Mean effect size: 201.8 (95% CI 9.2, 394.4; P = 0.04) | Nutrition intake was self-reported. Non-randomised intervention. Trial was limited to 22 weeks |  |

**12C.** **Dietary counselling** **(community)**

| **Study ID** | **Study Design** | **Study Aim** | **Policy(ies) Analysed** | **Participants & sample size** | **Geographical Scope** | **Methods** | **Outcomes** | **Study Limitations** | **Comments** |
| --- | --- | --- | --- | --- | --- | --- | --- | --- | --- |
| Yanek et al. (2001)^62^ | Randomized controlled trial | To test the impact on cardiovascular risk profiles after one year of participation in one of three church-based nutrition and physical activity strategies | 1. Health promotion | 529 African American women aged 40 years and older | US | Women were screened at baseline and after one year of participation. The authors analyzed intention-to-treat within group and between groups using a generalized estimating equations adjustment for intra-church clustering. Because spiritual strategies were added to the standard intervention by participants themselves, the results from both active groups were similar and, thus, combined for comparisons with the self-help group | **Sodium (mg/day)**  *Combined standard and spiritual intervention groups*   - Baseline: 2692 ±1008 - Change: -145 ±25   *Self-help control group*   - Baseline: 2973 ±1212 - Change: -8 ±37   Between group P value = 0.0167 |  | There were 455 people in the intervention group and only 74 in the control group |
| **Study ID** | **Study Design** | **Study Aim** | **Policy(ies) Analysed** | **Participants & sample size** | **Geographical Scope** | **Methods** | **Outcomes** | **Study Limitations** | **Comments** |
| Cappuccio et al. (2006)^63^ | Community-based cluster randomised trial | To establishing the feasibility of salt reduction as a way of reducing BP in twelve rural and semi-urban villages in the Ashanti region of Ghana | 1. Health promotion - education | 1,013 participants from 12 villages | Ghana | A health promotion intervention was provided over 6 months to all villages. Assessments were made at 3 and 6 months. Primary end-points were urinary sodium excretion and BP levels | Salt was often added at the table (52%), more often in rural villages than in semi urban settings (59 v 45%; p < 0.01), although the total salt consumed as measured by urinary sodium was similar (99 v 103 mmol/day). Although at six months the intervention group showed a reduction in systolic (2.54 mmHg [-1.45 to 6.54]) and diastolic (3.95 mmHg [0.78 to 7.11], p = 0.015) BP when compared to control. There was no significant change in UNa. Irrespective of randomisation, there was a consistent and significant relationship between change in UNa and change in systolic BP, when adjusted for confounders. UNa fell in four out of six villages in the intervention group and in 5 out of six villages in the control group. The net intervention effect was therefore a non-significant change in sodium excretion  *Intervention*   - Baseline: 100.9 mmol/24h - 3 months: 94.0 mmol/24h - Baseline: 100.7 mmol/24h - 6 months: 91.8 mmol/24h   *Control*   - Baseline: 103.6 mmol/24h - 3 months: 97.5 mmol/24h - Baseline: 104.2 mmol/24h - 6 months: 89.8 mmol/24h | Low response rate. Time between household survey and the intervention was 18 months | There may have been contamination as the control group did as well or better than the intervention group. Interpret the results with caution |
| **Study ID** | **Study Design** | **Study Aim** | **Policy(ies) Analysed** | **Participants & sample size** | **Geographical Scope** | **Methods** | **Outcomes** | **Study Limitations** | **Comments** |
| Takahashi et al. (2006)^64^ | Community based open randomizer controlled cross-over trial | To assess whether dietary intervention in free-living healthy subjects is effective in improving blood pressure levels | 1. Dietary education | 550 healthy volunteers aged 40-69 years old | Japan | Tailored dietary education was given to the intervention group consisting of four sessions. 5 months later a second dietary assessment was performed | *Nutrition intakes at each point*  Sodium intake in the intervention group decreased by 15 mmol/day (95% CI: -26, -4), but increased by 11 mmol/day (-0, +22) in the control group. This difference in change between the two groups was statistically significant (P = 0.002).  *Sodium excretion and intake among subjects completing urine collections at two points:*  Excretion of sodium in the intervention group decreased by 49 (95% CI: -62, -36) and 11 mmol/day (-25, +4) in the control group. This difference in change between the two groups was statistically significant (P < 0.001). Sodium intake in the intervention group decreased by 13 mmol/day (95% CI: -29, +3) and increased 12mmol/day (95% CI: -5, +28) in the control group (P = 0.040) | Open trial – interaction between groups possible. Subjects were previously exposed to public health campaigns |  |
| **Study ID** | **Study Design** | **Study Aim** | **Policy(ies) Analysed** | **Participants & sample size** | **Geographical Scope** | **Methods** | **Outcomes** | **Study Limitations** | **Comments** |
| Robare et al. (2010)^65^ | Community-based randomized trial | To evaluate a dietary Na reduction trial in a community setting | Nutrition education | 115 hypertensive adults at least 65 years of age | US | Ten-week nutrition intervention activities focused on lifestyle modification to decrease dietary Na intake, under the supervision of a registered dietitian. Twenty-four hour urine specimens were collected at baseline and follow-up visits to determine 24 h urinary Na excretion | At baseline, mean urinary Na was 3128 mg/day. Comparing baseline and 6-month follow-up tests (103 matched pairs), the mean urinary Na dropped to 2990 mg/d at the 6-month follow up. This 138 mg/day reduction was not statistically significant (P = 0·30). When comparing baseline results with the 12-month follow-up (ninety matched pairs), there was a 299 mg/d reduction in urinary Na excretion. This observed decrease was significant (P = 0·03). Mean urinary Na was reduced to 2875 mg/d |  |  |
| **Study ID** | **Study Design** | **Study Aim** | **Policy(ies) Analysed** | **Participants & sample size** | **Geographical Scope** | **Methods** | **Outcomes** | **Study Limitations** | **Comments** |
| Van de Vijver et al. (2012)^66^ | Review | To evaluate the effectiveness of the community-based interventions for CVD prevention programmes in LMIC | 1. Health promotion |  |  | A literature review with searches in the databases of PubMed, EMBASE, CINAHL, LILACS, African Index Medicus and Google Scholar between 1990 and May 2012 | Twenty-six studies involving population-based and high-risk interventions have been included in this review | Prone to publication bias. Follow up for a year and not longer. Most studies implemented a variety of interventions which makes it difficult to separate effects. Control group was missing in half of the studies included. Focus on LMIC which can vary between themselves. Focus on papers in English |  |
| Cappuccio et al. (2006) | RCT |  | 1. Health education and salt reduction | 1013 | Ghana |  | *Blood pressure.* Reduction SBP 2.5 mmHg (1.45 to 6.54), DBP 3.9 mmHg (0.78 7.11)* vs control  *Salt.* No significant reduction in salt intake vs control |  | Already included separately |
| Chen, Wu, and Gu (2008) (urban) | Intervention trial |  | 1. Health education | 120.000 | China |  | *Blood pressure.* Reduction SBP 1.9 mmHg, reduction DBP 2.2 mmHg*  vs control  *Salt.* Reduction in salt intake of 3.9 g/day* vs control |  | Already included separately |
| Yu et al. (1999) | Cross-sectional surveys |  | 1. Health promotion through media and salt reduction | 2000 aged 15-64 years old | China |  | *Blood pressure.* Reduction among men in prevalence in HT 2%,* SBP 0%, among women prevalence of HT 2%,* SBP 2 mmHg  *Salt.* Reduction in salt intake 6.0% |  |  |
| Huang et al. (2011) | Intervention trial |  | 1. Health education | 1632 aged 35 years or older | China |  | *Blood pressure.* Reduction prevalence HT 12.9%* pre vs post  *Salt.* Reduction in salt intake 30%* |  |  |

**12D. Media campaigns**

| **Study ID** | **Study Design** | **Study Aim** | **Policy(ies) Analysed** | **Participants & sample size** | **Geographical Scope** | **Methods** | **Outcomes** | **Study Limitations** | **Comments** |
| --- | --- | --- | --- | --- | --- | --- | --- | --- | --- |
| Shankar et al. (2012)^67^ | Cross-sectional | To provide fresh evidence on the impact of the campaign, by using data on spot urinary sodium readings and socio-demographic variables from the Health Survey for England over 2003–2007 and combining it with food price information from the Expenditure and Food Survey | 1. Salt campaign (potential effect on reformulation and table salt use) |  | UK | Econometrically modelling spot urinary sodium readings from the HSE over the period of 2002–2007, controlling for socio-economic characteristics and relative prices | The results are consistent with a previous hypothesis that the campaign reduced salt intakes by approximately 10%. The impact is shown to be stronger among women than among men. Older cohorts of men show a larger response to the salt campaign compared to younger cohorts, while among women, younger cohorts respond more strongly than older cohorts  *Sodium*   - 2003: 109.03 mmol/l - 2004: 111.28 mmol/l - 2005: 99.94 mmol/l - 2006: 97.49 mmol/l - 2007: 94.16 mmol/l   Difference in mmol/l between 2003-2007 = 13.5% | Spot urinary sodium.  Small sample size.  No control group | The 10% might be for reformulation and the use of salt during food preparation/ consumption |

**12E. Labelling**

| **Study ID** | **Study Design** | **Study Aim** | **Policy(ies) Analysed** | **Participants & sample size** | **Geographical Scope** | **Methods** | **Outcomes** | **Study Limitations** | **Comments** |
| --- | --- | --- | --- | --- | --- | --- | --- | --- | --- |
| Babio et al. (2013)^72^ | Randomised cross-over trial | To compare, in adolescents, two models of front-of-pack Guideline Daily Amounts (GDA) labels in terms of (i) friendliness and acceptance and (ii) the ability to choose a diet that closely follows the nutritional recommendations | 1. Labelling | 81 adolescents aged between 14-16 years old | Spain | Participants were randomly exposed to two experimental non-real food-choice conditions using multiple-traffic-light (MTL GDA) or monochrome nutritional labels M-GDA). Participants had to choose options from a closed menu for 5 days on the basis of the experimental front-of-pack labelling. For each meal, three food options with different nutritional compositions were given to the participants. The contents of total energy and fat, saturated fat, sugar and salt of the chosen options were calculated | No carry-over effect was observed between the experimental sequences on salt (t = 0·9, df = 75·3, P = 0·371). Participants using the MTL-GDA system chose significantly less salt; -0·4g, P < 0·001 than those using M-GDA labels. Differences between the most and the least healthy options represent 6·0 % less salt when the MTL-GDA nutritional label system was used | Sample was recruited from one school – cannot be generalized. Potential food choice was assessed and not real shopping or consumption |  |
| **Study ID** | **Study Design** | **Study Aim** | **Policy(ies) Analysed** | **Participants & sample size** | **Geographical Scope** | **Methods** | **Outcomes** | **Study Limitations** | **Comments** |
| Elfassy et al. (2015)^73^ | Cross-sectional | To examine the independent association between hypertension and frequency of use of the NF label for sodium information and determine whether frequent use in individuals with hypertension was associated with differences in mean sodium intake assessed through 24-hour urine samples | Labeling (use) | 1,656 adults | US | Data came from the New York City Community Health Survey Heart Follow-Up Study, a cross-sectional study conducted in 2010 in a representative sample of New York City adults. Participants were asked questions regarding frequency of checking the NF label and also had 24-hour urine samples collected to assess actual sodium intake | Daily sodium intake was not lower in those who reported frequent vs non-frequent use of the NF label for sodium information (3,084 mg vs 3,059 mg; P=0.924) | Results from this study of New York City participants may not be generalizable to the US population. It was possible that incomplete samples were included in the analysis. Focus on NF label use in those with hypertension irrespective of whether blood pressure was controlled. Individuals with hypertension that was controlled vs uncontrolled may have different NF label use and sodium consumption patterns that warrant further investigation |  |

**12F. Reformulation**

| **Study ID** | **Study Design** | **Study Aim** | **Policy(ies) Analysed** | **Participants & sample size** | **Geographical Scope** | **Methods** | **Outcomes** | **Study Limitations** | **Comments** |
| --- | --- | --- | --- | --- | --- | --- | --- | --- | --- |
| Chang et al. (2006)^78^ | Cluster randomised controlled trial | To examine the effects of potassium-enriched salt on CVD mortality and medical expenditures in elderly veterans | 1. Low sodium salt (reformulation) | 1982 elderly veterans | Taiwan | Five kitchens of a veteran retirement home were randomized into 2 groups (experimental or control) and veterans assigned to those kitchens were given either potassium-enriched salt (experimental group) or regular salt (control group) for 31 months. Information on death, health insurance claims, and dates that veterans moved in or out of the home was gathered | The incidence of CVD-related deaths was 13.1 per 1000 persons (27 deaths in 2057 person-years) and 20.5 per 1000 (66 deaths in 3218 person-years) for the experimental and control groups, respectively. A significant reduction in CVD mortality (age-adjusted hazard ratio: 0.59; 95% CI: 0.37, 0.95) was observed in the experimental group. Persons in the experimental group lived 0.3– 0.90 y longer |  |  |

**12**G. Taxes

| **Study ID** | **Study Design** | **Study Aim** | **Policy(ies) Analysed** | **Participants & sample size** | **Geographical Scope** | **Methods** | **Outcomes** | **Study Limitations** | **Comments** |
| --- | --- | --- | --- | --- | --- | --- | --- | --- | --- |
| Thow et al. (2014)^85^ | Systematic review | To assess the effect of food taxes and subsidies on consumption | 1. Tax on salty foods |  | US (with UK data) | Databases were searched for literature that focused on taxes and subsidies and assessed the effect on consumption | One study, using a modeled analysis of taxes in the United States (although limited by use of price elasticity data from the UK), found that a sodium excise tax that increased the price of salty foods by 40% would reduce sodium consumption by 6% | Only English papers were included. The lack of studies from low- and middle-income countries. The wide variety of targets of taxation that have been proposed and modeled add uncertainty to the conclusions that can be drawn regarding public health and policy measures. This study is also limited by its focus on assessments of fiscal policy interventions, which means that other, possibly relevant studies that focused only on price would have been excluded |  |
| **Study ID** | **Study Design** | **Study Aim** | **Policy(ies) Analysed** | **Participants & sample size** | **Geographical Scope** | **Methods** | **Outcomes** | **Study Limitations** | **Comments** |
| Niebylski et al. (2015)^86^ | Systematic review | To evaluate the evidence base to assess the effect of healthy food/beverage subsidies and unhealthy food/beverage taxation | 1. Taxation and subsidies |  | France and US | A comprehensive review was conducted by searching PubMed, Medline, and Google Scholar for peer-reviewed publications This review was performed in keeping with Preferred Reporting Items for Systematic Reviews and Meta-Analyses guidance | Kuchler et al. Modelled the effect of 1%, 10%, and 20% tax on chips/salty snacks on energy intake in United States. Predicted a 1% tax had no effect on consumption or body weight. Allais et al. Modelled the effect of a 1% value added tax on cheese/butter, sugar, and fat products along with ready-made meals in France. Household purchases were used to estimate energy intake. Predicted that these proposed taxes reduced saturated fat, cholesterol, sodium, and energy intake but suggests that a 1% is insufficient to have any positive health effect | Many of the studies were based on predictive modelling and price elasticity rather than real world interventions. There were limited interventions in remote communities and low- and middle-income countries. Given the 10-year window in their search criteria, there are multiple studies that were not identified in this review but that contribute significantly to the growing evidence base and deserve to be recognized. Difficulty in locating studies evaluating the effect on health outcomes over the long term. It is likely that this search missed several studies, as the restricted nature of the search terms used in databases excluded studies that could have been included in the review |  |

**12H. Multi-component interventions**

| **Study ID** | **Study Design** | **Study Aim** | **Policy(ies) Analysed** | **Participants & sample size** | **Geographical Scope** | **Methods** | **Outcomes** | **Study Limitations** | **Comments** |
| --- | --- | --- | --- | --- | --- | --- | --- | --- | --- |
| He et al. (2014)^88^ | Comprehensive analysis | To analyse the UK salt reduction programme with the aim to provide a step by step guidance of developing and implementing a national salt reduction strategies which other countries can follow | Media campaigns, voluntary reformulation and labelling |  | UK | The programme was evaluated by frequent surveys and repeated 24h urinary sodium at 3-5 year intervals | CASH proposed the UK salt reduction strategy in 2003 based on an average salt intake of 9.5 g per day as calculated from 24-h urinary sodium in the National Diet and Nutrition Survey.  An analysis of the Health Survey for England data showed a steady decline in salt use at the table since 1997, and this reduction was significantly greater after the introduction of the FSA’s salt reduction campaign in 2003. The percentage of adults who adds salt at the table decreased from 32.5% in 2003 to 23.2% in 2007.  *Primary outcomes:*  The average salt intake, as measured by 24-h urinary sodium excretion in a random sample of the adult population, was 9.5g per day in 2000/2001. Salt intake fell to 9.0g per day in 2005/2006, 8.6g per day in 2008 and fell further to 8.1g per day by 2011. As 24-h urinary sodium was not measured in 2003/2004 when the UK salt reduction programme started, it is assumed that salt intake was the same as that in 2000/2001. Therefore, from 2003/2004 to 2011, salt intake decreased by 1.4g per day (that is, 15%, P<0.05 for the downwards trend). In other words, there has been a steady fall in salt intake at a rate of ~2% per year since the introduction of the UK salt reduction strategy. The reduction occurred in both men and women, and in all age groups.  *Secondary outcomes:*  The 0.9-g per day reduction in salt intake achieved by 2008 led to E 6000 fewer CVD deaths per year. On the basis of the estimation of the National Institute for Health and Clinical Excellence, the further reduction of 0.5g per day achieved by 2011 would prevent E3000 additional CVD deaths amounting to a total of E9000 fewer CVD deaths per year. Further, there would also be E9000 non-fatal CVD events prevented per year |  |  |
| **Study ID** | **Study Design** | **Study Aim** | **Policy(ies) Analysed** | **Participants & sample size** | **Geographical Scope** | **Methods** | **Outcomes** | **Study Limitations** | **Comments** |
| Mozaffarian et al. (2012)^89^ | Systematic review | To systematically review and grade the current scientific evidence for effective population approaches to improve dietary habits, increase physical activity, and reduce tobacco use |  |  |  | First broad searches of online databases, including PubMed/MEDLINE, EconLit, AGRICOLA, ERIC, RePORT and the Social Science Citation Index. These searches were performed, followed by additional online searches, hand searches of citations, and use of expert contacts to identify systematic or narrative reviews in the scientific literature, as well as policy statements |  | For many interventions, there was limited or inconsistent evidence to evaluate the potential heterogeneity of effects, for example, depending on the population or level of intervention |  |
|  |  |  | Education |  | China | Community intervention | In the intervention neighbourhoods, mean sodium intake decreased by 22 and 11 mmol/day in men and women, respectively, compared with increases of 18 and 4 mmol/day, respectively, in the control neighbourhoods (P0.001 for men, P0.065 for women) |  | Already included separately |
|  |  |  |  |  | Finland |  | From the 1970s to the late 1990s, mean daily salt consumption in Finland declined from approximately 14.5 g in men (unknown in women) to approximately 11 g in men and 7 g in women; mean diastolic blood pressure declined by 5% in men and 13% in women; and mean total blood cholesterol declined by almost 20% |  |  |
| **Study ID** | **Study Design** | **Study Aim** | **Policy(ies) Analysed** | **Participants & sample size** | **Geographical Scope** | **Methods** | **Outcomes** | **Study Limitations** | **Comments** |
| Fattore et al. (2014)^43^ | Systematic review | To summarize and critically assess economic evaluation studies conducted on direct (e.g., counseling) or indirect (e.g., food labeling) interventions aimed at promoting voluntary dietary improvements through reduction of fat intake | 1. Dietary counselling and labelling |  |  | A systematic literature review was performed by searching 5 databases (PubMed, Ovid Medline, EconLit, Agricola, and Embase) using a combination of diet-related (fat, diet, intake, nutrition) and economic-related (cost-effectiveness, cost-benefit, cost-utility, health economics, economic evaluation) key words |  |  |  |
|  | Modelling study |  | 1. 1. Voluntary reformulation 2. 2. Mandatory reformulation 3. 3. Dietary advice |  | Australia |  | **Cobiac et al. (2010)**  610,000 DALYs averted (95%CI: 480,000–740,000) if everyone reduced their salt intake to recommended limits. Dietary advice: <0.5% disease burden (IHD & stroke cases) averted; Tick program: <1%; making Tick limits mandatory: 18% |  | Already included separately |
|  | Modelling study |  | A potential reduction in daily caloric intake of 100 to 500 kcal below current estimated energy requirements |  | US |  | **Dall et al. (2009)**  400 mg/d sodium intake reduction |  |  |
|  |  |  | A set of personal (e.g., individual treatment of SBP >160 mmHg) and non-personal (e.g., a mass media campaign for reducing consumption of salt) prevention strategies to reduce CVD |  | Vietnam |  | **Ha & Chisholm (2011)**  A health education program to reduce salt intake (VND 1,945,002 or USD 118 per DALY averted) & individual treatment of SBP >160 mmHg (VND 1,281,596 or USD 78 per DALY averted) are the most cost-effective measures |  | Already included separately |
|  |  |  | 1. Voluntary reformulation  2. Sodium tax |  | US |  | **Smith-Spangler (2010)**  (1) vs. (2): 1.25-mm Hg vs. 0.93-mm Hg decrease in mean SBP; 513,885 vs. 327,892 strokes averted; 480,358 vs. 306,137 MIs averted; 1.3 million vs. 840,113 years LE increase. Collaboration with industry: 2.1 million QALYs gained; USD 32.1 billion medical cost savings. Tax on sodium: 1.3 million QALYs gained; USD 22.4 billion medical cost savings |  | Already included separately |
| **Study ID** | **Study Design** | **Study Aim** | **Policy(ies) Analysed** | **Participants & sample size** | **Geographical Scope** | **Methods** | **Outcomes** | **Study Limitations** | **Comments** |
| He & MacGregor (2009)^90^ | Review | To provide an update on the current experience of worldwide salt reduction programmes, which have been successfully carried out in several countries and a reduction in salt intake has been achieved in these countries | 1. Salt initiatives including reformulation and health promotion campaigns |  | Japan, Finland, UK and 23 low and middle income countries | On the basis of the two search strategies developed earlier for meta-analyses on salt and BP in adults and children, they updated the search for electronic database—MEDLINE, EMBASE and the Cochrane Library. Furthermore, they reviewed the reference list of original and review articles to search for more studies | *Japan.* A recent randomized community-based intervention trial was carried out in 550 individuals in two rural villages in north-eastern Japan. The study demonstrated that dietary counselling for 1 year reduced salt intake by 2.3 g/day as measured by 24-h urinary sodium and this was associated with a decrease of 3.1mmHg in systolic BP.  The Japanese Government initiated a campaign to reduce salt intake. Over the following decade salt intake was reduced from an average of 13.5 to 12.1 g/day. However, in the north of Japan salt intake fell from 18 to 14 g/day. Paralleling this reduction in salt intake, there was a fall in BP both in adults and children, and an 80% reduction in stroke mortality despite large increases in population fat intake, cigarette smoking, alcohol consumption and an increase in body mass index.  *Finland.* Since the 1970s, Finland has had the aim to reduce salt intake in the whole population. This has been conducted through collaboration with the food industry to develop reduced-salt food products and raise the general awareness among consumers of the harmful effects of salt on health. Over the following 30 years, this resulted in a significant reduction in salt intake of the Finnish population, from an average of approximately 12 g/day in 1979 to less than 9 g/day in 2002 as measured by 24-h urinary sodium. This was accompanied by a fall of over 10mmHg in both systolic and diastolic BP, a pronounced decrease of 75–80% in both stroke and CHD mortality, and a remarkable increase of 5–6 years in life expectancy.  *23 low and middle income countries* Murray et al. showed that non-personal health interventions, including government action to stimulate a reduction in the salt content of processed foods, were cost-effective ways to limit CVD and could avert over 21 million DALYs (disability-adjusted life years) per year worldwide. Asaria et al. demonstrated that, over 10 years (from 2006 to 2015), a 15% reduction in mean population salt intake could avert 8.5 million cardiovascular deaths and a 20% reduction in smoking prevalence could avert 3.1 million cardiovascular deaths.  *UK.* In the UK, it was roughly estimated that 15% of the total 9.5 g of salt consumed (that is, 1.4 g) was added either at the table or in the cooking. Approximately 5% was naturally present in the food (that is, 0.6 g) and the rest, 80% (that is, 7.5 g), was not in the hands of the consumer and was added by the food industry either in processed, canteen, restaurant food, and so on  On a voluntary basis most processed foods bought in supermarkets have been reduced by 20 to 30% in the last 3 years in the UK |  |  |
| **Study ID** | **Study Design** | **Study Aim** | **Policy(ies) Analysed** | **Participants & sample size** | **Geographical Scope** | **Methods** | **Outcomes** | **Study Limitations** | **Comments** |
| Pietinen et al. (2010)^91^ | Before and after study | To describe the main actions in Finnish nutrition policy during the past decades | 1. 1. Education 2. 2. Voluntary reformulation 3. 3. Labelling |  | Finland |  | The National Nutrition Council recommended the lowering of the salt intake level in the population as far back as 1978, but no recommended level was given until 1981, after the first survey in Eastern Finland had shown that salt intake was about 13g in men and 11g in women. The recommended level was set at <9 g. Gradually the recommended level has been decreased, being 7–9 g in 1987, 7 g in 1994, 0.5 g/MJ (3–5 g NaCl) in 1998, and most recently, in 2005, 6g in women and 7g in men, the final long-term aim being 5g. Salt intake in the Finnish population has been monitored regularly in the FINRISK and FINDIET surveys, both using the 24 h urinary sodium and the calculated dietary sodium intake. Salt intake has decreased continuously to a level of about 9g in men and 7g in women in 2007. The decrease in salt intake has been the result of several actions: education of the public and health-care personnel, cooperation with the food industry, as well as national legislation concerning salt labelling. The national labelling decrees have been formulated by the Ministry of Trade and Commerce in close cooperation with the salt experts and food industry |  | No methods section |
| **Study ID** | **Study Design** | **Study Aim** | **Policy(ies) Analysed** | **Participants & sample size** | **Geographical Scope** | **Methods** | **Outcomes** | **Study Limitations** | **Comments** |
| Wang et al. (2011)^92^ | Literature review of modelling studies | To summarize cost-effectiveness evidence on selected interventions to reduce sodium intake that would be intended as population-wide approaches to control hypertension |  |  |  |  |  | Publication bias may be present. It may be hard to generalize the health economic data across countries. All US studies used simulation models under hypothetical scenarios rather than data on the actual intervention to evaluate the approaches to sodium reduction |  |
|  |  |  | Reformulation and a sodium tax |  | US |  | **Smith-Spangler et al.**  For US adults aged 40–85 years, collaboration with industry that decreased mean intake of sodium by 9.5% was estimated to avert 513 885 strokes and 480 358 myocardial infarctions over their lifetimes and to save US$ 32.1 billion in annual medical costs. Over the same period, a tax on sodium that decreased the population’s intake of sodium by 6% was projected to save US$ 22.4 billion in such costs |  | Already included separately |
|  |  |  |  |  | US |  | Palar and Sturm found that reducing average sodium intake to 2300 mg/day might eliminate 11 million cases of hypertension, save US$ 18 billion in healthcare costs, and gain 312 000 QALYs valued at US$ 32 billion on an annual basis |  | Unable to get original paper |
| **Study ID** | **Study Design** | **Study Aim** | **Policy(ies) Analysed** | **Participants & sample size** | **Geographical Scope** | **Methods** | **Outcomes** | **Study Limitations** | **Comments** |
| Webster et al. (2011)^93^ | Review | To provide an overview of national salt reduction initiatives around the world, describe core characteristics and develop a framework for future strategy development | 1. Salt reduction initiatives |  | Europe, Americas and Western Pacific region | National strategies were identified from existing reviews and from searches of the literature and relevant websites. Standardized information was extracted about governance and strategy development, baseline assessments and monitoring and implementation | Thirty-two national salt reduction initiatives were identified, 19 in Europe, six in the Americas and seven in the Western Pacific Region. There were no salt reduction strategies identified in Africa.  *Finland.* Finland commenced efforts to reduce salt in 1978 and by 2002 had demonstrated a 3 g reduction in average population salt intake (from 12 to 9 g/person per day). During the same period there was a corresponding 60% fall in coronary heart disease and stroke mortality. Key characteristics include: strong leadership through the Finnish National Nutrition Council with clear population targets; regular monitoring of population salt consumption using 24-h urinary assessments and dietary survey data; mass media campaigns and education of healthcare personnel; extensive stakeholder and community involvement; voluntary cooperation with the food industry to reduce salt including the use of Pan salt (a reduced sodium salt substitute); and mandatory warning labels for foods high in salt which drove many high salt foods from the supermarket shelves.  *UK.* The UK Food Standards Agency (FSA) started working with the food industry in 2003 and launched its consumer education campaign in 2005. By 2008 the UK had achieved an average 0.9 g/person per day reduction in daily salt consumption, which is predicted to be saving some 6000 lives a year.  *Ireland.* Ireland published its scientific report on salt in 2005 and shortly thereafter the Irish Food Standards Agency (FSAI) initiated a salt reduction initiative. The initial goals for selected food products were achieved by 2008 including reducing the salt in breads by 10%, sauces by 15% and soups by 10%. More challenging reformulation targets have since been set  *France.* The French Food Safety Authority recommended a reduction in population salt consumption in 2000 and has since reported a decline in intake provided by foods from 8.1 to 7.7 g/day in the overall adult population. The French salt industry has also reported a 15% reduction in sales of salt to food manufacturers between 2001 and 2006 and a parallel 5% reduction in the sales of household salt. Key characteristics of the French approach have been a focus on bread (although bread is one of the few products in which salt levels have not declined) and salt messages disseminated as part of broader nutrition campaigns.  *Japan.* The Japanese Government initiated a campaign to reduce salt intake in the 1960s through a sustained public education programme. Prior to that deaths from stroke in Japan were among the highest in the world, and it became apparent that certain regions, particularly the north, were consuming as much as 18 g/day of salt. Over the following decade average salt intake was reduced from 13.5 to 12.1 g/day with a parallel fall in blood pressure in adults and children, and an 80% reduction in stroke mortality despite large adverse changes in a range of other cardiovascular risk factors. In the absence of any sustained government programme there are indications that salt intakes are once again gradually rising in Japan | Webster et al. (2011) | Review |
| **Study ID** | **Study Design** | **Study Aim** | **Policy(ies) Analysed** | **Participants & sample size** | **Geographical Scope** | **Methods** | **Outcomes** | **Study Limitations** | **Comments** |
| Wang & Bowman (2013)^94^ | Literature review | To summarize recent economic analyses of interventions to reduce sodium intake |  |  |  | They searched six databases for potentially relevant articles published in English during the period from January 2011 to March 2013 |  |  |  |
|  |  |  | (1) reducing the sodium content of all foods in the settings of child care, senior meal programs, cafeterias, and mobile trucks; and (2) reducing sodium content by labeling foods and by promoting, subsidizing, and providing lows odium food options in hospitals and in county government cafeterias |  | US |  | If the sodium-reduction strategies were implemented, adults in the county would reduce their intake of sodium by 233 mg per day, on average, in 2010. This would correspond to an average decrease of 0.71 mmHg in SBP among adults with hypertension, 388 fewer cases of uncontrolled hypertension, and a decrease per year of $629,724 in direct health care costs |  |  |
|  |  |  |  |  | UK |  | They found that legislation or other measures to reduce the intake of salt by 3 g per person per day (in a population where the current mean intake was about 8.5 g per person per day) would reduce the mean population SBP by approximately 2.5 mmHg, prevent about 30,000 cardiovascular events and approximately 4,450 deaths, and produce discounted savings overall of approximately £347 million (about $684 million) over a decade, which would be equivalent to annual savings of approximately £40 million |  |  |
| **Study ID** | **Study Design** | **Study Aim** | **Policy(ies) Analysed** | **Participants & sample size** | **Geographical Scope** | **Methods** | **Outcomes** | **Study Limitations** | **Comments** |
| He et al. (2014)^95^ | Cross-sectional study | To determine the relationship between the reduction in salt intake that occurred in England, and blood pressure (BP), as well as mortality from stroke and ischemic heart disease (IHD) | Salt reduction interventions | 2003 N=9183,  2006 N=8762, 2008 N=8974 and 2011 N=4753, aged  ≥16 years | England | Data from the Health Survey for England for 2003, 2006, 2008 and 2011 was used for analysis  Comparisons among the 4 years were made by one-way analysis of variance for continuous variables and by χ2 test for categorical variables | *Primary outcomes:*  The average salt intake, as measured by 24 h urinary sodium excretion in a random sample of the adult population, was 9.5±0.2 g/day in 2003. Salt intake fell to 9.0±0.4 g/day in 2005/2006, 8.64±0.2 g/day in 2008 and further to 8.1±0.2 g/day by 2011. Therefore, from 2003 to 2011, salt intake decreased by 1.4 g/day (i.e. 15%, p<0.05 for the downward trend).  *Secondary outcomes:*  From 2003 to 2011, stroke mortality decreased from 128/1 000 000 to 82/1 000 000 (36% reduction, p<0.001) and IHD mortality decreased from 423/1 000 000 to 272/1 000 000 (36% reduction, p<0.001). In individuals who were not on any antihypertensive medications or other medications that might affect BP, there was a fall in BP of 1.9±0.34/1.0±0.25 mm Hg (p<0.001 for systolic and diastolic BP) from 2003 to 2011 after adjusting for age, sex, ethnic group, education level, household income, alcohol consumption, fruit and vegetable intake and BMI | Potential ecological bias. They could not exclude the potential confounding effect of some variables which were not measured, such as physical activity levels which were recorded in 2003, but not in the 2011 survey. The trend in 24h urinary sodium was taken from data for England, Great Britain or the UK as the original report did not separate results by countries. Potentially the higher intake in Scotland and lower intake in Wales might balance each other out | This programme looked at reformulation and health promotion campaigns. They suggested that reformulation contributed most to the reduction in dietary salt intake |
| **Study ID** | **Study Design** | **Study Aim** | **Policy(ies) Analysed** | **Participants & sample size** | **Geographical Scope** | **Methods** | **Outcomes** | **Study Limitations** | **Comments** |
| Enkhtungalag et al. (2015)^96^ | Before and after study | To reduce salt intake of employees of three of the main food producing factories | 1. Nutrition education and provision of reduced salt foods |  | Mongolia | Employees of three food producing factories received training on the negative impact of salt and how to consume a healthy diet and were provided reduced salt meals. Salt intake was measured using 24h urinary excretion and a questionnaire | Results showed that salt intake reduced from 11.48 (7.32) g per day in 2011 to 8.65 (4.26) g per day in 2013. Number of respondents who didn’t know which foods were high in salt declined from 18.5% (95% CI, 14.0-23.9) to 2.9% (95% CI, 1.1-7.4) during the same period | Time limited |  |
| **Study ID** | **Study Design** | **Study Aim** | **Policy(ies) Analysed** | **Participants & sample size** | **Geographical Scope** | **Methods** | **Outcomes** | **Study Limitations** | **Comments** |
| Trieu et al. (2015)^24^ | Systematic review | To quantify progress with the initiation of salt reduction strategies around the world in the context of the global target to reduce population salt intake by 30% by 2025 | 1. Reformulation, education, mass media campaigns and labelling |  | 75 countries by WHO region | A systematic review of the published and grey literature was supplemented by questionnaires sent to country program leaders. Core characteristics of strategies were extracted and categorised according to a pre-defined framework |  | Much of the information was obtained from country questionnaires which relied on the knowledge and potentially subjective opinion of one country representative, usually a government agency. Methodological rigor behind some of the reports is unknown  Focus on national and not local strategies |  |
| Du et al. (2014) | Open cohort study |  | 1. Labelling & media campaign |  | China | Dietary survey (1991 to 2009) | Salt intake reduced from 16.8g to 12g/day (28%) |  | Already included separately |
|  |  |  | 1. Voluntary sodium targets, media campaign and voluntary labelling |  | Denmark | Spot urine measurement tool (2006-2010) | Salt intake reduced from 10.7 to 9.9g/day in men and 7.5g to 7.0g/day in women (7%) |  |  |
| Pietinen et al. (2010) & Laatikanen et al. (2006) | Cross-sectional study |  | Voluntary sodium targets, media campaigns, labelling and education and food procurement policy with sodium standards in schools, work places and hospitals |  | Finland | Dietary survey and 24h urine (1979-2007) | Salt intake reduced from 13g to 8.3g/day in men and 11g to 7.0g/day in women (36%) |  |  |
| European commission (2008) | Report |  | Voluntary sodium targets, media campaigns and food procurement policy with sodium standards in schools |  | France | Dietary survey (1999-2007) | Salt intake reduced from 8.1g to 7.7g/day (4.9%) |  |  |
| WHO (2013) | Report |  | Media campaigns and meetings with the industry to discuss salt reformulation |  | Iceland | Dietary survey (20002-2010) | Salt intake reduced from 8.4g to 7.9g/day (6%) |  |  |
| Walton (2013) |  |  | Sodium targets for foods, media campaigns, labelling and education in schools |  | Ireland | Dietary survey (2001-2011) | Salt intake reduced from 8.1g to 7g/day (13.6%) |  | PPT slides |
|  |  |  | 1. Industry meetings to reformulate and media campaigns |  | Japan | Dietary survey (1997-2012) | Salt intake reduced from 13.5 to 10.4g/day (23%) |  |  |
|  |  |  | Voluntary sodium targets for foods, media campaigns, labelling – both voluntary % of daily intake and mandatory traffic light, education in schools and a food procurement policy with sodium standards at the workplace |  | Korea | Dietary survey (2005-2012) | Salt intake reduced from 13.4g to 11.6g/day (13.6%) |  |  |
| National Food and Veterinary Risk Assessment Institute | Report |  | Sodium content targets for foods, media campaigns, voluntary labelling and education and food procurement policy with sodium standards in schools |  | Lithuania | Dietary survey (1997-2007) | Salt intake reduced from 10.8g to 8.8g/day (18.6%) |  | No title page or date on the report |
|  |  |  | Voluntary sodium content targets for foods, media campaigns and education and food procurement policy with sodium standards in schools |  | Slovenia | 24h urine (2007-2012) | Salt intake reduced from 12.4g to 11.3g/day (8.9%) |  |  |
| WHO (2013) |  |  | Voluntary sodium content targets for foods, media campaigns, and education and procurement policy with sodium standards in schools, workplaces and hospitals |  | Turkey | 24h urine (2008-2012) | Salt intake reduced from 18.0g to 15g/day (16.7%) |  | Webpage |
| Sadler et al. (2011) |  |  | 1. Voluntary sodium content targets for foods, media campaigns, voluntary traffic light labelling, education and procurement policy with sodium standards in schools |  | UK | 24h urine (2001-2011) | Salt intake reduced from 9.5g to 8.1g/day (14.7%) |  |  |
| **Study ID** | **Study Design** | **Study Aim** | **Policy(ies) Analysed** | **Participants & sample size** | **Geographical Scope** | **Methods** | **Outcomes** | **Study Limitations** | **Comments** |
| Luft et al. (1997)^97^ | Review | To discuss the approaches used in a community-wide salt-reduction project |  |  |  |  |  |  | No methods section |
|  |  | To evaluate the feasibility and effects of salt reduction in the general population | 1. Reformulation, health promotion | 17.000 hypertensive patients | Finland | Health education was conducted throughout the entire 3-y study and local press and radio were also used. About 1 7 000 hypertensive patients lived in this area and were mailed written guidelines on salt reduction. Health personnel, including doctors, public health nurses, home economics teachers, and other health care personnel working in schools and hospitals were contacted and trained by nutritionists employed for the project. Home visits were also conducted. The local food industry was contacted | After 3 y salt intake had not changed significantly. In women, mean sodium excretion decreased somewhat, with a significant decrease in the ratio of sodium to potassium (Na: K).  *Hypertensive subjects*  Sodium (mmol/day)  **Men**   - 1979: 240 ± 92 - 1982: 239 ± 95   **Women**   - 1979: 181 ± 81 - 1982: 174 ± 71   *Normotensive subjects*  Sodium (mmol/day)  **Men**   - 1979: 215 ± 83 - 1982: 213 ± 83   **Women**   - 1979: 170 ± 66 - 1982: 158 ± 63 |  |  |
|  |  | To develop dietary salt restriction as an adjunctive treatment for hypertension | 1. Dietary counselling |  | US | Families received instructions and counselling for restricting their sodium intake to <60mmol/day. A 2-4 week stabilization period was included followed by a 3 months intervention. 24h collections were used to determine sodium intake | Women reduced their sodium excretion from 130 ± 7 to 62 ± 4 mmol/d (ẋ± SD) and men reduced their sodium excretion from 179 ± 14 to 81 ± 5 mmol/d |  |  |
|  | Placebo controlled randomized clinical trial | To examine the efficacy and acceptability of diet alone or in combination with therapy in the treatment of hypertension | 1. Education |  | US | The TAIM educational program consisted of 10 weekly group sessions and 2 individual counseling sessions. A 6-wk follow-up schedule included a minimum of two subsequent follow-up counseling sessions. Specific goals and food diaries were used to facilitate self-monitoring. A food demonstration was included in each of the 10 group sessions | Daily sodium excretion was reduced from 138 to 1 12 mmol/d. Analysis of 3-d food records indicated that sodium intake decreased from 141 to 86 mmol/d. At 6 months, reported (recall) dietary sodium intake was significantly lower than the intake measured by 24-h urine excretion |  |  |
|  |  |  | 1. Nutrition education | 2182 men and women |  | Three lifestyle interventions (weight reduction, sodium reduction and stress management) were compared with unmasked non-intervention controls. >18 months | Urinary sodium (mmol/day)  *Intervention*   - Baseline: 155 ± 59 - Change: -55 ± 76   *Control*   - Baseline: 156 ± 60 - Change: -11 ± 76 |  |  |
| **Study ID** | **Study Design** | **Study Aim** | **Policy(ies) Analysed** | **Participants & sample size** | **Geographical Scope** | **Methods** | **Outcomes** | **Study Limitations** | **Comments** |
| Mohan et al. (2009)^98^ | Analysis | To review the evidence related to dietary sodium and health in the context of the Ottawa Charter for Health Promotion, an internationally adopted framework for health organizations to advocate health promotion | 1. Reformulation  2. Labelling  3. Health promotion campaign |  | Finland, UK and Taiwan | We searched MEDLINE (January 1980 to December 2008) for peer reviewed literature on sodium and sodium-reduction strategies. We searched the identified articles for additional studies of interest. To obtain grey literature and reports, we screened Google Scholar and the websites of the World Health Organization (WHO) and other relevant health organizations | *Finland.* Finland has had a population based policy for sodium reduction anchored on partnership and regulation of the food industry and consumer education via mass media. This has resulted in a 40% decrease in population sodium consumption, a de crease of more than 10 mm Hg in blood pressure and a reduction of 70% in mortality from stroke and coronary artery disease.  *UK.* Simple and effective consumer-friendly labelling indicating sodium content in processed foods by use of a colour system (red for high salt content, amber for medium salt and green for low salt) is being implemented in several UK food chains. These and other concurrent efforts in the UK have resulted in a reduction of the average population intake of sodium from 3800 mg in 2004 to 3440 mg in 2008.  *Taiwan.* A cluster randomized trial among elderly Taiwanese people in which regular salt was substituted with a low-sodium salt markedly reduced cardiovascular events. Salt substitution may be a cost-effective strategy in developing countries where the principal source of sodium in the diet is salt added during domestic cooking |  |  |
| **Study ID** | **Study Design** | **Study Aim** | **Policy(ies) Analysed** | **Participants & sample size** | **Geographical Scope** | **Methods** | **Outcomes** | **Study Limitations** | **Comments** |
| He & MacGregor et al. (2010)^99^ | Comprehensive review | To provide an update on the current salt reduction programs that have been successfully carried out in several countries | 1. Reformulation, mass media campaigns and labelling |  | Japan, Finland and UK |  | *Japan.* The Japanese Government initiated a campaign to reduce salt intake. Over the following decade, the national salt intake was reduced from an average of 13.5 to 12.1 g/day, and in the north, salt intake fell from 18 to 14 g/day. Paralleling this reduction in salt intake, there was an 80% reduction in stroke mortality despite large increases in fat intake, cigarette smoking, alcohol consumption, and obesity.  *Finland.* Finland was one of the first countries to initiate a systematic approach to decrease salt intake in the population, in the late 1970s, through mass-media campaigns, cooperation with the food industry, and implementing salt labelling legislation. These different measures have resulted in a significant reduction in salt intake of the Finnish population, from an average of approximately 12 g/day in 1979 to less than 9 g/day in 2002. This was accompanied by a fall of more than 10 mm Hg in both systolic and diastolic BP, a pronounced decrease of 75% to 80% in both stroke and CHD mortality, and a remarkable increase of 5 to 6 years in life expectancy.  *UK.* A strategy to reduce population salt intake was developed based on the United Kingdom's average salt intake of 9.5 g/day as measured by 24-hour urinary sodium. The United Kingdom salt reduction strategy started in 2003/2004 and salt intake has already fallen from 9.5 to 8.6 g/day by May 2008 |  | Methods not reported |
| **Study ID** | **Study Design** | **Study Aim** | **Policy(ies) Analysed** | **Participants & sample size** | **Geographical Scope** | **Methods** | **Outcomes** | **Study Limitations** | **Comments** |
| Wyness et al. (2012)^100^ | Literature review | To describe the UK Food Standards Agency's (FSA) salt reduction programme undertaken between 2003 and 2010 and to discuss its effectiveness | 1. Voluntary reformulation and health promotion campaign + labelling |  | UK | Relevant scientific papers, campaign materials and evaluations and consultation responses to the FSA's salt reduction programme were used | In 2000–2001 salt intake in adults was estimated to be 9.5 g. By 2005–2006, average intake among adults had fallen by 0.5 g/d to 9.0 g/d. Urinary Na levels were assessed again in 2008, and the average adult population salt intake was estimated to be 8.6g/d. The 0.9 g reduction in UK salt intakes (since 2001) is assumed to be the direct result of efforts across the manufacturing, retail and (more recently) food service sectors to reformulate products, coupled with changes in consumers’ dietary practices |  |  |

**12I. Salt intake outcomes with interventions detailed in other publications**

| **Study ID** | **Study Design** | **Study Aim** | **Policy(ies) Analysed** | **Participants & sample size** | **Geographical Scope** | **Methods** | **Outcomes** | **Study Limitations** | **Comments** |
| --- | --- | --- | --- | --- | --- | --- | --- | --- | --- |
| Laatikainen et al. (2006)^25^ | Cross-sectional population surveys | To present trends in urinary sodium and potassium excretion from 1979 to 2002 | Reformulation, labelling and mass media campaigns | 4648 adults aged 24-64 years old | Finland | Surveys were carried out in Finland in 1979, 1982, 1987 and 2002 in four geographical areas: North Karelia, the Kuopio area, Southwestern Finland and the Helsinki area. A 24-h urinary collection was carried out in connection with population risk factor surveys. Urinary sodium and potassium concentrations were analyzed in the same laboratory throughout, using a flame photometer in 1979, 1982 and 1987 and an ion-selective electrode in 2002 | Between 1979 and 2002 urinary sodium excretion in Finland decreased from over 220 to less than 170 mmol/day among men and from nearly 180 to less than 130 mmol/day among women. In 2002, the salt intake in grams per 24 h calculated from sodium excretion was about 9 .5 g among men in North Karelia and southwestern Finland and 8.6 g in the Helsinki area. Among women the corresponding values were 7.4 g in North Karelia and southwestern Finland and 6.9 g in the Helsinki area. The age and area adjusted annual average reduction in salt intake between 1979 and 2002 was 0.14 g in men and 0.11 g in women. In 1979 the most educated North Karelian men had lower salt intake compared to the least educated being 11.4 g (95% CI, 10.5–12.3) in the highest education tertile and 13.1 g (95% CI, 12.1–14.1) in the lowest tertile. Respectively, in 2002, the salt intake in southwestern Finland among women in the highest education tertile was 6.7 g (95% CI, 6.1–7.3) compared to 8.1 g (95% CI, 7.3–8.9) in the lowest tertile |  | Linked to other papers describing timing of interventions |
| **Study ID** | **Study Design** | **Study Aim** | **Policy(ies) Analysed** | **Participants & sample size** | **Geographical Scope** | **Methods** | **Outcomes** | **Study Limitations** | **Comments** |
| Otsuka et al. (2011)^101^ | Longitudinal study | To describe salt intake for 8 years according to age groups, and examines whether salt intake changes over time in community-dwelling middle-aged and elderly Japanese subjects |  | 544 men aged 40-70 years old and 512 women aged 70-79 years old | Japan | Data were collected as part of the National Institute for Longevity Sciences Longitudinal Study of Aging. Participants participated in and completed all nutrition surveys from the first (1997-2000) to fifth (2006-2008) study waves. Each study wave was conducted for 2 years; in individuals, the entire follow-up period was 8 years. Salt and energy intake were calculated from 3-day diet records with photographs. The mixed-effects regression model was used for analysis of repeated measures of salt intake | Average daily salt intake was 12.8± 3.3 g/day in men and 10.6± 2.5 g/day in women at first participation. There was no consistent change in salt intake in women across each age group or study wave. Estimated linear changes in salt intake for 8 years by age group were estimated according to the slope of salt intake. Among men, the slope of salt intake (grams/day) per study wave declined by age group with values of 0.15 g among 40- to 49-year-olds (P=0.057), 0.19 g among 50- to 59-year-olds (P=0.007), 0.32 g among 60- to 69-year-olds (P<0.0001), and 0.29 g among 70- to 79-yearolds (P=0.017), respectively. Because each study wave was conducted for 2 years, mean salt intakes in men decreased 0.08 g/year among 40- to 49-year-olds, 0.09g/year among 50- to 59-year-olds, 0.16 g/year among 60-to 69-year-olds, and 0.14 g/year among 70- to 79-year olds. For women, the slope of salt intake (grams/day) per study wave among 70- to79-year-olds showed a decline toward a lower intake of 0.17 g or 0.08 g/year, although this value was marginally significant (P=0.098). Estimated linear changes in salt intake by age group after adjusting for energy intake are shown in the Figure. In men, a 0.14-g per study wave decline was observed among 60- to 69-year-olds, which represented a decline of 0.07 g/year (=P0.049). In women, a 0.13-g per study wave increase was observed among 50- to 59-year-olds, which represented an increase of 0.06 g/year (P=0.015) | Eight years was too short a period to investigate trends in salt intake. Adjustment for a wide range of potential confounding variables, including a history of hypertension, medications, or dietary interventions, was not performed | This paper was linked to other papers about Japanese population that describe salt interventions in a certain time span to estimate effect measures |
| **Study ID** | **Study Design** | **Study Aim** | **Policy(ies) Analysed** | **Participants & sample size** | **Geographical Scope** | **Methods** | **Outcomes** | **Study Limitations** | **Comments** |
| Du et al. (2014)^102^ | Ongoing open cohort study | To analyse the patterns and trends of dietary sodium intake, potassium intake and the Na/K ratio and their relations with incident hypertension in China | Labelling & media campaign | 16,869 adults aged 20–60 y | China | The China Health and Nutrition Survey cohort includes 16,869 adults aged 20–60 y from 1991 to 2009. Three consecutive 24-h dietary recalls and condiment and food weights provided detailed dietary data. Multinomial logistic regression models determined trends and patterns of sodium and potassium intake and the Na/K ratio. Models for survival-time data estimated the hazard of incident hypertension | Sodium intake decreased from 6.6 g/d in 1991 to 4.7 g/d in 2009 | Past studies possibly overestimated salt intake, whereas this study possibly underestimated it. During periods of heavy salting and preservation, the excessive discarded salt solution was not measured. The lack of urine collection and analysis of 24-h urinary sodium excretion | Linked to Trieu et al. (2015) |
| **Study ID** | **Study Design** | **Study Aim** | **Policy(ies) Analysed** | **Participants & sample size** | **Geographical Scope** | **Methods** | **Outcomes** | **Study Limitations** | **Comments** |
| Miura et al. (2000)^103^ | Report | To introduce the target level of dietary salt reduction and its scientific evidence, present status of salt consumption in Japan, salt-reducing measures/guidance methods in individuals and population strategies to reduce salt intake |  |  | Japan |  | The National Health and Nutrition Survey in 2010 reported that the mean salt intake in adults was 10.6 g per day (men: 11.4 g per day and women: 9.8 g per day). There was an ~4 g decrease in comparison with that in 1972 (14.5 g), when salt intake was investigated for the first time in the National Nutrition Survey. According to surveys in the 1950s, the mean salt intake was 27 g per day in the Tohoku district and 17 g per day in the Kinki district; therefore, it may have more markedly decreased during the past 60 years |  | Link to other papers describing timing of interventions |

# Table 13. All included modelling papers

| **Study ID** | **Study Design** | **Study Aim** | **Policy(ies) Analysed** | **Participants & sample size** | **Geographical Scope** | **Methods** | **Outcomes** | **Study Limitations** | **Sources referenced & Comments** |
| --- | --- | --- | --- | --- | --- | --- | --- | --- | --- |
| Cobiac et al. (2010)^54^ | Modelling study | To evaluate population health benefits and cost-effectiveness of interventions for reducing salt in the diet | 1. 1. Voluntary reformulation 2. 2. Mandatory reformulation 3. 3. Dietary advice |  | Australia | Four salt interventions ((a) the current practice ‘Tick’ programme, which provides incentives for voluntary changes by food manufacturers; (b) legislation and enforcement to make Tick salt limits mandatory for food manufacturers; (c) dietary advice for everyone at increased risk of cardiovascular disease (systolic blood pressure >115 mmHg); and (d) dietary advice for those at high risk (systolic blood pressure >140 mmHg)) are evaluated for effectiveness and cost-effectiveness | *Secondary outcomes:*  A total of 610 000 DALY (95% uncertainty interval 480 000 to 740 000 DALY) could be averted over the lifetime of the population if everyone reduced their salt intake to recommended limits. Providing dietary advice to the population with hypertension (systolic blood pressure >140 mmHg) or everyone at risk (systolic blood pressure >115 mmHg) might avert less than 0.5% (1,700 – 2,600 DALYs) of this disease burden, and the Tick programme (as modelled for breads, margarines and cereals) less than 1% (5,300 DALYs). Making Tick limits mandatory for all bread, margarine and cereal products, however, could avert 18% of the disease burden (110,000 DALYs). That would be 20 times the health gain potentially achieved with the voluntary approach. Both the voluntary Tick Reformulation programme and mandatory salt reduction had a 100% probability of being dominant (i.e., cost saving to the health sector) under all modelled scenarios of discounting, costing and reversal of risk. In contrast, there was zero probability of dietary advice being cost-saving or cost-effective against the A$50 000 per DALY threshold, even when targeted at the population most at risk |  | **Reformulation**: *Young L, Swinburn B. Impact of the Pick the Tick food information programme on the salt content of food in New Zealand. Health Promot Int 2002;17:13e19*  *McLennan W, Podger A. National nutrition survey: foods eaten, Australia, 1995. Australia: Commonwealth of Australia: Australian Bureau of Statistics, 1999*  **Dietary advice**: *Hooper L, Bartlett C, Davey Smith G, et al. Advice to reduce dietary salt for prevention of cardiovascular disease. Cochrane Database Syst Rev 2004;(1):CD003656* |
| **Study ID** | **Study Design** | **Study Aim** | **Policy(ies) Analysed** | **Participants & sample size** | **Geographical Scope** | **Methods** | **Outcomes** | **Study Limitations** | **Sources referenced & Comments** |
| Cobiac et al. (2012)^55^ | Modelling study | To evaluate the optimal mix of lifestyle, pharmaceutical and population-wide  interventions  for  primary  prevention of  cardiovascular disease | 1. 1. Mandatory reformulation in bread, margarines and cereals 2. 2. Community heart health programme 3. 3. Dietary advice | Men and women aged 35 to 84 years old who have never experienced heart disease or stroke event | Australia | In a discrete time Markov model they simulated IHD and stroke outcomes and cost impacts of interventions aimed at the Australian population | *Secondary outcomes*  Mandating more moderate use of salt in breads, margarines and cereals is easily the most effective and cost-effective strategy for primary prevention of CVD; it produces the biggest improvements in population health (80,000 DALYs), and can save money for the health sector (dominant). No other interventions represent good value for money. A community heart health program can achieve only small improvements in population health (3,000 DALYs; $44,000)). These interventions, although cost-effective if implemented as isolated strategies, are not cost-effective if other more cost-effective strategies (mandatory salt reduction) are provided first. The behaviour change interventions such as dietary advice can achieve only small improvements in population health (180-370 DALYs) and are least cost-effective of all the primary prevention strategies ($ 1,000,000 to $1,400,000) | They assumed that the effects (and costs) of these interventions will be sustained for those who continue to participate, but further evidence is needed to clarify the sustainability of different intervention approaches | **Voluntary reformulation = current practice**  mgNa/day men 0.50 (0.03)  mgNa/day women 0.34 (0.02)  *Young L, Swinburn B (2002) Impact of the Pick the Tick food information programme on the salt content of food in New Zealand. Health Promotion International 17: 13–19.*  *BS (1995) National Nutrition Survey: Foods Eaten, Australia, 1995. Canberra: Australian Bureau of Statistics*  **Mandatory reformulation**: mgNa/day men 10.6 (0.74)  mgNa/day women 7.3 (0.53)  *Young L, Swinburn B (2002) Impact of the Pick the Tick food information programme on the salt content of food in New Zealand. Health Promotion International 17: 13–19.*  *Asaria P, Chisholm D, Mathers C, Ezzati M, Beaglehole R (2007) Chronic disease prevention: health effects and financial costs of strategies to reduce salt intake and control tobacco use. Lancet 370: 2044–2053*  *BS (1995) National Nutrition Survey: Foods Eaten, Australia, 1995. Canberra: Australian Bureau of Statistics*  *WHO (2009) Tables of Costs and Prices used in WHO-CHOICE Analysis. Choosing Interventions that are Cost Effective (WHO-CHOICE): World Health Organisation*  **Community heart health programme**  *Pennant M, Davenport C, Bayliss S, Greenheld W, Marshall T, et al. (2010) Community programs for the prevention of cardiovascular disease: a systematic review Am J Epidemiol 172: 501–516.*  *Ronckers ET, Groot W, Steenbakkers M, Ruland E, Ament A (2006) Costs of the ‘Hartslag Limburg’ community heart health intervention. BMC Public Health 6*  **Dietary advice**: Systolic BP Total cholesterol 1.6% (0.4%) 3.1% (1.2%)  *Department of Veterens’ Affairs (2008) Dietitians schedule of fees. Australian Government*  *Brunner E, Rees K, Ward K, Burke M, Thorogood M (2007) Dietary advice for reducing cardiovascular risk. Cochrane Database of Systematic Reviews Issue 4* |
| **Study ID** | **Study Design** | **Study Aim** | **Policy(ies) Analysed** | **Participants & sample size** | **Geographical Scope** | **Methods** | **Outcomes** | **Study Limitations** | **Sources referenced & Comments** |
| Nghiem et al. (2015)^56^ | Modelling study | To compare the impact of eight sodium reduction interventions, including feasible and more theoretical ones, to assist prioritization | 1. 1. Dietary counselling 2. 2. Labelling 3. 3. Mandatory 3G reformulation 4. 4. Mandatory all reformulation 5. 5. UK package (multiple policies) 6. 6. Mass media campaign 7. 7. Tax | New Zealand population aged 35 years and older (2.3 million people) | New Zealand | Epidemiological modelling and cost-utility analysis were performed using a Markov macro-simulation model to study CVD interventions | *Secondary outcomes*  This QALY benefit was followed in descending order by that from a: (i) Salt Tax (195,000 QALYs gained); (ii) mandatory 25% reduction of sodium levels in processed food (“MandatoryAll”), (110,000); (iii) the package of interventions performed in the UK (85,100); (iv) mandatory 25% reduction in sodium levels in bread, processed meats and sauces (“Mandatory-3G”), (61,700); (v) Media Campaign as per the UK one (25,200); (vi) the voluntary Endorsement Label Programme as currently used in New Zealand (7900); and (vii) Dietary Counselling as currently used in New Zealand (200 QALYs gained). | Model structure and indeed uncertainty estimates did not capture uncertainty arising from “model structure uncertainty”. Limitations around input parameters. Unknowns in public and industry responses like compensatory behaviours in response to perceived reduced saltiness of processed foods. Taking a health system perspective and ignoring the economic benefits of preventing premature deaths in workers | **Dietary counselling:** For the per hour impact of counselling: 7.6 mmol/d reduction (with uncertainty based on the initial trials in the Cochrane review. SD=0.8 mmol/d). Normally distributed. Total amount of counselling in NZ: 4600 h/year (SD=920). Gamma distribution.  *Ministry of Health (2013) Ways and Means: A report on methodology from the New Zealand Burden of Disease, Injury and Risk Study, 2006–2016. Wellington: Ministry of Health.* [*http://www.health.govt.nz/publication/ways-and-means-report-methodology-new-zealand-burden-disease-injury-and-risk-study-2006-2016*](http://www.health.govt.nz/publication/ways-and-means-report-methodology-new-zealand-burden-disease-injury-and-risk-study-2006-2016)  **Labelling**:  Effect size: 1.7 mmol/d reduction overall (38 mg/d) with SD at +/- 20% (-1.0 to -2.3 mmol/d). Normally distributed  *Blakely T, Foster R, Wilson N, BODE³ Team (2012) Burden of Disease Epidemiology, Equity and Cost-Effectiveness (BODE3) Study Protocol. Version 2.1. Technical Report No.3. Wellington: Department of Public Health, University of Otago, Wellington, December 2012. http://www.otago.ac.nz/wellington/otago042986.pdf*  *OECD (2013) New international comparisons of GDP and consumption based on purchasing power parities for the year 2011. Paris*  **Mandatory 3G reformulation**: Effect size: 12.9 mmol/d reduction overall with SD at +/- 10% of this. Normally distributed.  *Nghiem N, Wilson N, Blakely T (2014) Validation Issues Relating to the Cardiovascular Disease Model Developed in the BODE³ Programme. Wellington: Department of Public Health, University of Otago.* [*http://www.otago.ac.nz/wellington/otago070189.pdf*](http://www.otago.ac.nz/wellington/otago070189.pdf)  **Mandatory all reformulation**: Effect size: 22.8 mmol/d reduction overall with SD at +/- 10% of this. Normally distributed  *Nghiem N, Wilson N, Blakely T (2014) Validation Issues Relating to the Cardiovascular Disease Model Developed in the BODE³ Programme. Wellington: Department of Public Health, University of Otago.* [*http://www.otago.ac.nz/wellington/otago070189.pdf*](http://www.otago.ac.nz/wellington/otago070189.pdf)  **UK package (multiple policies)**: Effect size: 3.2 mmol/d reduction per adult annually over the seven year period (22.7 mmol/d overall) with SD at +/- 10% of this. Normally distributed.  *Barendregt J, Oortmarssen GJ, Vos T, Murray CJL (2003) A generic model for the assessment of disease epidemiology: the computational basis of DisMod II. Popul Health Metr 1: 4. PMID: 12773212*  **Mass media campaign**: Effect size: 0.97 mmol/d reduction per adult annually over the seven year period (6.8 mmol/d overall) with SD at +/- 30% of this. Normally distributed.  *Barendregt J, Oortmarssen GJ, Vos T, Murray CJL (2003) A generic model for the assessment of disease epidemiology: the computational basis of DisMod II. Popul Health Metr 1: 4. PMID: 12773212*  **Tax**: Effect size: Variable annual reductions to keep under the maximal level of 20% change in any year. The highest reduction was in the first year at 6.5 mmol/d per adult  *Law MR, Frost CD, Wald NJ (1991) By how much does dietary salt reduction lower blood-pressure? 1. Analysis of observational data among populations. British Medical Journal 302: 811–815. PMID:2025703 34.*  *Lewington S, Clarke R, Qizilbash N, Peto R, Collins R (2002) Age-specific relevance of usual blood pressure to vascular mortality: a meta-analysis of individual data for one million adults in 61 prospective studies. Lancet 360: 1903–1913. PMID: 1249325535.*  *Law MR, Morris JK, Wald NJ (2009) Use of blood pressure lowering drugs in the prevention of cardio-vascular disease: meta-analysis of 147 randomised trials in the context of expectations from prospective epidemiological studies. British Medical Journal 338: b1665. doi: 10.1136/bmj.b1665 PMID:1945473736.*  *OECD OECD Health Statistics 2014: How does New Zealand compare? Paris: OECD, 2014. http://www.oecd.org/els/health-systems/Briefing-Note-NEW-ZEALAND-2014.pdf* |
| **Study ID** | **Study Design** | **Study Aim** | **Policy(ies) Analysed** | **Participants & sample size** | **Geographical Scope** | **Methods** | **Outcomes** | **Study Limitations** | **Sources referenced & Comments** |
| Collins et al. (2014)^68^ | Modelling study | To estimate the cost-effectiveness of four population health policies to reduce dietary salt intake on an English population to prevent coronary heart disease (CHD) | 1. 1. Health promotion campaign 2. 2. Labelling 3. 3. Voluntary salt reformulation 4. 4. Mandatory salt reformulation | English population | United Kingdom | The validated IMPACT CHD model was used to quantify and compare four policies. The effectiveness of these policies in reducing salt intake, and hence blood pressure, was determined by a systematic literature review. The model calculated the reduction in mortality associated with each policy, quantified as life-years gained over 10 years | *Primary outcomes (salt intake reductions estimated):*   - Campaign = 0.16g/d - Labelling = 0.16g/d - Voluntary reformulation = 1.21g/d - Mandatory reformulation = 1.62g/d   *Secondary outcomes:*   - Change4life and labeling might each gain approximately 1960 life-years - Voluntary reformulation might gain 14,560 life-years - Mandatory reformulation might gain 19,320 life-years | The model assumed a single step change in policy, moving in one year from the baseline of doing nothing to a fully implemented policy in the next year. In reality, there is likely to be a phased implementation with dietary salt levels reducing over time.  The CHD patient numbers were assumed to remain the same in subsequent years, which is likely to represent an overestimate. It also assumed that there would not be any detectable difference in taste of the foods or changes in consumers’ preferences | **Health Promotion** (2%)  *Food Standards Agency. Salt reduction strategy. Available from: http://www.food.gov.uk/scotland/scotnut/salt/strategy#.UW-9QUpLkRJ[Accessed April 18, 2013].*  *Unal B, Critchley J, Fidan D, Capewell S. Life-years gained from modern cardiological treatments and population risk factor changes in England and Wales, 1981-2000. Am J Pub Health 2005;95:103–8*  *Department of Health Vascular Programme Team. Treatment of Heart Attack National Guidance. Final Report of the National Infarct Angioplasty Project (NIAP). London, UK: Department of Health, 2008*  **Labelling** (2%)  *Food Standards Agency. Salt reduction strategy. Available from: http://www.food.gov.uk/scotland/scotnut/salt/strategy#.UW-9QUpLkRJ[Accessed April 18, 2013].*  **Voluntary reformulation** (15%) *National Institute for Health and Care Excellence. NICE Public Health Guidance 25: Prevention of Cardiovascular Disease at Population Level. London, UK: NICE, 2010*  *Craig R, Mindell J. Health Survey for England 2006. Leeds, UK: The Information Centre, 2008*  **Mandatory reformulation** (20%) *Craig R, Mindell J. Health Survey for England 2006. Leeds, UK: The Information Centre, 2008* |
| **Study ID** | **Study Design** | **Study Aim** | **Policy(ies) Analysed** | **Participants & sample size** | **Geographical Scope** | **Methods** | **Outcomes** | **Study Limitations** | **Sources referenced & Comments** |
| Gillespie et al. (2015)^69^ | Modelling study | To forecast the potential impact on English adults of policies implemented during the 2015 UK parliament, projecting the health consequences to 2025 | 1. 1. Mandatory reformulation 2. 2. Voluntary reformulation 3. 3. Social marketing 4. 4. Nutrition labelling |  | England | IMPACT_SEC_ was used, a validated CHD policy model, to link policy implementation to salt intake, systolic blood pressure and CHD mortality. The effects of mandatory and voluntary product reformulation, nutrition labelling and social marketing were forecasted. To inform our forecasts, they elicited experts’ predictions on further policy implementation up to 2020. They then modelled the effects on CHD mortality up to 2025 and simultaneously assessed the socio-economic differentials of effect | *Primary outcomes*  By 2020, they forecasted that mandatory reformulation (with a high impact, 30% reduction in salt content) might reduce dietary salt intake by around 1.45 g/day. Furthermore, the effect in the most deprived quintile would exceed that in the most affluent quintile by around 14% or 0.19 g/day, thus reducing inequality. Their forecast also indicated that mandatory reformulation would be more inequality-reducing than further voluntary reformulation.  The agentic options, social marketing (-0.027 to -0.13g/day) and nutrition labelling (-0.031 to -0.16g/day), might be a third as effective as voluntary reformulation (-0.48g/day), and a tenth as effective as our high impact mandatory scenario.  *Secondary outcomes*  Mandatory reformulation might prevent or postpone 4,500 (2,900–6,100) CHD deaths in total, with the effect greater by 500 (300–700) deaths or 85% in the most deprived than in the most affluent. Further voluntary reformulation was predicted to be less effective and inequality-reducing, preventing or postponing 1,500 (200–5,000) CHD deaths in total, with the effect greater by 100 (−100–600) deaths or 49% in the most deprived than in the most affluent. Further social marketing and improvements to labelling might each prevent or postpone 400–500 CHD deaths, but minimally affect inequality | For the agentic policy options, they lacked data on individual responsiveness to the information they receive. Important to acknowledge that, in practice, each policy option will fit within a multi-component policy strategy, where policies undoubtedly interact | **Social marketing and nutrition labelling; reduction of 2.39g/day modelled for each from:**  *Rees K, Dyakova M, Wilson N, Ward K, Thorogood M, Brunner E. Dietary advice for reducing cardiovascular risk. The Cochrane database of systematic reviews. 2013; 12: CD002128. doi: 10.1002/ 14651858.CD002128.pub5 PMID: 24318424*  **Product reformulation**  *2010 Health Survey for England*  *2011 Living Costs and Food Survey* |
| **Study ID** | **Study Design** | **Study Aim** | **Policy(ies) Analysed** | **Participants & sample size** | **Geographical Scope** | **Methods** | **Outcomes** | **Study Limitations** | **Sources referenced & Comments** |
| Wilcox et al. (2014)^70^ | Modelling study | To present a cost-effectiveness analysis of salt reduction policies to lower CHD in Syria | 1. 1. Health promotion campaign 2. 2. Labelling 3. 3. Reformulation |  | Syria | Costs and benefits of a health promotion campaign about salt reduction (HP); labelling of salt content on packaged foods (L); reformulation of salt content within packaged foods (R); and combinations of the three were estimated over a 10-year time frame using the IMPACT CHD model for Syria | Health promotion campaign:   - 5% reduction in salt intake - 252 deaths prevented - 5,679 LYG   Labelling:   - 10% reduction in salt intake - 497 deaths prevented - 11,192 LYG   Reformulation:   - 10% reduction in salt intake - 497 deaths prevented - 11,192 LYG   Reformulation + HP  Reformulation + Labelling   - 15% reduction in salt intake - 735 deaths prevented - 16,543 LYG   All 3 policies:   - 30% reduction in salt intake - 1,413 deaths prevented - 31,674 LYG | Data from a comparable neighbouring population (Palestine) were used, when possible, if Syrian data were unavailable. The source of dietary salt intake and evidence of the effectiveness of the policies to reduce intake were extracted from other countries. The IMPACT CHD model assumed that the 2010 level of CHD rates persisted through the 10-year time frame. Rates, however, are increasing in Syria due to rising trends in population risk factors | **Duplicate analysis of Mason**  *Asaria P, Chisholm D, Mathers C et al. 2007. Chronic disease prevention: health effects and financial costs of strategies to reduce salt intake and control tobacco use. The Lancet 370: 2044–53*  *Cappuccio F, Capewell S, Lincoln P, McPherson K (2011) Policy options to reduce population salt intake. BMJ 343:d4995*  *Hooper L, Bartlett C, Davey Smith G, Ebrahim S (2004) Advice to reduce dietary salt for prevention of cardiovascular disease. Cochrane Database Syst Rev. Issue 1. doi:10.1002/14651858. CD003656.pub2. (Art No: CD003656)*  *Mohan S, Campbell NR, Willis K (2009) Effective population-wide public health interventions to promote sodium reduction. Can Med Assoc J 181(9):605–609*  *Smith-Spangler C, Juusola J, Enns E, Owens D, Garber A (2010) Population strategies to decrease sodium intake and the burden of cardiovascular disease. Ann Intern Med 152:481–487* |
| **Study ID** | **Study Design** | **Study Aim** | **Policy(ies) Analysed** | **Participants & sample size** | **Geographical Scope** | **Methods** | **Outcomes** | **Study Limitations** | **Sources referenced & Comments** |
| Mason et al. (2014)^71^ | Modelling study | To present an economic evaluation of population based salt reduction policies in Tunisia, Syria, Palestine and Turkey | 1. 1. Health promotion campaign (HP) 2. 2. Labelling (L) 3. 3. Mandatory reformulation (R) |  | Tunisia, Syria, Palestine and Turkey | Estimates of the effectiveness of salt reduction on blood pressure were based on a literature review. The reduction in mortality was estimated using the IMPACT CHD model specific to that country. Cumulative population health effects were quantified as life years gained (LYG) over a 10 year time frame | *Tunisia:*   - HP = 1,151 LYG ; 39 - L = 2,272 LYG - R = 2,272 LYG - All 3 policies = 6,455 LYG   *Syria:*   - HP = 5,679 LYG - L = 11,192 LYG - R = 11,192 LYG - All 3 policies = 31,674 LYG   *Palestine:*   - HP = 479 LYG - L = 945 LYG - R = 945 LYG - All 3 policies = 2,682 LYG   *Turkey:*   - HP = 68,816 LYG - L = 135,221 LYG - R = 135,221 LYG - All 3 policies = 199,303 LYG   The combination of all three policies (reducing salt consumption by 30%) generated estimated cost savings of $235,000,000 and 6,455 LYG in Tunisia; $39,000,000 and 31,674 LYG in Syria; $6,000,000 and 2682 LYG in Palestine and $1,3000,000,000 and 378,439 LYG in Turkey | The analysis in this study was confined to a ten year time horizon. It thus did not take into account any health care costs postponed to the distant future. The methodology for the collection of cost data varied across countries due to differences in data availability | **HP** (effectiveness 5%)  *Hooper L, Bartlett C, Davey Smith G (2004) Advice to reduce dietary salt for prevention of cardiovascular disease. Cochrane Database of Syst Revs. Issue 1.DOI:10.1002/14651858.CD003656.pub2*  **Labelling** (10% effectiveness)  *Cappuccio F, Capewell S, Lincoln P, McPherson K (2011) Policy options to reduce population salt intake. BMJ 343:d4995*  **Mandatory reformulation** (effectiveness 10%)  *Smith-Spangler C, Juusola J, Enns E, Owens D, Garber A (2010). Population Strategies to Decrease Sodium Intake and the Burden of Cardiovascular Disease. Ann Intern Med 152: 481–7*  **MR+HP** and **HP+L** (effectiveness 15%)  *Asaria P, Chisolm D, Mathers C, Ezzati M, Beaglehole R (2007) Chronic disease prevention: health effects and financial costs of strategies to reduce salt intake and control tobacco use. Lancet 370: 2044–53* |
| **Study ID** | **Study Design** | **Study Aim** | **Policy(ies) Analysed** | **Participants & sample size** | **Geographical Scope** | **Methods** | **Outcomes** | **Study Limitations** | **Sources referenced & Comments** |
| Pietinen et al. (2008)^74^ | Modelling study | To estimate the impact of choosing food products labelled either as low or high in salt on salt intake in the Finnish adult population | 1. Salt labelling | 2007 subjects aged 25-64 years | Finland | The National FINDIET 2002 survey was used. Sodium intake was calculated based on the Fineli food composition database including the sodium content of natural and processed foods as well as the salt content of recipes. The distribution of salt intake was calculated in different ways: the present situation; assuming that all breads, cheeses, processed meat and fish, breakfast cereals and fat spreads consumed would be either 'lightly salted' or 'heavily salted' based on the current labelling practice; and, in addition, assuming that all foods would be prepared with 50% less or more salt | The calculated salt intake was 9.9 g in men and 6.8 g in women, when all persons were included. If the entire population were to choose low-salt breads, cheeses, processed meat and fish, fat spreads, and breakfast cereals, then salt intake could be lowered by 1.5 g in men and by 0.9 g in women. If everybody was to select high-salt products, then salt intake would go up by 1.9 g in men and by 1.2 g in women. Thus, the potential difference between the low and the high alternatives would be 3.4 g in men and 2.9 g in women. If all prepared foods had a reduced salt content, the mean salt intake would go further down by 2.3 g in men and by 1.7 g in women. When under reporters were excluded, the mean salt intake was 11.1 g in men and would go down to 9.5 g if all men chose lightly salted products and further down to 6.8 g if also all prepared foods would have a lower salt content. In women, the respective numbers are 7.8, 6.7 and 4.9 g. If heavily salted products were chosen systematically, salt intake would be 13.1 g in men and 9.2 g in women. In the worst scenarios, the levels would be 15.4 and 10.8 g, respectively | Based on self-reported data and predictions | In this analysis, excluding under-reporters gives a more realistic picture of the present situation and the mean calculated intakes, 11.1 g in men and 7.8 g in women, in line with the 24-hour urinary sodium  *Mannisto S, Ovaskainen M-L, Vasta L, eds. The National Findiet 2002 Study. Publications of the National Public Health Institute B3/2003. Helsinki: Hakapain: OY, 2003.* |
| **Study ID** | **Study Design** | **Study Aim** | **Policy(ies) Analysed** | **Participants & sample size** | **Geographical Scope** | **Methods** | **Outcomes** | **Study Limitations** | **Sources referenced & Comments** |
| Temme et al. (2010)^75^ | Modelling study | To evaluate the effects of changed food compositions according to health logo criteria on the intake of saturated fat, sugar and sodium in a Dutch population of young adults | 1. Labelling (health logos) | 750 young Dutch adults aged 19-30 years old | Netherlands | Foods in the Dutch food composition table were evaluated against nutrient criteria for logo eligibility. Three replacement scenarios were compared with the nutrient intake 'as measured' in the Dutch consumption survey. The foods not complying with health logo criteria were replaced either by 'virtual' foods exactly complying with the health logo criteria, with real 2007 market shares (scenario I) and 100% market shares (scenario II), or by existing similar foods with a composition that already complied with the health logo criteria (scenario III) | The estimated usual intake of sodium in the study population, at baseline, was 2.9 (95 % CI 2.8, 2.9) g/day. For sodium, in a 100 % market share scenario (scenario II), sodium reduction expected is 0.1g/d (4% reduction). The intake in this scenario is not significantly different from the intake in the baseline situation. With the current market share sodium intake is similar to the reference intake. In scenario III, when all non-complying foods are replaced with foods complying with health logo criteria, sodium intake reduced by 23% to 2.2g/d. The main contributors to this reduction are meat (products; 8.2% lower intake from meat products compared with the reference situation), bread, cheese, nutty and savoury snacks and herbs (2.3% lower intake compared with the reference situation) | Type of data the calculations are based on. Health logo labelling is voluntary and is initiated by a few large companies. The market share for 2007 is a snapshot; the market for foods and the availability of foods with health logos change rapidly. Self-reported data used. In the intake assessments, it was assumed that a person would replace a non-complying food for a similar complying food, and would not eat more of it | *Ocke M, Hulshof K & van Rossum C (2005) The Dutch National Food Consumption Survey 2003. Methodological issues. Arch Public Health 63, 227–241.* |
| **Study ID** | **Study Design** | **Study Aim** | **Policy(ies) Analysed** | **Participants & sample size** | **Geographical Scope** | **Methods** | **Outcomes** | **Study Limitations** | **Sources referenced & Comments** |
| De Menezes et al. (2013)^76^ | Modelling study | To evaluate the impact of introducing products that are in agreement with the Choices criteria in the usual diet of the Brazilian population | 1. Food labelling |  | Brazil | Data on industrialized and packed products available in the market, in São Paulo State, in the period of January to July 2011, were collected. The sources of nutritional information were product labels or websites. The nutritional information on SAFA (g/100 g), TFA (g/100 g), sodium (mg/100 g), added sugar (g/100 g), DF (g/100 g) and energy (kcal/100 g and kJ/100 g) were collected. A food composition database of industrialized products was created (1720 products) and their data were evaluated according to the Choices criteria | It is possible to observe that the Choices products would promote a reduction in sodium from 1% (China) to 47% (Brazil). Sodium would still be considered an important reduction, 36% in relation to the TM, but it would be 3022 mg/day, which is above the recommended by the program (2600 mg/day).  **Sodium (mg/day)**  *Typical menus (TM)*   - 4743 ± 498   *Choices menus (CM)*   - 2527 ± 51   *Choices menus energy (CME) – same as CM, but adjusted for energy of TM*   - 3040 ± 346 |  |  |
| **Study ID** | **Study Design** | **Study Aim** | **Policy(ies) Analysed** | **Participants & sample size** | **Geographical Scope** | **Methods** | **Outcomes** | **Study Limitations** | **Sources referenced & Comments** |
| Roodenburg et al. (2013)^77^ | Modelling study | To describe a nutrient intake modelling method to evaluate nutritional criteria by investigating the potential effect on nutrient intakes | 1. Food labelling |  | Netherlands | Data were combined from the 2003 Dutch food consumption survey in young adults (aged 19–30) and the Dutch food composition table into the Monte Carlo Risk Assessment model. Three scenarios were calculated: the ‘‘actual intakes’’ (scenario 1) were compared to scenario 2, where all foods that did not comply were replaced by similar foods that did comply with the Choices criteria. Scenario 3 was the same as scenario 2 adjusted for the difference in energy density between the original and replacement food. Additional scenarios were calculated where snacks were not or partially replaced and stratified analyses for gender, age, Body Mass Index (BMI) and education | A reduction of -23% for sodium was seen for sodium compared to the ‘actual scenario’.  **Sodium (mg)**   - *Actual*: 2949 - *Choices*: 2261 - *Choices energy adjusted*: 2590 - *Snacks (partially replaced)*: 2300 - *Snacks (not replaced)*: 2349 | Replacements chosen may still be susceptible to some subjectivity and bias. Product acceptability not taken into consideration. The same replacement food has been used for a large number of snacks. Snacks are usually eaten for indulgence; therefore it is unrealistic to assume that consumers will replace all snacks with one and the same healthier alternative | *Ocke MC HK, Buurma-Rethans EJM, Rossum CTM van, Drijvers JJMM, Brants HAM, et al. (2004) Food consumption survey, 2003: summary of procedures and evaluation. Available: http://www.rivm.nl/en/Documents_ and_publications/Scientific/Reports/2004/oktober/National_food_ consumption_survey_2003_Summary_of_procedures_and_evaluation. Accessed 2013 Jul 24.*  *(2006) NEVO-tabel Nederlands Voedingsstoffenbestand (Dutch Food Composition Table). The Hague: Stichting Nederlands Voedingsstoffen bestand* |
| **Study ID** | **Study Design** | **Study Aim** | **Policy(ies) Analysed** | **Participants & sample size** | **Geographical Scope** | **Methods** | **Outcomes** | **Study Limitations** | **Sources referenced & Comments** |
| Choi et al. (2015)^41^ | Modelling study | To estimate the cardio-vascular impact of the expanded NSRI among different segments of the US population and under varying possible producer and consumer responses to the initiative | Reformulation | 10,000 adults aged 18 to 85 years | US | They developed and validated a stochastic micro simulation model of hypertension, MI, and stroke morbidity and mortality, using data from food producers on sodium reduction among foods, linked to 24-hour dietary recalls, blood pressure, and cardiovascular histories from the National Health and Nutrition Examination Survey | Expansion of the initiative to ensure all restaurants and manufacturers reach agreed-upon sodium targets would be expected to avert from 0.9 to 3.0 MIs (a 1.6%– 5.4% reduction) and 0.5 to 2.8 strokes (a 1.1%–6.2% reduction) per 10,000 Americans per year over the next decade, after incorporating consumption patterns and variations in the effect of sodium reduction on blood pressure among different demographic groups. The expanded NSRI covering both packaged and restaurant food items would be expected to reduce mean daily sodium intake by 447 mg per person per day on average, or 13.0% (95% CI, 12.9–13.1) which would correspond to a reduction in the prevalence of hypertension by 3.0% (95% CI, 2.7–3.3) and MI and stroke incidence by 5.4% (95% CI, 4.9–5.9) and 6.3% (95% CI, 5.7– 6.9), respectively (Table 2). The initiative would be expected to lower MI and stroke mortality by 5.1% (95% CI, 3.8–6.3) and 4.9% (95% CI, 3.2–6.7), respectively. If the NSRI included only restaurant food items, the program would lower MI and stroke mortality by an estimated 2.7% (95% CI, 1.4–4.0) and 2.1% (95% CI, 0.4–3.9), respectively. Hence, most of the benefit from the program would likely be due to sodium changes among packaged foods. The projected benefits in MI and stroke incidence would be largest among non-Hispanic white men in the 40- to 59- year-old age cohort (experiencing a 5.5% [95% CI, 5.1–5.9] decline in MI incidence and a 7.8% [95% CI, 7.2–8.3] decline in stroke incidence), after accounting for their typical food choice preferences and NSRI-associated sodium reductions.  Mexican men aged 60 to 85 years had a slightly but not significantly lower percentage change in incident MI than non-Hispanic white men aged 40 to 59 years. Mortality rates also decreased the most in this cohort, by 5.6% (95% CI, 5.0–6.3) for MI and by 8.0% (95% CI, 6.3–9.7) for stroke. In the simulations including both restaurant and packaged foods in the NSRI, we estimated that large numbers of people, 1217 (95% CI, 1205–1229) per 10,000 persons, would move from above the threshold of 3 g/d of sodium recently correlated to increased mortality18 to below that threshold due to the NSRI. Approximately 901 (95% CI, 893–909) per 10,000 persons would be expected to lower sodium consumption below the threshold of 2 g/d due to the NSRI. The group at particular risk for moving below this threshold was older women | They modelled the effects of sodium reduction on blood pressure based on published data, assuming that the health benefits of sodium reduction were mediated through changes in blood pressure observed in such trials. Data from NHANES was used, which are subject to the limitations of survey studies, including recall biases, acceptability biases, and underreporting. They did not account for potential effects of sodium reduction that are not directly associated with blood pressure reduction or hypertension-related pathologies such as chronic kidney disease, which may garner additional benefits from the program | The NSRI has announced sodium reduction agree-  ments for each type of packaged and restaurant  food. The expansion is being modelled  *New York City Department of Health and Mental Hygiene.*  *National Salt Reduction Initiative packaged food categories and*  *targets. 2010. Available from: http://www.nyc.gov/html/doh/*  *downloads/pdf/cardio/packaged-food-targets.pdf*  *New York City Department of Health and Mental Hygiene.*  *National Salt Reduction Initiative restaurant food categories and*  *targets. 2010. Available from: http://www.nyc.gov/html/doh/*  *downloads/pdf/cardio/cardio-salt-nsri-restaurant.pdf* |
| **Study ID** | **Study Design** | **Study Aim** | **Policy(ies) Analysed** | **Participants & sample size** | **Geographical Scope** | **Methods** | **Outcomes** | **Study Limitations** | **Sources referenced & Comments** |
| Murray et al. (2003)^79^ | Modelling study | To report estimates of the population health effects, and costs of selected interventions to reduce the risks associated with high cholesterol concentrations and blood pressure in areas of the world with differing epidemiological profiles | 1. 1. Voluntary reformulation 2. 2. Mandatory reformulation |  | South East Asia, Latin America, Europe | Effect sizes were derived from systematic reviews or meta-analyses, and the effect on health outcomes projected over time for populations with differing age, sex, and epidemiological profiles. Incidence data from estimates of burden of disease were used in a four-state longitudinal population model to calculate DALYs averted and patients treated. Costs were taken from previous publications, or estimated by local experts, in 14 regions | Non-personal health interventions, including government action to stimulate a reduction in the salt content of processed foods, were cost-effective ways to limit CVD and could avert over 21 million DALYs per year worldwide. They demonstrated that, over 10 years (from 2006 to 2015), a 15% reduction in mean population salt intake could avert 8.5 million cardiovascular deaths. Measures to decrease salt intake appear potentially very cost effective, with legislation being more cost effective than voluntary agreements under the assumption that it would lead to the larger reduction in dietary salt intake.  *Secondary outcomes*  *Europe estimates*  Voluntary reformulation:   - 7 X10^6^ DALYs averted - $44 per DALY   Mandatory measures:   - 13x10^6^ DALYs averted - $23 per DALY   *South East Asia estimates*  Voluntary reformulation:   - 5X10^6^ DALYs averted - $37 per DALY   Mandatory measures:   - 10 x10^6^ DALYs averted - $19 per DALY   *Latin America estimates*  Voluntary reformulation:   - 3 X10^6^ DALYs averted - $24 per DALY   Mandatory measures:   - 6 x10^6^ DALYs averted - $13 per DALY |  | Unclear where effect measures were obtained |
| **Study ID** | **Study Design** | **Study Aim** | **Policy(ies) Analysed** | **Participants & sample size** | **Geographical Scope** | **Methods** | **Outcomes** | **Study Limitations** | **Sources referenced & Comments** |
| Rubinstein et al. (2010)^80^ | Modelling study | To estimate the burden of acute CHD and stroke and the cost-effectiveness of preventative population-based and clinical interventions | 1. Salt reduction in bread | Adult population over 35 years old | Argentina | An epidemiological model was built incorporating prevalence and distribution of high blood pressure, high cholesterol, hyperglycemia, overweight and obesity, smoking, and physical inactivity, obtained from the Argentine Survey of Risk Factors dataset. Population Attributable Fraction (PAF) of each risk factor was estimated using relative risks from international sources. Total fatal and non-fatal events, PYLL and DALYs were estimated. Costs of event were calculated from local utilization databases and expressed in international dollars (I$). Incremental cost-effectiveness ratios (ICER) was estimated for reducing salt in bread | Argentina has an average individual consumption of 12 grams of salt per day, 3.4 grams coming from bread.  *Reducing salt in bread is cost-saving:*   - Total costs: 193,576.23 - Net total cost: -946,580.87 - DALYs averted: 672.80 - % of DALYs saved: 0.11% - ICER per DALY saved: -1,406.93 | The risk factors included in the model were limited to those that were specifically addressed in the national survey as they were specifically defined. Since the prevalence of risk factors was obtained from self-reports of participants and not from direct measures, they were defined dichotomously or categorically for the calculation of the PAR. The study synthesized data from many sources and used several assumptions in the design of the model. Some inputs were derived from international sources | **Salt reformulation in bread**  *He FJ, MacGregor GA: Effect of longer-term modest salt reduction on blood pressure. Cochrane Database Syst Rev 2004, CD004937*  *Hooper L, Bartlett C, Davey SG, Ebrahim S: Advice to reduce dietary salt for prevention of cardiovascular disease. Cochrane Database Syst Rev*  *2004, CD003656.* |
| **Study ID** | **Study Design** | **Study Aim** | **Policy(ies) Analysed** | **Participants & sample size** | **Geographical Scope** | **Methods** | **Outcomes** | **Study Limitations** | **Sources referenced & Comments** |
| Smith-Spangler et al. (2010)^81^ | Modelling study | To assess the cost-effectiveness of 2 population strategies to reduce sodium intake: government collaboration with food manufacturers to voluntarily cut sodium in processed foods, modelled on the United Kingdom experience, and a sodium tax | 1. 1. Voluntary reformulation 2. Sodium tax | Adults aged 40 to 85 years | US | A Markov model was constructed with 4 health states: well, acute myocardial infarction (MI), acute stroke, and history of MI or stroke using data from the Medical Panel Expenditure Survey (2006), Framingham Heart Study (1980 to 2003), Dietary Approaches to Stop Hypertension trial, and other published data | *Secondary outcomes:*  Collaboration with industry to achieve a 9.5% reduction in population sodium intake would result in a 1.25–mm Hg decrease in mean SBP of persons aged 40 to 85 years. This blood pressure reduction, in turn, would avert 513 885 strokes and 480 358 MIs and increase life-years lived by more than 1.3 million over the lifetime of U.S. adults aged 40 to 85 years alive today, saving $32.1 billion in direct medical costs. A sodium tax achieving a 6% decrease in sodium intake and a 0.93–mm Hg decrease in mean SBP would avert 327 892 strokes and 306 137 MIs and increase life-years lived by 840 113, saving $22.4 billion over the lifetime of adults aged 40 to 85 years alive today | Efforts to reduce population sodium intake could result in other dietary changes that are difficult to predict | **Collaboration with the industry – 9.5%**  *National Centre for Social Research/ Human Nutrition Research. An assessment of dietary sodium levels among adults (aged 19-64) in the UK general population in 2008, based on analysis of dietary sodium in 24 hour urine samples. June 2008. Accessed at* [*www.food.gov.uk/multimedia/pdfs/08sodiumreport*](http://www.food.gov.uk/multimedia/pdfs/08sodiumreport)*.pdf on 16 February 2010.*  *Food Standards Agency. Salt levels continue to fall. July 2008. Accessed at www.food.gov.uk/news/newsarchive/2008/jul/sodiumrep08 on 16 February 2010.*  *Henderson L, Irving K, Gregory J, et al. The National Diet & Nutrition Survey: Adults Aged 19–64 Years: Vitamin and Mineral Intake and Urinary Analytes (2000–2001), Vol 3. London: Her Majesty’s Stationery Office; 2003. Accessed at www.food.gov.uk/multimedia/pdfs/ndnsv3.pdf on 20 February 2010.*  **They also modelled a 6% reduction in salt intake for a 40% sodium tax.**  *Orzechowki W, Walker R. The Tax Burden on Tobacco. Arlington, VA:*  *Orzechowski & Walker; 2006* |
| **Study ID** | **Study Design** | **Study Aim** | **Policy(ies) Analysed** | **Participants & sample size** | **Geographical Scope** | **Methods** | **Outcomes** | **Study Limitations** | **Sources referenced & Comments** |
| Konfino et al. (2013)^82^ | Modelling study | To project the impact of Argentina’s sodium reduction policies under two scenarios - the 2-year intervention currently being undertaken and a more persistent 10 year sodium reduction strategy | Reformulation |  | Argentina | They used Argentina-specific data on sodium excretion by sex and projected the impact of the current strategy on sodium consumption and blood pressure decrease. The projected impact of sodium reduction policies on CVD using the Cardiovascular Disease (CVD) Policy Model was assessed, adapted to Argentina, modeling two alternative policy scenarios over the next decade | Using the spot urines from the Argentina La Pampa Pilot study, after estimating 24-hour urinary sodium excretion, they estimated a mean sodium consumption of 4832 mg/day for men, 3983 mg/day for women and 4407 mg/day on average, what is equivalent to 12.1, 10 and 11 grams/day of salt respectively. The current initiative is projected to reduce the mean sodium consumption by 387 mg/day in men and 319 mg/day in women (Scenario 1). If this strategy was maintained for 10 years (Scenario 2) it is projected to reduce sodium intake by 1933 mg/day in men and 1593 mg/day in women. Scenario 1 would reduce systolic blood pressure by 0.93 mmHg up to 1.81 mmHg depending on the population subgroup and Scenario 2 would reduce systolic blood pressure by 4.66 mmHg up to 9.04 mmHg depending on the subgroup. They estimated that the impact the current salt initiative implemented in Argentina (Scenario 1) would avert about 19,000 deaths of which 6,000 are CHD deaths and 2,000 are stroke deaths. They also projected that the ‘‘Less salt More Life’’ initiative can avert about 13,000 total myocardial infarctions and 10,000 total stroke cases from 2013–2023. These results represent a reduction of 0.6% in total mortality in 35 years and older adults, 1.5% in total myocardial infarctions and 1% in total stroke cases in the next decade. If this sodium reduction strategy were continued for 10 years, setting progressive goals simulated in Scenario 2, the impact would be even greater: 55,000 deaths, with 16,000 CHD deaths and 5,000 stroke deaths, 38,000 total myocardial infarctions and 27,000 total strokes would be avoided in the next 10 years. These reductions would translate into a decrease of 2% in overall mortality in 35 years and older adults, as well as a 4.3% in total myocardial infarctions and a 2.7% decrease in total strokes | The same sodium consumption in adults over 35 years was assumed accounting small differences reported in the literature for Argentina. They assumed that the sodium urine excretion representative of the Province of La Pampa reflected the sodium urine excretion of the entire Argentina. Need to consider that some variations in population’s ‘sodium consumption and possible replacements by other product in response to the lower sodium content may occur over time that could influence final estimated impact of the intervention | Since the ‘‘Less salt More Life’’ initiative aims to reduce 5–15% of sodium content in processed food, they used the mean value of that reduction (10%), applied to the 80% of sodium consumed that comes from processed foods  *Mattes R, Donnelly D (1991) Relative contributions of dietary sodium sources. J Am Coll Nutr 10: 383–393.*  They assumed that each gram of reduction in salt consumption would decrease 1.87 mmHg in systolic blood pressure of hypertensive and older than 65 years old and 1.17 mmHg in systolic blood pressure of non-hypertensive and/or less than 65 years.  *He FJ, MacGregor GA (2004) Effect of longer- term modest salt reduction on blood pressure. Cochrane Database Syst Rev 2001(2):CD000165, 3.* |
| **Study ID** | **Study Design** | **Study Aim** | **Policy(ies) Analysed** | **Participants & sample size** | **Geographical Scope** | **Methods** | **Outcomes** | **Study Limitations** | **Sources referenced & Comments** |
| Rubinstein et al. (2009)^83^ | Modelling study | To use generalised cost-effectiveness analysis to identify the most efficient interventions to decrease CVD | Reformulation in bread |  | Argentina | Estimates of effectiveness were entered into age and sex specific models to predict their impact in terms of age-weighted and discounted DALYs saved. To translate the age- and sex-adjusted incidence of CVD events into health changes, we used risk model software developed by WHO (PopMod). Costs of services were measured in Argentine pesos, and discounted at an annual rate of 3%. Different budgetary impact scenarios were explored | Concerning specific interventions, the strategy of lowering salt intake in the population through reducing salt in bread was found to be the most cost-effective (ARS $151 per DALY averted) followed by mass media campaign (ARS $547 per DALY averted).  *Less salt in bread*   - Total Cost per year (ARS$): - $ 87,471 - DALY Age weighted, 3% discounted per year: 579 - DALY No age weight 3% discounted per year: 713 - DALY # age weight, undiscounted per year: 1,107 - ARS$ (1)/DALY (2): $ 151 |  | **Reduction of salt in bread through voluntary agreement RR = 0.99**  *He FJ, MacGregor GA: How far should salt intake be reduced? Hypertension 2003, 42:1093-1099* |
| **Study ID** | **Study Design** | **Study Aim** | **Policy(ies) Analysed** | **Participants & sample size** | **Geographical Scope** | **Methods** | **Outcomes** | **Study Limitations** | **Sources referenced & Comments** |
| Hendriksen et al. (2014)^84^ | Modelling study | To evaluate the health benefits of salt-reduction strategies related to processed foods for the Dutch population | 1. Reformulation, substitution and reaching recommended intake | Dutch population aged 20 years and over | Netherlands | Three salt-reduction scenarios were developed: 1) substitution of high-salt foods with low-salt foods, 2) a reduction in the sodium content of processed foods, and 3) adherence to the recommended maximum salt intake of 6 g/d. Health outcomes were obtained in 2 steps: after salt intake was modelled into blood pressure levels, the Chronic Disease Model was used to translate modelled blood pressures into incidences of cardiovascular diseases, DALYs and life expectancies. Health outcomes of the scenarios were compared with health outcomes obtained with current salt intake | When the salt intake in the Netherlands remained the same throughout the coming 20 y (current salt intake scenario), 667,600 cases of AMI, 902,900 cases of CHF, and 896,500 cases of CVA were projected to occur. If salt intake is reduced to the  recommended maximum salt intake (6 g/d), 4.8% of cases of AMI, 1.7% of cases of CHF, and 5.8% of cases of CVA might be prevented.  Current salt intake: 8.4g/d. In the salt reduction processed foods scenario, the median salt intake would decrease by 28% (-2.3g/d). All-cause mortality might be reduced by 0.7% when salt intake was reduced to the recommended maximum salt intake. The same percentage would be found for the salt reduction in processed foods scenario, and a slightly higher reduction would be found for the substitution processed foods scenario. A 40-y-old individual would increase the number of DALYs lived by 0.5% (56,400 DALYs) if he would equal his salt intake to the recommended maximum intake for the rest of his life. Life expectancy for a 40-y-old individual would increase by 0.4% (0.15 y) in the recommended maximum intake scenario. Similar health gains were found for the salt reduction in processed foods scenario (DALYs: 56.000; 0.5%. LYG: 0.15y; 0.4%) and for the substitution of processed foods scenario (DALYs: 67.900; 0.6%. LYG: 0.18y; 0.4%) | Study was limited by any uncertainty related to the data entered into the model. They did not have national representative blood pressure levels available for individuals older than 69 y and had to estimate the salt intake in older adults. They used blood pressure categories instead of continuous blood pressure levels. Future trends in determinants or treatment of blood pressure or CVD may affect the outcomes of the model, but did not take these (largely unknown) changes into account | *For the 2 salt-reduction strategies, data from the DNFCS 2007–2010 and the specially prepared strategy-specific food-composition tables were combined to calculate usual salt intakes (including estimated use of discretionary salt, as described before)* |
| **Study ID** | **Study Design** | **Study Aim** | **Policy(ies) Analysed** | **Participants & sample size** | **Geographical Scope** | **Methods** | **Outcomes** | **Study Limitations** | **Sources references & Comments** |
| Ni Mhurchu et al. (2015)^87^ | Modelling study | To estimate the effects of health-related food taxes and subsidies on deaths prevented or postponed (DPP) in New Zealand | 1. Taxes |  | New Zealand | A macrosimulation model based on household expenditure data, demand elasticities and population impact fractions for 18 diet-related diseases was used to estimate effects of five tax and subsidy regimens. Price elasticity values for 24 major commonly consumed food groups in New Zealand were used, together with food expenditure data from national Household Economic Surveys. Changes in mortality from cardiovascular disease, cancer, diabetes and other diet-related diseases were estimated | A sodium tax might reduce sodium intakes by approximately 11% but would result in a 2% increase in saturated fat purchases due to positive cross-PEs between pork and products high in sodium. A 20% tax on major dietary sources of sodium might result in 2,000 (1300 to 2,700) DPP (6.8%) | The PEs they derived for their models were the best available national estimates but were obtained by linking the economic survey dataset to another dataset, the Food Price Index. These had a r elative small sample size and short time period. PEs only by population and not sub-groups. Theoretical assumptions were made about which cross-price elasticities to include. The model assumed that price elasticities were conditional. Uncertainty regarding potential supply-side responses to taxes or subsidies. Quantification of health gain in DPP instead of QALYs | **Sodium tax**  *University of Otago and Ministry of Health (2011) A focus on nutrition: key findings of the 2008/09 New Zealand adult nutrition survey. Wellington: Ministry of Health.* |
| **Study ID** | **Study Design** | **Study Aim** | **Policy(ies) Analysed** | **Participants & sample size** | **Geographical Scope** | **Methods** | **Outcomes** | **Study Limitations** | **Sources referenced & Comments** |
| Asaria et al. (2007)^104^ | Modeling study | To investigate how many deaths could potentially be averted over 10 years by implementation of selected population-based interventions, and calculated the financial costs of their implementation | 1. 1. Voluntary reformulation 2. Mass media campaign |  | 23 low and middle income countries | For each of the 23 countries, and for all years between 2006 and 2015, they used methods from the WHO Comparative Risk Assessment project to estimate the effects of successful implementation of the two strategies. They calculated the proportion of chronic disease deaths from specific causes that could be averted if the distributions of mean systolic blood pressure and tobacco exposure were shifted to lower levels (the “potential impact fraction”) for different age-groups and sexes in adults | 8.5 million deaths would be averted by implementation of the salt-reduction strategy alone.  *15% reduction in mean intake*   - salt reduction of 1.69g/d - 8.4 million CVD deaths averted   *30% reduction in mean intake*   - salt reduction of 3.38g/d - 16.0 million CVD deaths averted   *Reduction to 5g/d*   - salt reduction of 6.28g/d - 28.3 million CVD deaths averted | Underlying uncertainties in the data sources we used. Because data about salt consumption in low-income and middle-income countries were not available for many of the countries modelled, they had to be inferred from regional estimates. Furthermore, they did not have time trends for these data | **The results include both voluntary reformulation and mass media campaigns**  Not very clear where the 15% was obtained from |
| **Study ID** | **Study Design** | **Study Aim** | **Policy(ies) Analysed** | **Participants & sample size** | **Geographical Scope** | **Methods** | **Outcomes** | **Study Limitations** | **Sources referenced & Comments** |
| Dodhia et al. (2012)^105^ | Modelling study | To assess the impact of known cost-effective interventions in terms of the avoidable CVD burden and costs by comparing these strategies to the current situation | 1. 1. Health promotion 2. 2. Reformulation | Population aged over 16 years | England | The avoidable CVD outcomes simulating the English population aged over 16 years were modelled with Excel spreadsheets for the current prevention/treatment and following various interventions over a 10-year time frame. Costs are reported from the health service perspective. They analysed relative cost-effectiveness, undertook sensitivity analysis and measured relative impacts of different strategies on avoidable burden of disease | Population lifestyle interventions.  *NA – 2mmHg*   - IHD events avoided: 56.116 - Stroke events avoided: 98.497 - IHD deaths avoided: 26.781 - Stroke deaths avoided: 39.557 - DALYs averted: 238.043 - Cost per DALY: -2.708   *NA – 5mmHg*   - IHD events avoided: 120.138 - Stroke events avoided: 257.508 - IHD deaths avoided: 57.322 - Stroke deaths avoided: 103.492 - DALYs averted: 579.869 - Cost per DALY: -3.216   *NA – MRC study*   - IHD events avoided: 80.366 - Stroke events avoided: 128.032 - IHD deaths avoided: 38.372 - Stroke deaths avoided: 51.419   Reducing salt intake in the population with a 5 mmHg reduction in SBP had the greatest population impact and cost-saving to the NHS. | Model based on population survey data from 2003. Assumptions were made that the non-CVD deaths were not affected by the intervention, which is unrealistic. Epidemiological studies used for modelling. Assumed that the interventions had an average effect size over the time horizon of the model | They modelled the impact of scaled-up salt reduction in the whole population, at its lowest a 2 mmHg and at best a 5 mmHg reduction in SBP based on the evidence:  *Dickinson HO, Mason JM, Nicolson DJ, Campbell F, Beyer FR, Cook JV, et al. Lifestyle interventions to reduce raised blood pressure: a systematic review of randomized controlled trials. J Hypertens 2006; 24:215–233.*  *NICE. NICE Clinical Guideline 34: hypertension: management of hypertension in adults in primary care. London: National Institute for Health and Clinical Excellence; 2006.*  *North of England Hypertension Guideline Development Group. Newcastle upon Tyne: Centre for Health Services Research, School of Population and Health Sciences, University of Newcastle upon Tyne; 2004. Report No. 111.*  They also modelled the impact of a reduction in blood pressure in moving from an average current dietary salt intake of between 9 and 10 g to an average intake of 6 g through agreement with the food industry for the whole population based on a summary of the scientific literature by the Medical Research Council (MRC) Human Nutrition Research.  *Jebb S, Mac Evilly C. Why 6 g? A summary of the scientific evidence for the salt intake target. London: Food Standards Agency and Medical Research Council; 2005.* |
| **Study ID** | **Study Design** | **Study Aim** | **Policy(ies) Analysed** | **Participants & sample size** | **Geographical Scope** | **Methods** | **Outcomes** | **Study Limitations** | **Sources referenced & Comments** |
| Gase et al. (2011)^106^ | Modelling study | To examine approaches to reduce sodium content of food served in settings operated or funded by the government of the County of Los Angeles, California | Label, promote, subsidize and provide low-sodium food options | 593 adults (hospital cafeteria)  1820 adults (county government cafeterias) | US | Adapted health impact assessment methods to mathematically simulate various levels of reduction in the sodium content of food served by the County of Los Angeles and to estimate the reductions potential impact on mean systolic blood pressure (SBP) among food-service customers. Data provided by county government food-service vendors used to generate these simulations | **Hospital cafeterias:**   - Average sodium reduction of 773mg/day (23%) - Overall SBP: 1.59   **County government cafeterias:**   - Average sodium reduction of 307mg/day (11%) - Overall SBP: 0.63 | Assumed no major variation in the day to day costumers and linear relationship between sodium consumption and SBP. No data were available on sodium consumption specific to Los Angeles County. They assumed that the prevalence of hypertension among customers in the study mirrored that of the general Los Angeles County population; it does not account for gender and racial/ethnic group differences | **Meal content**  *US Department of Agriculture, Agricultural Research Service. Data tables (PDF) from What we eat in America, NHANES. 2005---2006. Available at: http://www.ars.usda.gov/Services/docs.htm?docid=18349. Accessed May 3, 2011.*  **Reduction intake**  *Schmitz MF, Fielding JE. Point-of-choice nutritional labelling: evaluation in a worksite cafeteria. J Nutr Educ. 1986;18(1):S65---S68.*  *Perlmutter CA, Canter DD, Gregoire MB. Profitability and acceptability of fat- and sodium-modified hot entrees in a worksite cafeteria. J Am Diet Assoc. 1997; 97(4):391---395.*  *Simons-Morton BG, Parcel GS, Baranowski T, Forthofer R, O’Hara NM. Promoting physical activity and a healthful diet among children: results of a school-based intervention study. Am J Public Health. 1991;81(8):986---991.*  *Ellison RC, Capper AL, Goldberg RJ, Witschi JC, Stare FJ. The environmental component: changing school food service to promote cardiovascular health. Health Educ Q. 1989;16(2):285---297.*  **Table salt use**  *Beauchamp GK, Bertino M, Engelman K. Failure to compensate decreased dietary sodium with increased table salt usage. JAMA. 1987;258(22):3275---3278.* |
| **Study ID** | **Study Design** | **Study Aim** | **Policy(ies) Analysed** | **Participants & sample size** | **Geographical Scope** | **Methods** | **Outcomes** | **Study Limitations** | **Sources referenced & Comments** |
| Ha & Chrisholm (2011)^107^ | Modelling study | To assess the costs, health effects and cost-effectiveness of a set of personal and non-personal prevention strategies to reduce CVD in Vietnam, including mass media campaigns for reducing consumption of salt | 1. 1. Mass media campaign 2. 2. Voluntary reformulation |  | Vietnam | WHO-CHOICE methods and analytical models were employed, using local data to estimate the costs, effects and cost-effectiveness of 12 population and individual interventions implemented alone or in combination | *Media salt campaign*   - Cost per year (VND, billion): 89 - DALYs averted per year: 45.939 - VND per DALY saved: 1.945.002   Very cost-effective | Use of local rather than national data on costs and epidemiology of sodium consumption. Also, epidemiology and effect estimates were derived from other international studies and then a panel of experts determined conservative estimates in order to adjust to the context of Vietnam. They did not incorporate in the models other benefits, i.e. decrease in the risk of gastric cancer due to reduced salt intake, or resistance which may be caused by platelet-active drugs | **Mass media campaigns and voluntary reformulation** (20%)  *Asaria P, Chisholm D, Mathers C et al. 2007. Chronic disease prevention: health effects and financial costs of strategies to reduce salt intake and control tobacco use. The Lancet 370: 2044–53*  *He FJ, MacGregor GA. 2002. Effect of modest salt reduction on blood pressure: a meta-analysis of randomized trials. Implications for public health. Journal of Human Hypertension 16: 761–70.*  *Forrester T, Adeyemo A, Soarres-Wynter S et al. 2004. A randomized trial on sodium reduction in two developing countries. Journal of Human Hypertension 19: 55–60.*  *Law MR, Frost CD, Wald NJ. 1991. By how much does dietary salt reduction lower blood pressure? III. Analysis of data from trials of salt reduction. British Medical Journal 302: 819–24*  *Murray CJL, Lauer JA, Hutubessy RCW et al. 2003. Effectiveness and costs of interventions to lower systolic blood pressure and cholesterol: a global and regional analysis on reduction of cardiovascular-disease risk. The Lancet 361: 717–25.*  *Tian HG, Guo ZY, Hu G et al. 1995. Changes in sodium intake and blood pressure in a community-based intervention project in China. Journal of Human Hypertension 9: 959–68* |
| **Study ID** | **Study Design** | **Study Aim** | **Policy(ies) Analysed** | **Participants & sample size** | **Geographical Scope** | **Methods** | **Outcomes** | **Study Limitations** | **Sources referenced & Comments** |
| Barton et al. (2011)^120^ | Modelling study | To estimate the potential cost effectiveness of a population-wide risk factor reduction programme aimed at preventing cardiovascular disease | 1. Salt legislation | Entire population aged between 40 and 79 years old | England and Wales | Spreadsheet model carrying a range of possible interventions to quantify the reduction in CVD over a decade, assuming the benefits apply consistently for men and women across age and risk groups | Dietary salt intake in England and Wales averages approximately 8.5 g/day. A reduction of 3 g/day in salt intake represents a conservative estimate of the potential effects of specific legislation, based on the 6 g reduction achieved in Finland, Japan, and elsewhere. Reducing salt intake by 3 g/day might reduce mean population systolic blood pressure by approximately 2.5 mm Hg. This would equate to a 2% decrease in the risk reduction model. This would prevent approximately 4450 deaths from cardiovascular disease, with total discounted savings overall of approximately £347m over a decade, representing equivalent annual savings of approximately £40m | They made no attempt to consider recurrent events or subsequent deaths. The estimates of deaths avoided, life years gained, and cost savings are thus likely to be underestimates, making the analysis somewhat conservative. The 10 year time frame for prevention of cases; gains over a lifetime would clearly be greater. The analysis was pragmatically limited to people aged between 40 and 79 years at the time of the intervention. This initial modelling lacks a full probabilistic sensitivity analysis | **Referenced 3g/day from:** *He FJ, MacGregor GA. A comprehensive review on salt and health and current experience of worldwide salt reduction programmes. J Hum Hypertens 2009;23:363-84* |
