# Supplementary material for: Systematic review of dietary salt reduction policies: Evidence for an effectiveness hierarchy?
Source: PLoS One. 2017 May 18;12(5):e0177535. doi: 10.1371/journal.pone.0177535 (PMC5436672; doi:10.1371/journal.pone.0177535)
Supplement: S1 File — (DOCX) [file pone.0177535.s003.docx]

**Supplementary file 2.**

**PROTOCOL**

**Systematic review of dietary salt reduction policies: evidence for an effectiveness hierarchy?**

Lirije Hyseni, Alex Elliot Green, Ffion Lloyd-Williams, Martin O’Flaherty, Chris Kypridemos, Rory McGill, Lois Orton, Helen Bromley, Francesco P Cappuccio, Simon Capewell

**AIM**

To critically review and summarise the growing evidence for a public health ‘effectiveness hierarchy’, in order to inform future preventive health strategies focused on dietary salt.

**Background**

An effectiveness hierarchy for preventive interventions may exist, whereby approaches targeting individuals consistently achieve a smaller public health impact than policies such as regulations or taxes that affect the whole population.

**Criteria for considering studies for this review**

**Types of studies**

Systematic reviews and primary studies reporting a quantitative assessment of the effects (intended or unintended) of actions to improve healthy lifestyle.

Include: Primary studies RCTs, Systematic Reviews (SRs), empirical observational studies, natural experiments, modelling studies, secondary analysis, and before vs. after interventions

Papers from the last 30 years, i.e. 1985 onwards are considered for inclusion. Non-systematic reviews will be used as a source of primary studies where systematic reviews do not exist or are out of date

Exclude: commentary/opinion articles; purely qualitative evaluations with no quantitative assessment; data/statistics from monitoring and surveillance that are not directly linked to a policy intervention; reviews/studies of under-nutrition or under-weight.

**Types of participants**

Include: studies for all age groups from all populations, from high-, middle- and low-income countries. Exclude: animal studies and studies involving pregnant women?

**Types of interventions**

Interventions/policies (upstream and downstream) focused on dietary salt.

Actions must involve an intervention, policy, programme, pledge or strategy to promote a reduction in salt intake.

Include: SRs and Primary studies evaluating the effects of actions to promote these specific healthy behaviours by government policy or adopted in specific real or experimental settings.

Exclude: Only qualitative data, with no quantified changes.

**Types of comparison**

Include: systematic and non-systematic reviews where actions to promote salt reduction were evaluated or compared.

Exclude: no comparisons of different actions to promote salt reduction presented

**Types of outcome measures**

Primary outcome of interest is quantified changes in dietary intake salt (g/day).

Secondary outcomes include changes in clinical/physiological indicators related to NCDs, QALYs, DALYs and behaviours associated with a healthy lifestyle.

Exclude: healthy lifestyle knowledge or self-efficacy/skills/competency; process evaluations reporting on implementation of interventions/policies without any outcome data; data only on costs, feasibility or acceptability without an assessment of primary effects (intake); studies on individuals as opposed to groups or whole populations; under-nutrition; under-weight; BMI.

**Search strategy for identification of studies**

Stage One: Pilot study. The approach described below will be tested for one target behaviour, dietary salt.

Stage Two: **Key informants** will be asked to highlight key studies for the range of actions for each life style factor: dietary intake, smoking/tobacco and alcohol consumption and physical activity level. In addition, key websites will be screened, including: the Cochrane Collaboration, the Campbell Collaboration, the Centre for Reviews and Dissemination, NICE, UKHF PIE, the UK NF infoSource, the European Heart Network, the World Health Organisation (including PAHO and ECHP), the World Bank, the Kings Fund, NOPA and Bandolier.

Stage three: Once key studies have been identified for each action and life style factor, these papers will be used to identify terms to be used in targeted searches of the following electronic databases: MEDLINE; SCI; SCOPUS; PsychInfo, CDSR; The Campbell Library; CRD Wider Public Health database. Targeted searches will also be conducted in Google Scholar. Reference lists of included articles will also be scanned. Zotero will be used to manage the references.

**Screening of potentially eligible studies**

LH and AEG will screen titles and abstracts of all items retrieved to identify potentially eligible studies based on the inclusion/exclusion criteria. All articles deemed potentially eligible will be retrieved in full text. Full text articles will be screened for inclusion by LH and SC based on the inclusion/exclusion criteria, above. Disagreement will be resolved by discussion, or referral to a third party (SC).

**Data extraction**

Data will be extracted into pre-designed and pre-piloted forms. Data to be extracted include: first author; year of publication; funder(s); study aim(s); sample size; study design; methods; participants; geographical scope; policies analysed; length of follow-up; outcomes (effect/response); limitations and potential risks of bias. We may contact study authors for unclear, missing or additional data.

**Assessing the strength of evidence**

We will employ the typology of evidence proposed by Petticrew and Roberts (2003). This typology will be developed by matching the research objectives to specific types of research in a matrix. We will also consider using the following factors (from GRADE 2012):

Consistency/inconsistency between studies; Methodological quality/study limitations; Integrity/fidelity (completeness of implementation); and Indirectness of evidence (reflecting study design).

*[We note the alternative Cochrane Collaboration hierarchy of study designs for evaluating the effects of public health and health promotion interventions (Armstrong and Walters 2007): RCTs>Non-RCTs>Controlled Before and After studies (CBAs)>Interrupted Time Series (ITSs) and Comparisons with historical controls or national trends. However, such hierarchies are often considered inappropriate as they may in fact be reversed when answering certain public health questions (Petticrew and Roberts 2003)].*

**Data synthesis**

The data from included studies will be synthesised as a narrative review (Mays 2005; Popay 2003):

Data will be analysed thematically.

- Data will be organised by healthy eating intervention/action and strength of evidence.
- Reasons for contradictory findings will be explored.
- Data will be presented narratively, with tables and graphical displays (where appropriate).

**Dissemination of results**

Via conferences, websites, abstracts, and peer reviewed papers.

# References

Armstrong R, Walters E (on behalf of the Guidelines for Systematic Reviews in Health Promotion and Public Health Taskforce). 2007. Systematic Reviews of Health Promotion and Public Health Interventions. Available from: http://ph.cochrane.org/sites/ph.cochrane.org/files/uploads/Guidelines%20HP_PH%20reviews.pdf.

GRADE working group. 2012. Accessed at: http://www.gradeworkinggroup.org/index.htm.

Mays N, Pope C, Popay J. 2005. Systematically reviewing qualitative and quantitative evidence to inform management and policy-making in the health field. Journal of Health Services Research and Policy 10(1)S1:6 – S1:20.

Petticrew M, Roberts H. 2003. Evidence, hierarchies, and typologies: horses for courses. JECH 57: 527-529.

Popay J, Baldwin S, Arai L, Britten N, Petticrew M, Rogers M, Sowden A. Narrative Synthesis in Systematic Reviews. [ESRC Methods Briefings](http://www.ccsr.ac.uk/methods/publications/); 22. 2003.

***LH & SC 4 May 2015***

**POTENTIAL TOPIC EXPERTS**

**Dietary Salt**

Graham MacGregor, Feng He, Franco Cappuccio
